# Profiling the T Cell Receptor Alpha/Delta Locus in Salmonids

**DOI:** 10.3389/fimmu.2021.753960

**Published:** 2021-10-18

**Authors:** Eva-Stina Edholm, Christopher Graham Fenton, Stanislas Mondot, Ruth H. Paulssen, Marie-Paule Lefranc, Pierre Boudinot, Susana Magadan

**Affiliations:** ^1^ Faculty of Biosciences, Fisheries & Economics, Norwegian College of Fishery Science, University of Tromsø—The Arctic University of Norway, Tromsø, Norway; ^2^ Clinical Bioinformatics Research Group, Genomics Support Centre Tromsø (GSCT), Department of Clinical Medicine, Faculty of Health Sciences, University of Tromsø - The Arctic University of Norway, Tromsø, Norway; ^3^ Université Paris-Saclay, INRAE, AgroParisTech, Micalis Institute, Jouy-en-Josas, France; ^4^ GABI, INRA, AgroParisTech, Université Paris-Saclay, Jouy-en-Josas, France; ^5^ IMGT^®^, The International ImMunoGeneTics Information System (IMGT), Laboratoire d´ImmunoGénétique Moléculaire (LIGM), Institut de Génétique Humaine (IGH), CNRS, University of Montpellier, Montpellier Cedex, France; ^6^ Université Paris Saclay, INRAE, UVSQ, Virologie et Immunologie Moléculaires, Jouy-en-Josas, France; ^7^ Immunology Laboratory, Biomedical Research Center (CINBIO), University of Vigo, Vigo, Spain; ^8^ Galicia Sur Health Research Institute (IIS-GS), Hospital Alvaro Cunqueiro, Vigo, Spain

**Keywords:** T cell receptor, repertoire, salmonid fish, VDJ annotation, TRA/TRD locus, gene rearrangement, adaptive immunity

## Abstract

In jawed vertebrates, two major T cell populations have been characterized. They are defined as α/β or γ/δ T cells, based on the expressed T cell receptor. Salmonids (family *Salmonidae*) include two key teleost species for aquaculture, rainbow trout (*Oncorhynchus mykiss*) and Atlantic salmon (*Salmo salar)* which constitute important models for fish immunology and important targets for vaccine development. The growing interest to decipher the dynamics of adaptive immune responses against pathogens or vaccines has resulted in recent efforts to sequence the immunoglobulin (IG) or antibodies and T cell receptor (TR) repertoire in these species. In this context, establishing a comprehensive and coherent locus annotation is the fundamental basis for the analysis of high-throughput repertoire sequencing data. We therefore decided to revisit the description and annotation of TRA/TRD locus in Atlantic salmon and two strains of rainbow trout (Swanson and Arlee) using the now available high-quality genome assemblies. Phylogenetic analysis of functional TRA/TRD V genes from these three genomes led to the definition of 25 subgroups shared by both species, some with particular feature. A total of 128 TRAJ genes were identified in *Salmo*, the majority with a close counterpart in *Oncorhynchus*. Analysis of expressed TRA repertoire indicates that most TRAV gene subgroups are expressed at mucosal and systemic level. The present work on TRA/TRD locus annotation along with the analysis of TRA repertoire sequencing data show the feasibility and advantages of a common salmonid TRA/TRD nomenclature that allows an accurate annotation and analysis of high-throughput sequencing results, across salmonid T cell subsets.

## Introduction

The emergence of vertebrates was accompanied by morphological and functional innovations, such as the development of an adaptive immune system (1, 2). Vertebrate adaptive immunity relies on the clonal expression of somatically diversifying antigen receptors on lymphocytes, which generates diversity and antigen specific recognition far beyond those offered by the allelic variation or alternative splicing processes. In jawed vertebrates, including jawed fish, the adaptive immune components include B and T lymphocytes, which express specific antigen receptors, the immunoglobulins (IG) or antibodies and T cell receptors (TR), respectively (3–5).

TR can be formed by two distinct polypeptide chains, α/β or γ/δ, which distinguish the two major T cell populations across jawed vertebrates (4, 6–8). The germline structure of TR loci provides the basic pieces from which genes encoding these receptors are assembled to generate a vast repertoire of T cells bearing structurally diverse receptors (potentially >10^13^ sequences in humans) for specific antigen recognition (4, 6). During T cell development, TR genes, called variable (V), diversity (D), and joining (J) undergo somatic rearrangement mediated by RAG1/2 and other enzymes to encode the different chains of the receptor. The V-(D)-J combinations made from the germline gene pool generate the wide diversity of TR chains required for antigen recognition.

TR locus organization has been analyzed in many mammalian species (4, 9–15) but the data available for teleost and lower ectothermic vertebrates are much more limited (9, 11, 16, 17). The TRB locus has a common genomic structure in several mammalian species, with different numbers of TRBV genes positioned upstream of tandem-aligned TRBD-J-C clusters, each composed of a single D (TRBD) gene, several J (TRBJ) genes, and one C (TRBC) gene (4, 10, 13, 18, 19). The TRG locus shows greater gene organization plasticity. Generally, the TRG genes comprise an array of TRGV genes linked to TRGJ and TRGC genes organized in J-C clusters (4, 9, 11). In all studied vertebrates including several teleost species, the TRA and TRD genes present a conserved linkage in a unique TRA/TRD locus (4, 15, 20–23). The TRA/TRD locus described in the amphibian *Xenopus laevis* is highly conserved among tetrapods (15, 24). The TRD locus is imbedded within the TRA locus with the following arrangement: TRA/TRD V-TRDD-TRDJ-TRDC-TRAJ-TRAC, with some V genes being used in the synthesis of both α and δ TR chains (9, 11, 21, 24). Furthermore, the presence of IGHV-related genes within the TRA/TRD locus, which are expressed exclusively with δ TR chains, has been also reported in different tetrapods, including Xenopus and the Chinese alligator (24, 25). The organization of the TRA/TRD locus in teleosts, based on data from zebrafish (*Danio rerio*) (17), Japanese pufferfish (*Takifugu rubripes*) (26), green pufferfish (*Tetraodon nigroviridis*) (20) and Atlantic salmon (*Salmon salar*) (23) is different. On the chromosome in the Watson orientation (FWD5’>FWD3’), the multiple TRA/TRD V genes are followed by the TRAC-TRAJ-TRDC-TRDJ-TRDD cluster, which is in inverted orientation to the TRA/TRD V genes. Defining the locus orientation, by the transcriptional orientation of the TRDC and TRAC genes, the teleost TRA/TDR locus is in reverse orientation (REV) on the chromosome, with the TRDD genes at the 5’ end of the locus and the TRA/TRD V genes translocated downstream at the 3’ end of the locus in opposite orientation (IMGT index > Genomic orientation, http://www.imgt.org/IMGTindex/genomicOrientation.php). TRA and/or TRD genes have been identified in other teleosts, such as channel catfish (*Ictalurus punctatus*) (16), mandarin fish (*Siniperca chuatsi*) (27), sea bass (*Dicentrachus labrax*) (28), common carp (*Cyprinus carpio*) (29), and flounder (*Parlichthys olivaceus*) (30), but the organization of genomic locus has yet to be characterized in these species.

Salmonids (family *Salmonidae*) include two key species for aquaculture, rainbow trout (*Oncorhynchus mykiss*) and Atlantic salmon (*Salmo salar)* which constitute important models for fish immunology and important targets for vaccine development. While TRA and TRB chain cDNAs were initially cloned in rainbow trout (31, 32), a first description of the organization of the TRA/TRD locus was reported from Atlantic salmon by Yazawa et al. (23). On *Salmo salar* chromosome 14 genomic sequence, a total of 292 TRA/TRD V genes (128 functional) were identified upstream of the TRAC-TRAJ-TRDC-TRDJ-TRDD cluster, which is in inverted orientation to TRA/TRD V genes. One hundred and twenty two TRAJ genes (113 functional), one TRDJ and 3 TRDD genes were also found in the locus (23).

In this study, we re-examined the genomic sequence of the TRA/TRD locus in Atlantic salmon and rainbow trout and revisited its annotation to establish a universal salmonid nomenclature based on IMGT rules and standards (4, 33). Recent progress in sequencing technologies has led to much improved versions of genome assemblies for these species. In rainbow trout, homozygous clonal lines from two different strains have been sequenced. The first reference genome, the Omyk_1.0 assembly, was from the line “Swanson”, which was derived from a semi-wild population from the Swanson River in the Kenai Peninsula of Alaska (34). The second assembly (released 2020, USDA_OmykA_1.1) was generated from the Arlee clonal line (35), which derives from the “Arlee” strain. This domesticated hatchery strain was used by the Montana Department of Fish, Wildlife and Parks and is thought to have originally been collected from Northern California like most farmed rainbow trout stocks that were imported to Europe (Gary Thorgaard, Personal Communication). Focusing on functional sequences, phylogenetic analysis of TRA/TRD V genes from these three genomes led to the definition of 25 subgroups shared by *Salmo* and *Oncorhynchus*, and an additional single gene subgroup present only in *Oncorhynchus.* Maps from Atlantic salmon and rainbow trout assemblies were established and compared, which will facilitate updates of the nomenclature based on future sequence data. In addition, this work on TRA/TRD locus allows accurate annotation and analysis of high-throughput sequencing repertoire data, across salmonid T cell subsets.

## Material And Methods

### Annotation of TRA/TRD Loci

Atlantic salmon (ICSASG_v2; GenBank GCA_000233375.4, isolate Sally, female, breed: double haploid), rainbow trout Arlee (USDA_OmykA_1.1; GenBank GCA_013265735.3, isolate Arlee, male) and Swanson (Omyk_1.0; GenBank GCA_002163495.1, isolate Swanson, male) genome assemblies were accessed through the NCBI website (https://www.ncbi.nlm.nih.gov/). To identify the chromosomes that embrace the TRA/TRD locus, previously published salmonid TRAC and TRDC sequences (23, 31, 32, 36, 37) were used for BLASTn and tBLASTn searches. The TRA/TRD locus was located on chromosome 14, in Atlantic salmon (CM003292.1, NC_027313.1, 93901823 bp), and on chromosome 8 in rainbow trout Arlee (CM023226.2, NC_048572.1, 91622588 bp) and Swanson (CM007942.1, NC_035084.1, 83778284 bp). These chromosomes were selected for in depth analysis. TR genes were searched using the BLAST tool at Galaxy website (https://usegalaxy.org) and visual analysis using SnapGene software (from Insightful Science; available at snapgene.com). Previously published TRA and TRD constant sequences from rainbow trout and Atlantic salmon (23, 31, 32, 36, 37) were used as queries to identify the chromosomal regions containing TRAC and TRDC genes, intron splice signals were identified for all sequences and were used to determine the limits of the TRAC and TRDC coding exons. To annotate TR variable V, joining J, and diversity D genes, the recombinant signal (RS) sequences were identified, respectively V-RS, J-RS, and 5’D-RS and 3’D-RS. Splice signals were used to determine the limits of the coding nucleotide sequences for the V (L-PART1 donor splice and L-PART2 acceptor splice) and the J (J-REGION donor splice). The annotation and functionality of TRA/TRD genes were established according to IMGT rules and standards (4, 33). In silico analysis of gene function considered the following parameters: a) presence of appropriate splice sites, b) presence of RS sequence compatible with effective rearrangement, c) open reading frames which included conserved cysteine and tryptophan codons at positions 23 (1st-CYS), 41 (CONSERVED-TRP) and 104 (2nd-CYS) of TRA/TRD V genes (IMGT unique numbering system) (32). Briefly, a germline entity (TRA or TRD V, TRDD, TRDJ or TRAJ gene) was considered functional (F) if the coding region has an open reading frame, no defect in splicing site, or in RS and presents key conserved amino acids; as open reading frame (ORF) if the coding region has an open reading frame but there are defects in the splicing sites or RS sequences, and/or changes of conserved amino acids that lead to incorrect folding; or annotated as pseudogene (P) if the coding region has stop codon or frameshift mutations (4, 33).

#### Nomenclature Rules

Salmonid TRA/TRD V genes were named based on nucleotide similarity, phylogenetic analysis and positional information. We followed the same principles as in our recent work on salmonid IGH loci (38, 39). The proposed TRAV names are constituted as follows: first, TRAV, then the subgroup number, then a first dash and a number defining the gene rank in the locus (4, 33). For example, the name TRAV2-2 denotes a gene belonging to the subgroup 2, located at the reference rank 2 within the locus. This rank was determined by the locus 5’-3’ transcriptional orientation of the TRDD-TRDJ-TRDC-TRAJ-TRAC cluster. Additional numeric qualifiers could be added when relevant to indicate the position of a gene in a microcluster, as described in (39). Accordingly, we first classified and named the Atlantic salmon TRAV genes. We then mapped rainbow trout TRA/TRD V genes to the corresponding subgroups based on a threshold off 75% nucleotide identity and named them based on positional information. This name format was chosen as it provides flexibility to integrate additional genes, and/or describe gaps while keeping the nomenclature consistent across haplotypes or genomes updates (39). There is no criterion to differentiate TRAV and TRDV a priori. Our choice of naming genes “TRAV” was made for the sake of coherence and standardization (according to the IMGT Scientific chart rules) and has been validated by the IUIS_ Nomenclature Committee. The expression of genes by TR alpha chains is documented by our repertoire sequencing data, and does not preclude of the expression by TR delta chain. The TRAJ genes were named following the same schedule proposed for human TRA/TRD locus (4, 40, 41), with TRAJ1 being the closest TRAJ to TRAC gene. This annotation also fits with the previous description given in Atlantic salmon by Yazawa et al. (23). Chromosomal coordinates given in the TR gene annotation files (**Supplementary Data 1**) include the recombinant signal (RS) sequence and coding regions of TRAV,TRAJ, TRDJ and TRDD genes, and refer to the genomic region that contains the 3 exons of the TRAC and TRDC genes (4).

### Phylogenetic Analysis

Phylogenetic analysis was performed on functional TRAV sequences from Atlantic salmon and rainbow trout. Amino acid sequences were aligned using ClustalW in the MEGA-X software (42). The phylogenetic trees were then constructed using the Maximum Likelihood method in MEGA X, corrected using the Poisson model, and bootstrapped 500 times.

### Samples and RNA Extraction for Sequencing of Atlantic Salmon TRA Transcripts

Head kindey (HK), spleen and gill were collected from three non-vaccinated Atlantic salmon, presmolts, strain NLA reared in hatchery at the Kårvika Aquaculture Research station, Troms, Norway and confirmed free of the salmon pathogens ISAV, SAV, PRV and IPNV by RT-qPCR. They were kept in running freshwater at 10°C, exposed to continuous light and fed with commercial dry feed. Tissues were aseptically harvested, kept in RNALater at -20°C until use and total RNA was extracted using the RNeasy Mini Kit (Qiagen). All animal handling were performed in accordance with the recommendations of the current animal welfare regulations: FOR-1996-01-15-23 (Norway).

### DNA Library Preparation and Sequencing

Reverse transcription (RT) was carried out using the SMARTer RACE 5`/3`kit (Clontech). Briefly, first strand synthesis was performed following the manufacturers protocol using 0.5 μg of total RNA per reaction. Then, double-stranded cDNA was obtained with PCR assay carried out using 2.5 μL of diluted RT product according to the manufacturers protocol using a TRAC reverse (GSP1) primer (5`-GATTACGCCAAGCTTGGCAAGCTGTGGTATTGCTTGAGTTC-3`). Touchdown PCR cycling conditions were performed as follows: five cycles at 94°C for 30 s and 72°C for 2 min; five cycles at 94°C for 30 s, 70°C for 30 s, and 72°C for 2 min; 25 cycles at 94°C for 30 s, 68°C for 30 s, and 72°C for 2 min; and a final step at 72°C for 10 min. The resulting PCR reaction was run on a 1,2% agarose/EtBr gel and the resulting ~500bp single band was extracted and gel purified using the NucleoSpin Gel and PCR Clean-Up Kit (Clontech).

The amount of input DNA for library preparation was quantified using a Qubit-fluorometer 3.0 (Invitrogen). Libraries were prepared using the Nextera XT DNA Sample Preparation Kit (Illumina Inc., San Diego, CA, USA) using the standard protocol. Each library was then quality checked with the Agilent 2100 Bioanalyzer. Prior to pooling and sequencing, libraries were quantified through PCR using the KAPA Library Quantification Kits, according to the manufacturer’s instructions. Libraries were pooled (4nM) and were diluted to 1.8 pM and subsequently paired- end sequenced with an Illumina Next Seq550 platform and with 300 cycle run chemistry.

### Raw Data Processing

All forward and reverse illumina reads were merged using the flash software (43) together to produce extended fasta reads. Only extended reads greater than 150 bp were considered. Using the seqkit software (44) all reads were relabeled with their respective sample name. The reads were then concatenated into one large fasta file. A blast database was constructed from the resultant sample labeled extended fragment file with the makeblastdb command from the ncbi blast executables. The C region sequence, J region sequences and V region sequences were then blasted against the locally created blastdb database. Only sequences with a 97% identity were considered as valid C, J, or V hits. The R data.table package was used to load the blast result files for C, J, and V. Viable blast ids met the following criteria; a C region greater than 30 bp, J region with an identity greater than 99.9%, V region with an identity greater than 99.9% and a length greater than or equal to 50 bp. Remaining V and J blast hits were then sorted by expectancy and any duplicates were removed. The remaining valid blast ids contain a C region, J region, and V region meeting the above criteria. The extended and labeled fasta sequence could be retrieved by blast_ids. Each sequence was translated in all six reading frames to define productive and unproductive sequences. To account for variation with respect to library size samples were normalized using the rlog function of the DESeq2 package to transforms the count data to the log2 scale.

## Results

### Salmonids TRA/TRD Genomic Organization: Comparison Between Atlantic Salmon and Rainbow Trout

#### The Atlantic Salmon TRA/TRD Locus

**Figure 1:**
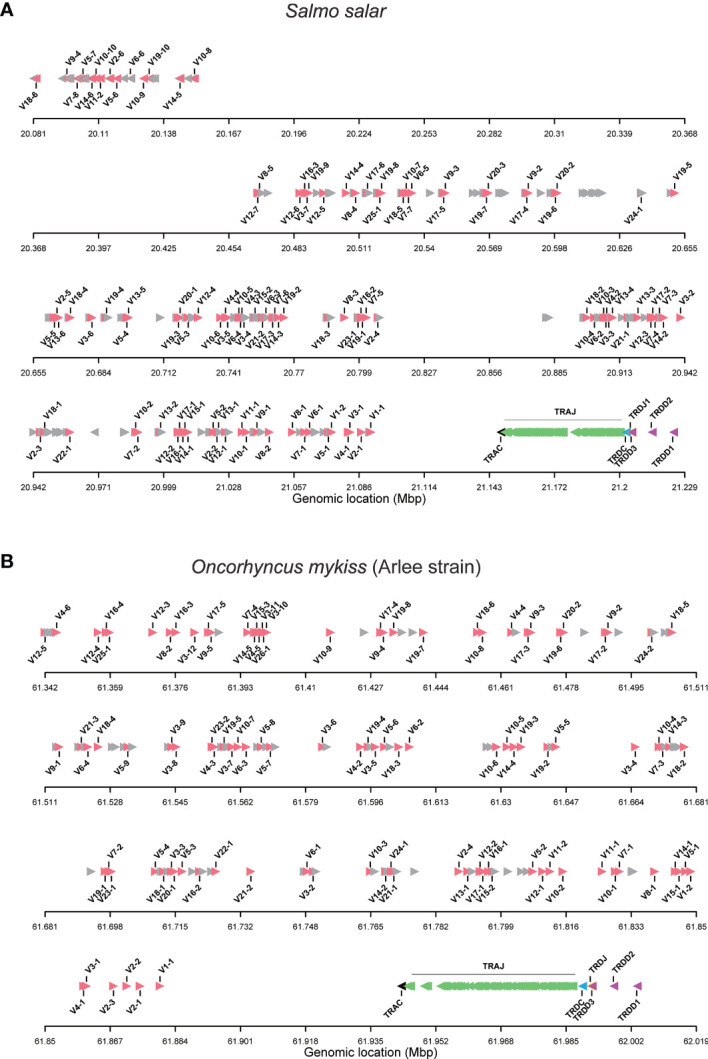
Organization of TRA/TRD locus in salmonids. **(A)** Atlantic salmon (*Salmo salar*) and **(B)** rainbow trout (*Onchorynchus mykiss*, Arlee strain). TRAV gene names are according to IMGT nomenclature (4, 33). Functional and ORF TRAV genes are in red. TRAV pseudogenes with frameshift(s) in the V-REGION are in grey and their name are not displayed. The arrow indicates the orientation. The symbols representing the genes are not to scale. Information about gaps (length and location) is available in **Supplementary Data 3**.

The TRA/TRD locus of Atlantic salmon was manually annotated using the current genome assembly [ICSASG_v2; GenBank GCA_000233375.4], as well as cDNA data available in GenBank and sequences previously reported by Yazawa et al. (23). The TRA/TRD locus is located on chromosome 14, in forward (Watson) orientation, defined from the FWD5’ (short arm) telomeric end to the FWD3’ (long arm) telomeric end of the chromosome (**Figure 1A**). It spans 1,14 Megabases (Mb) and includes, from FWD5’ to FWD3’, 239 TRAV, one TRAC, 128 TRAJ, one TRDC, a single TRDJ and three TRDD (**Figure 1A**). Defining the *Salmo salar* TRA/TRD locus orientation, by the transcriptional orientation of the TRAC and TRDC genes, the locus is in reverse orientation (REV) on chromosome 14, with the TRDD genes at the 5’ end of the locus and the TRAV genes translocated downstream at the 3’ end of the locus in opposite orientation. Thus, the Atlantic salmon TRA/TRD locus comprises from 5’ (chr FWD3’) to 3’ (chr FWD5’), first the TRDD-TRDJ-TRDC-TRAJ-TRAC cluster followed by the TRA/TRD V genes translocated downstream in 3’ in opposite orientation.

On the chromosomal FWD5’ side of the region containing V genes, a first block of 29 TRAV genes is in inverted orientation. It is followed by a region > 300 kilobases (Kb), between the TRAV10-8 and the TRAV12-7 genes, where we could not identify any TRAV genes and there are no gaps (**Figure 1A**). The other 210 TRAV genes are in forward orientation on the chromosome. In conclusion, we identified a total of 239 TRAV genes (**Supplementary Data 1**), while Yazawa et al. (23) reported 292 TRA/DV genes.

**Figure 2:**
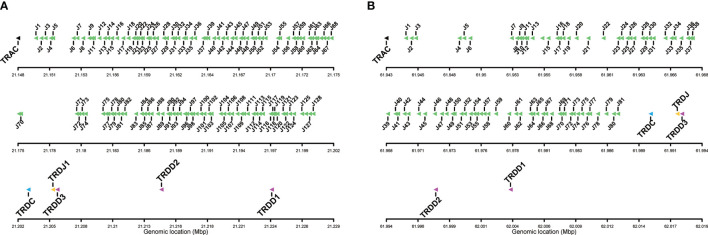
Detailed view of the TRAJ cluster and the TRD genes, including TRDC, TRDJ and TRDD genes. **(A)** *Salmo salar* and **(B)** *Oncorhynchus mykiss*, Arlee line. The boxes representing the genes are not to scale.

The TRAJ-TRAC cluster spans about 52 Kb. The exon-intron organization of the TRAC gene was confirmed, comprising 3 exons that encode a protein of 110 amino acids (AA). A total of 128 TRAJ genes are found on the genomic region between the TRAC and TRDC genes (**Figures 1A**, **2A**), *i.e.* 6 more genes than previously identified (23). The TRD genes (TRDD, TRDJ and TRDC) correspond to those previously characterized by Yazawa et al. (23), with three TRDD genes, one TRDJ and one TRDC gene that comprises 3 exons.

#### The Rainbow Trout TRA/TRD Locus

The analysis of genome assemblies from isogenic fish from the Swanson (Omyk_1.0; GCA_002163495.1) and Arlee (USDA_OmykA_1.1; GCA_013265735.3) strains resulted in a first description of the TRA/TRD locus in rainbow trout. In both genome assemblies the TRA/TRD genes are located on chromosome 8, with a similar FWD5’ to FWD3’ configuration (TRA/TRD V followed by a TRAC-TRAJ-TRDC-TRDJ-TRDD cluster comprising genes in inverted orientation) as described for Atlantic salmon (**Figure 1B** and **Supplementary Data 2**). Defining the *Oncorhychus mykiss* TRA/TRD locus orientation, by the transcriptional orientation of the TRAC and TRDC genes, the trout locus on chromosome 8 is, like that of salmon on chromosome 14, in reverse orientation (REV), with the TRDD genes at the 5’ end of the locus and the TRAV genes translocated downstream at the 3’ end of the locus in opposite orientation. Thus, the *Oncorhychus mykiss* TRA/TRD locus comprises from 5’ (chr FWD3’) to 3’ (chr FWD5’), first the cluster TRDD-TRDJ-TRDC-TRAJ-TRAC cluster followed by the TRA/TRD V genes translocated downstream in 3’ in opposite orientation as described for Atlantic salmon.

Unlike what was identified in Atlantic salmon TRA/TRD locus, in rainbow trout all annotated TRAV genes are in the same opposite orientation (**Figures 1A, B** **)**. The TRA/TRD locus spans a total length of approximately 825Kb in Omyk_1.0 genome (Swanson strain), compared to only 660Kb in the USDA_OmykA_1.1 genome (Arlee strain). Also, the number of annotated TRAV and TRAJ genes differs significantly between assemblies. Thus, 164 TRAV genes were found in Arlee for only 57 in Swanson (**Supplementary Data 1**), and 81 TRAJ genes were annotated in Arlee compared to 66 in Swanson (**Supplementary Data 2** and **Figure 2B**). These discrepancies may be due to either problems in the genome assembly or real differences between the loci of the different rainbow trout strains. Further studies are required to elucidate this point. However, the presence of gaps in the Swanson TRA/TRD locus suggests that the assembly of this region may be incorrect (**Figure 1B** and **Supplementary Data 2**). TRAJ genes were classified in accordance with their position and their high nucleotide identity with their corresponding Atlantic salmon orthologues.

### Salmonid TRAV Genes

A total of 239 TRAV genes were identified in the Atlantic salmon TRA/TRD locus. Of these, 104 TRAV are functional (F), 12 TRAV are ORF, and 123 TRAV are pseudogenes. The rainbow trout Arlee TRA/TRD locus comprises 163 TRAV genes: 93 TRAV are functional, 14 TRAV are ORF and 56 TRAV are pseudogenes (**Figure 1B** and **Supplementary Data 1**), while in Swanson assembly only 57 TRAV genes could be annoted. Of these 25 TRAV-are functional, 6 TRAV are ORF and 26 TRAV are pseudogenes (**Supplementary Data 1 **and** Data 2**).

**Figure 3:**
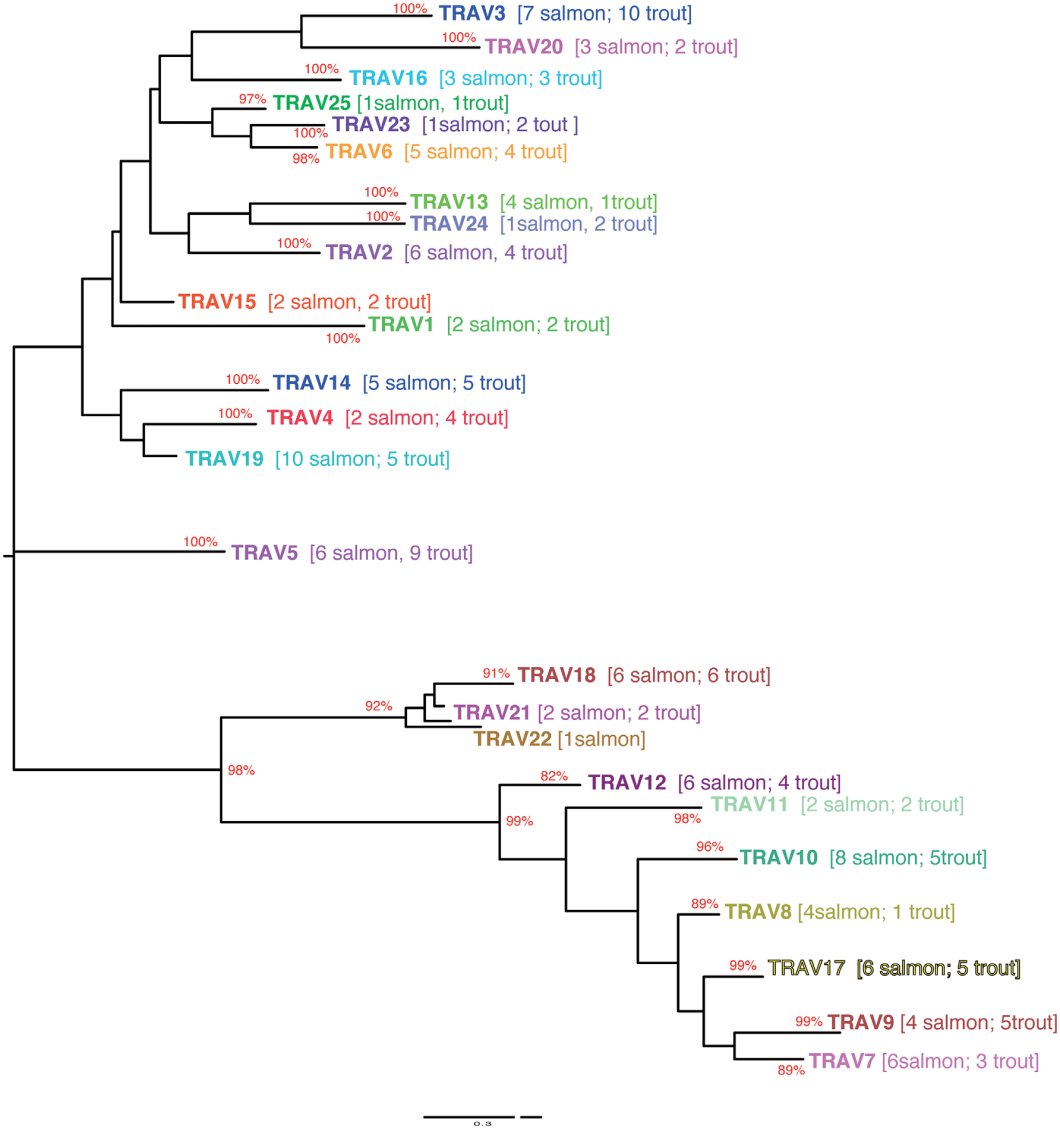
Evolutionary tree of TRAV protein sequences from Atlantic salmon and rainbow trout. The tree was inferred using the Maximum Likelyhood method and JTT matrix based model. A boostrap of 500 replicates was used. The tree with highest likelihood is shown, and is drawn to scale, with branch lengths representing the number of substitutions per site. The percentage of trees in which the associated taxa cluster together is indicated when >80. Subgroups have been collapsed for the sake of readability. The number of Atlantic salmon and rainbow trout sequences in each subgroup is indicated between brackets (an extended tree is represented in **Supplementary Figure 4**). The analysis has been performed only on functional V sequences. Hence it comprises only 25 subgroups since subgroup 26 is only represented by an ORF sequence in the Arlee rainbow trout genome.

**Figure 4:**
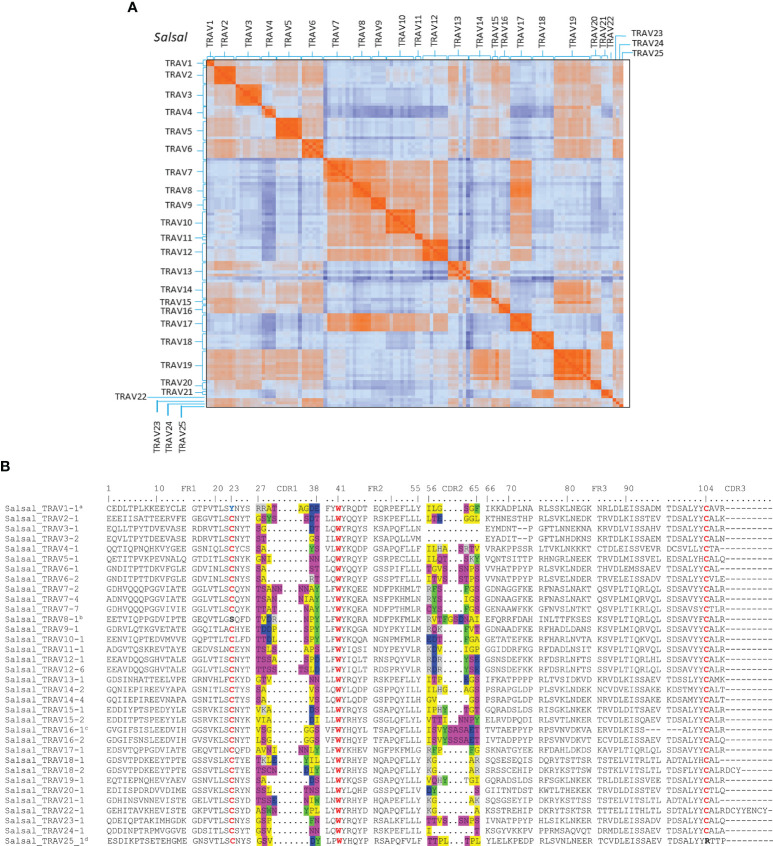
Comparison of TRAV sequences from Atlantic salmon. **(A)** Heatmap representation of pairwise distance matrix of functional and ORF TRAV. Pairwise distances were estimated from amino acid alignments using the Poisson model. 103 functional TRAV and 12 ORF (TRAV4-2, TRAV4-4, TRAV5-7, TRAV6-3, TRAV7-1, TRAV8-5, TRAV10-3, TRAV10-5, TRAV12-3, TRAV13-4, TRAV13-6 and TRAV14-1) were included in the analysis. Co**l**or in each pixel indicate pairwise distance among and between each TRAV sequence. **(B)** Multiple Alignment of representative TRAV subgroups for Atlantic salmon (*‘*Salsal’ in the IMGT 6-letter abbreviation for genus and species). The CDR1 and CDR2 encoded amino acids are colored as follows: *gray* positively charged R groups, *blue* negatively charged R groups, *green* aromatic R groups, *pink *polar uncharged R groups, *yellow *nonpolar R groups, *red *conserved cysteines (1st-CYS 23 and 2nd-CYS 104) and conserved tryptophan 41. *^a^* Conserved 1st-CYS 23 is replaced by a Tyrosine (Y). *^b^* Conserved 1st-CYS 23 is replaced by a Serine (S). *^c^* six amino-acid deletion in FR3 compared to the other genes of the subgroup. *^d^* Conserved 2nd-CYS 104 is replaced by a Arg.

Based on the percentage of identity between nucleotide sequences of the V-REGION (threshold 75%), Atlantic salmon F and ORF TRAV genes (116 TRAV sequences) could be classified into 25 TRAV gene subgroups (**Table 1** **).** All F and ORF TRAV genes identified in both lines of rainbow trout could be classified into the same 25 subgroups defined for the Atlantic salmon TRAV, except for one ORF TRAV gene (TRAV26) from the Arlee assembly, which represents a single gene subgroup (**Table 1**). To confirm the subgroup clustering deduced amino acid sequences of F and ORF TRAV genes were further analyzed. Both phylogenetic trees of functional TRAV genes (**Figure 3** and **Supplementary Data 4**) and the pairwise distance matrix (**Figure 4A** and **Supplementary Data 5**) generated from F and ORF TRAV sequences support these 26 subgroups. Representative Atlantic salmon and rainbow trout (Arlee) amino acid sequences from each TRAV subgroup were aligned with sequences from the 75 zebrafish TRAV subgroups defined by Seeley et al. (17) and a phylogenetic tree was constructed using NJ method (**Supplementary Data 6**). We chose the zebrafish because it was the only other teleost species in which a comprehensive analysis of the TRA/DV repertoire had been reported and was available in IMGT, allowing to look for highly conserved genes.

**Table 1:** Characterization of the TRAV subgroups (number of genes per functionality F and ORF and CDR-IMGT lengths) in atlantic salmon (*Salmo salarSalmo salar)* and rainbow trout *(Oncorhynchus mykiss)* arlee and swanson strains.

IMGTTRAV subgroup	*Salmo salar* ^1^	CDR-IMGT lengths	*Oncorhynchus mykiss* ^1^, Arlee	CDR-IMGT lengths	*Oncorhynchus mykiss* ^1^, Swanson	CDR-IMGT lengths
TRAV1	2F^2^	[8.6.3]	2F	[8.6.3]		
TRAV2	6F	[7.6.3]	4F	[7.6.3]	3F	[7.6.3]
TRAV3	7F	[4 (5).0.3]	11F, 1ORF	[4 (5) (8).0.3]	1F	[4.0.3]
TRAV4	2F, 2ORF^3^	[4.8.1 (2)]	5F, 1ORF	[4.7 (8).2 (3)]		
TRAV5	6F, 1ORF	[5.7.3]	9F	[5.7.2 (3)]	3ORF	[5.7.3]
TRAV6	5F, 1ORF	[4.8.2 (3)]	4F	[4.8.3]		
TRAV7	7F, 1ORF	[7 (8) (9).6.3]	3F, 1ORF	[7 (8).2 (6).3]	1F	[8.6.3]
TRAV8	4F, 1ORF	[7.10.3]	1F, 1ORF	[7.10.3]	1F	[7.10.3]
TRAV9	4F	[7.6.3]	5F	[7.6.3]	2F	[7.6.3]
TRAV10	8F, 2ORF	[7.6.3]	5F, 4ORF	[7.6.3 (10)]	2F, 3ORF	[6 (7).6.3]
TRAV11	2F	[7.6.3]	2F	[7.6.3]	1F	[7.6.3]
TRAV12	6F, 1ORF	[7 (8).6.3]	5F	[7.6.3]	2F	[7.6.3]
TRAV13	4F, 2ORF	[5.6.3]	1F	[5.6.3]		
TRAV14	4F, 1ORF	[4.3 (7).3]	5F	[4.7.3]	2F	[4.7.3]
TRAV15	2F	[5.7 (8).3]	2F, 1ORF	[5.8.3]	1F	[5.8.3]
TRAV16	3F	[6.10.3]	3F, 1ORF	[6.10.3 (4)]	2F	[6.10.3 (4)]
TRAV17	6F	[8.5.3]	5F	[8.5.3]	1F	[8.5.3]
TRAV18	6F	[7.4.2 (6)]	6F	[7.4.2 (5)]	1F	[7.4.5]
TRAV19	10F	[4.7.3]	6F, 2ORF	[4.7.3]	4F	[4.7.3]
TRAV20	3F	[6.3.3]	2F	[6.3.3]	1F	[6.3.3]
TRAV21	2F	[4.7.2]	3F	[7.4.2 (5)]		
TRAV22	1F	[7.3.11]	1ORF	[7.3.11]		
TRAV23	1F	[5.8.3]	2F	[5.8.3]		
TRAV24	1F	[5.2.3]	2F	[5.2.3]		
TRAV25	1F	(5.7.3]	1F	[5.8.3]		
TRAV26			1ORF			
Total	103F, 12 ORF		94F, 14 ORF		25F, 6ORF	

^1^TRAV genes per subgroup ^2^F, functional; ^3^ORF, Open reading frame (as defined in the text).

The structure of the tree which lacked stable branches with sequences from the three species, shows that subgroups defined in salmonids and in the zebrafish reflect completely different evolutionary pathways. This is in contrast to what is observed between different groups of mammals, with a number of TRAV subgroups conserved across primates, rodents, Cetartiodactils and carnivores (12). Understanding the evolution of TRA/DV genes would require an extensive phylogenetic work involving sequences from multiple fish species, but this simple analysis indicates that there is apparently no highly conserved TRA/DV subsets between Salmonids and Cyprinids, two basal branches of teleosts. Comparing the Atlantic salmon and Arlee TRA/TRD locus, we did not find an obvious difference in the number of members belonging to each TRAV subgroup. In Atlantic salmon, four subgroups (TRAV22 to TRAV25) are represented by a single functional gene, while TRAV10 and TRAV19 subgroups each contain 10 genes. In rainbow trout, the 25 TRAV subgroups comprise one (TRAV1, TRAV13, TRAV22, TRAV25) to twelve (TRAV3) members. The most striking differences are found in the TRAV3 subgroup, with 7 TRAV genes in Atlantic salmon and 12 TRAV genes in Arlee, and the TRAV13 subgroup, with 6 members in Atlantic salmon and 1 in Arlee (**Table 1**). In Swanson genome assembly (Omyk_1.0), we only identified TRAV genes belonging to 16 subgroups (TRAV2, 3, 5, 7 to 12 and 14 to 20). All of them could be easily matched to a unique counterpart in Arlee (*i.e.*, with a nucleotide sequence >90% similar). The distribution of TRAV genes in Arlee and Swanson was very similar, and functional genes were found in the same order. There was only one exception, the duplicated TRAV10-8D gene found in Swanson was lacking in the Arlee genome assembly (**Figure 1B** and **Supplementary Data 2**).

Multiple alignments of amino acid sequences of representative members of each TRAV subgroup are shown in **Figure 4B** and **Supplementary Data 5**. In most subgroups sequences show typical framework regions (FR-IMGT) and complementarity determining regions (CDR-IMGT) as well as canonical conserved amino acids: cysteine 23 (1stCYS) in FR1-IMGT, tryptophan 41 (CONSERVED-TRP) in FR2-IMGT, hydrophobic AA 89 (generally Leu), and cysteine 104 (2ndCYS) in FR3-IMGT (33) (**Figure 4B** and **Supplementary Data 5**).

However, TRAV sequences show structural diversity both in AA composition and in length. CDR1-IMGT and CDR2-IMGT lengths range from 4 to 9 AA and 0 to 10 AA in length, respectively, and the germline CDR3-IMGT is two to eleven AA long. The diversity is also reflected in the FR3-IMGT, most of them are 38 AA long (**Figure 4**), but there are TRAV genes with 32 (Salsal TRAV16-1), 35 (Oncmyk TRAV3-6) or 36 (Salsal and Oncmyk TRAV3-1 and TRAV3-2) AA.

### Atypical Salmonid TRAV Genes

**Figure 5:**
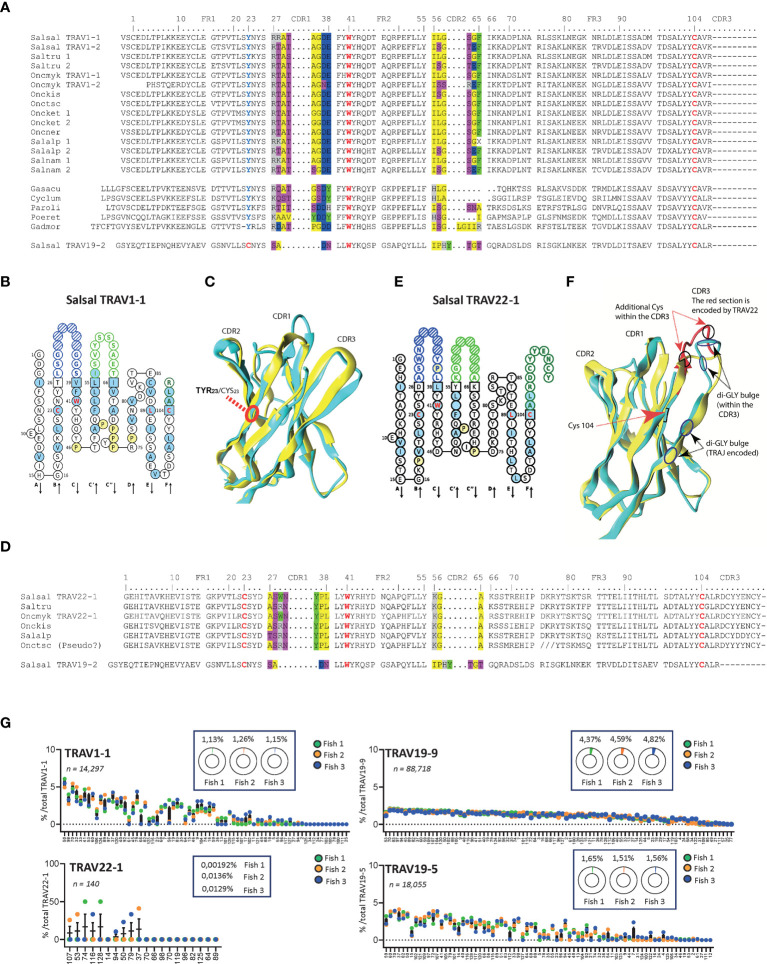
Specific features of salmonid TRAV1 and TRAV22. **(A)**. Multiple alignment of TRAV1 protein sequences from Atlantic salmon (Salsal) and rainbow trout (Oncmyk) with sequences presenting similar features from other Salmonids and other teleost. **(B)** IMGT Collier de Perles representation of Atlantic salmon TRAV1-1 based on the IMGT unique numbering for V-DOMAIN (45, 46). CDR1-IMGT is shown in dark blue and the CDR2-IMGT in light green and the germline CDR3-IMGT in dark green (33). Amino acids are shown in the one-letter abbreviation. Hatched circles correspond to missing positions according to the IMGT unique numbering (33, 46).**(C)** Salmon TRAV1 domain (cyan) aligned to human TR 2w20 alpha chain V domain. Position 23 is frame in red. **(D)** Multiple alignment of TRAV22 protein sequences from rainbow trout and Atlantic salmon with related sequences from other salmonids. **(E)** IMGT Collier de Perles of Atlantic salmon TRAV22-1. **(F)** Salmon TRAV22 domain (cyan) aligned to human TR lpha chain V domain. Note the long CDR3. The section of CDR3 encoded by TRAV22 is in red. 2nd-CYS104, additional Cys within the CDR3 loop, and Gly amino acids in di-Gly motifs (one in the G strand and one in within the CDR3) are highlighted. **(C, G)** Relative TRAJ usage shown as percentage of total reads containing a specific TRAJ gene among TRAV1-1, TRAV22, TRAV19-5 or TRAV19-9 containing productive rearrangements. Individual TRAJ genes are shown on the X-axis, average number of reads from each fish containing the indicated TRAV-TRAJ combination are shown (fish 1 is in green, fish 2 in orange and fish 5 in blue). The total number of sequences for each TRAV is indicated and the inlaid box shows the average representation of each TRAV per total number of productive rearrangements from each fish. (Atlantic salmon, *Salmo salar*: Salsal; rainbow trout, *Oncorhynchus mykiss*: Oncmyk; Brown trout, *Salmo trutta*: Saltru; Coho salmon, *Oncorhynchus kisutch*: Onckis; Chinook salmon, *Oncorhynchus tshawytscha* Onctsh; Chum salmon, *Oncorhynchus keta*: Oncket; Sockeye salmon, *Oncorhynchus nerka*: Oncner; Artic char, *Salvelinus alpinus*: Salalp; Lake trout, *Salvelinus namaycush*: Salnam; Three-spined stickleback, *Gasterosteus aculeatus*: Gasacu; Lumpfish, *Cyclopterus lumpus*: Cyclum; Olive flounder, *Paralichthys olivaceus*: Paroli; goby, *Gobiodon reticulatus*: Gobret, and Atlantic cod, *Gadus morhua*: Gadmor; guppy, *Poecilia reticulata* Poeret; sea bass, *Dicentrarchus labrax*: Diclab; barramundi perch, *Lates calcarifer*: Latcal).

**Figure 6:**
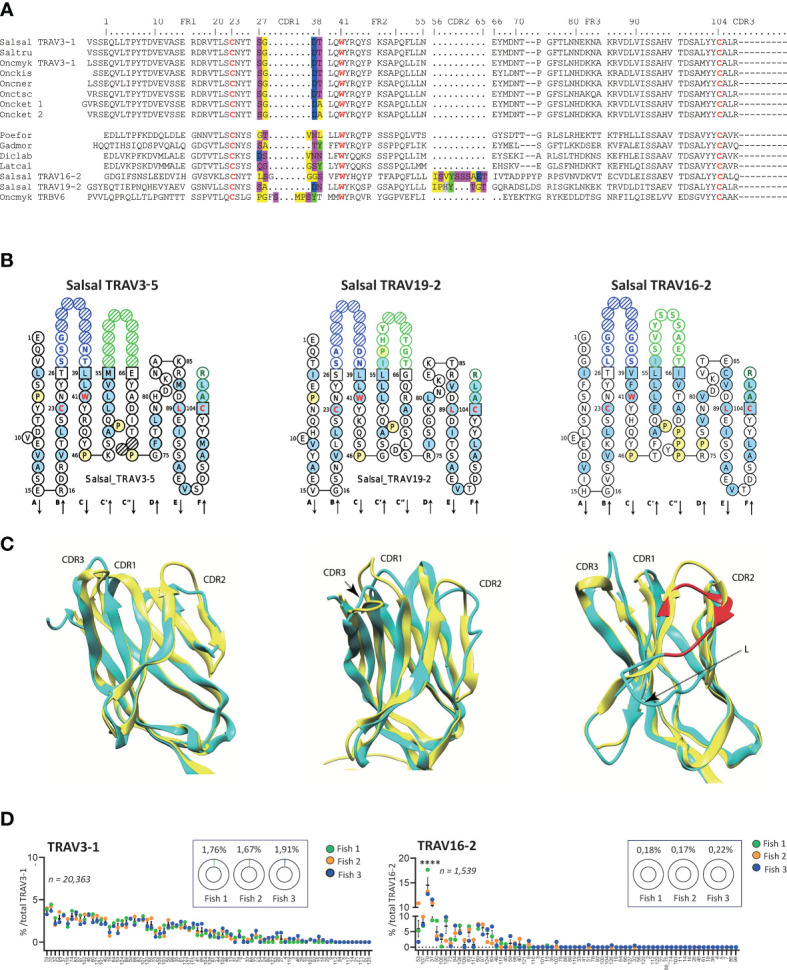
Variations of CDR2-IMGT across salmonid TRAV. **(A)** Multiple alignment of selected TRAV (TRAV22, TRAV16, TRAV19) protein sequences from Atlantic salmon (Salsal) and rainbow trout (Oncmyk) with sequences presenting similar features from other Salmonids and other teleosts. **(B)** IMGT Collier de Perles of Atlantic salmon TRAV3-5, TRAV19-2, TRAV16-2, illustrating CDR2-IMGT length variation. **(C)** Salmon TRAV3, TRAV19, and TRAV16 (cyan) aligned to human TR B7 alpha chain V domain. Note the lack of CDR2 in salmon TRAV3. Salmon TRAV19 is shown for comparison of CDR2 modelling, and TRAV16 domain for its very long CDR2 region (in red) that induces an additional loop (L) in the model, which may not reflect the reality but illustrates the constraint on the domain. **(D)** Relative TRAJ usage shown as percentage of total reads containing a specific TRAJ gene for TRAV3-1 or TRAV19-9 containing productive rearrangements. Individual TRAJ genes are shown on the X-axis, average number of reads from each fish containing the indicated TRAV-TRAJ combination are shown (fish 1 is in green, fish 2 in orange and fish 5 in blue). The total number of sequences for each TRAV is indicated and the inlaid box shows the average representation of each TRAV per total number of productive rearrangements from each fish. Atlantic salmon, Salsal; rainbow trout, Oncmyk; Brown trout, Saltru; Coho, Onckis; Chinook salmon, Onctsc; Chum salmon, Oncket; Sockeye, Oncner; African molly, Poefor; Atlantic cod, Gadmor; barramundi perch, Latcal).

Three subgroups - TRAV1, TRAV3 and TRAV22 – have unusual structural particularities as shown in **Figures 5** and **6** in comparison to a typical TRAV (TRAV19).

TRAV1 from salmon and trout consistently lack the cysteine 23 (1st-CYS), which is replaced by a tyrosine (**Figure 5A**). No other structural particularity was identified, as illustrated by the “IMGT Collier de Perles” which provides a standardized delimitation of the strands and loops of the V-DOMAIN (32) (45), or by 3D modelling based on crystallographic structures (**Figures 5B, C** **)**.

Another peculiar structural feature was found in salmon and trout TRAV22: as illustrated by the “IMGT Collier de Perles” representation, these sequences comprise a long stretch of 11 codons between the 2nd-CYS 104 and the V-HEPTAMER of the V-RS, instead of 3 or 4, as in most salmonid TRAV genes (**Figures 5D, E** **)**. Such germline-encoded CDR3 sequences might lead to particular function of TR, with possible involvement in invariant-like TR. **Figure 5F** shows that the CDR3 stretch encoded by the V gene indeed constitutes a large fraction of the loop. Remarkably, it may contain particular structural elements such as two Cys possibly involved in a S-S bridge and a di-glycine motif in the sequence presented in **Figure 5F**.

All members of TRAV3 completely lack CDR2, reminiscent of the trout TRBV6 sequences (47) (**Figure 6A**). This was observed in all twelve functional rainbow trout TRAV3 (11 in Arlee and 1 in Swanson, see **Table 1**), and the seven functional Atlantic salmon TRAV3 as well as in the other salmonids, and in species as diverse as goby, Atlantic cod, sea bass and barramundi perch. Again, this feature could not be retrieved in all teleost groups. Comparing TRAV3, TRAV19 and TRAV16 IMGT Collier de Perles and 3D models, **Figures 6B, C** illustrate the wide variation of the CDR2 length observed across salmonid TRAV domains, raising the issue of potential modes of interaction with major histocompatibility (MH) proteins.

### Salmonid TRAJ Genes

A total of 128 TRAJ genes were identified in the Atlantic salmon genome assembly. The TRAJ genes were named following the same rule proposed for human TRA/TRD locus (40), classifying the TRAJ1 as the closest TRAJ to the TRAC gene. This annotation also corresponds to the description given in Atlantic salmon by Yazawa et al. (23) (**Figures 1A** and **2A**). The nucleotide and deduced AA sequences of the 128 TRAJ genes are reported in **Supplementary Data 7**. One hundred and four TRAJ appeared to be functional genes, with canonical FGXG J-motif, without stop codons and flanked by the J-RS at the 5´ end, and a donor splice at the 3´ end. Interestingly, 15 TRAJ genes lacked the canonical FGXG motif and can be considered ORF. Nine pseudogenes were also identified in Atlantic salmon TRA locus, with the presence of stop codons. A lower number of TRAJ genes were found in rainbow trout. A total of 81 (73 F, 6 ORF, 2P) and 66 (62 F, 2 ORF and 2P) TRAJ genes were annotated in Arlee and Swanson, respectively (**Figure 2B**, **Supplementary Data 2 **and** Data 7**).

When we compared the deduced amino acid sequences of TRAJ genes from both salmonid species, we could identify at least one counterpart, except for Arlee or Swanson TRAJ9, Arlee TRAJ61 and for eight Atlantic salmon TRAJ genes (**Supplementary Data 7**). It means that TRAJ genes are conserved in both species and many TRAJ genes in Atlantic salmon are duplicated.

### Salmonid TRDJ and TRDD Genes

Only one TRDJ gene was found in salmonids (**Figures 1**, **2** and **Supplementary Data 8**). The deduced amino acid sequence is highly conserved in Atlantic salmon (DKLTFGKAINLIVEP) and rainbow trout (AKLTFGKAINLIVEP). In both species the FGKA motif replaces the hallmark motif (FGXG) identified in higher vertebrates TR J regions.

The nucleotide sequences of the three TRDD genes identified in the genome assemblies of both species are reported in **Supplementary Data 8**. They consist of 8 nt (TRDD1), 13 nt (Atlantic salmon TRDD2), 14 nt (rainbow trout TRDD2), and 13 nt (TRDD3) sequence that can be read in its three coding frames without stop codon. The 5’D-RS and 3’D-RS that flank the D-REGION are well conserved.

### Expression of Atlantic Salmon TRA Genes

**Figure 7:**
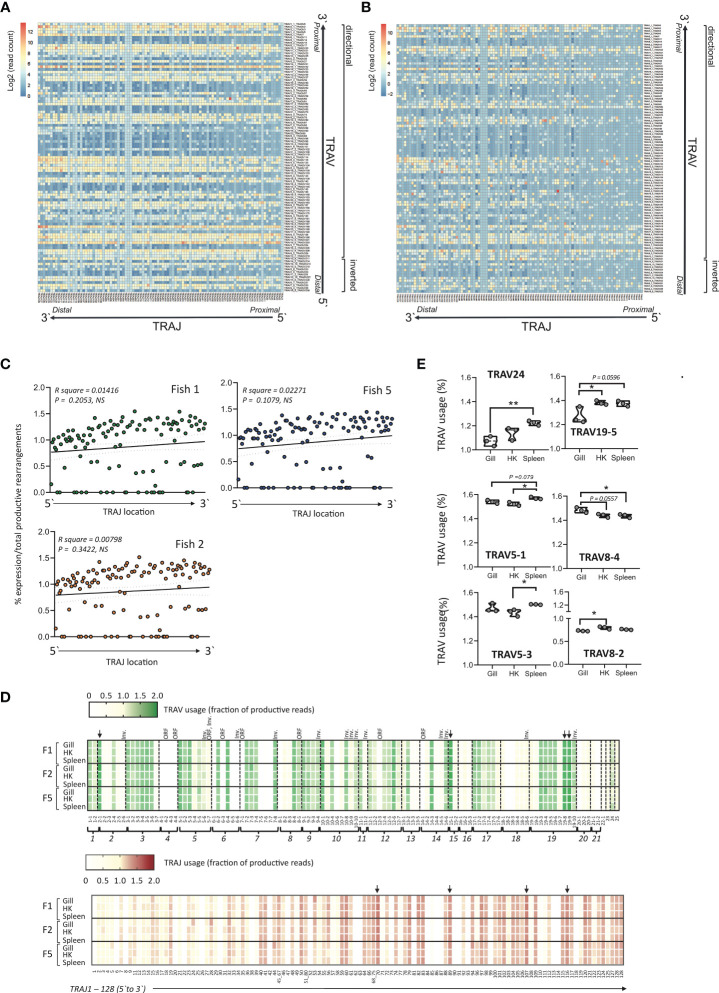
Heat map of representative productive **(A)** or unproductive **(B)** TRAV- J combinations in Atlantic salmon. The average expression of TRAV-J combinations was computed from the TRA repertoire of the total number of productive sequences from gill, head kidney and spleen from three individual presmolt Atlantic salmon. The expression of each combination was expressed as total reads and the resulting matrix was normalized and represented as a heat map with a log2 count scale. TRAJ and TRAV genes are displayed as each gene appear in the genome following a 5`to 3`direction and the transcriptional direction of the TRAV genes is indicated. **(C)** Scatterplot of the relative expression of TRAJ genes *versus* genome location showing no significant correlation. The most 5` TRAJ corresponds to TRAJ1 and is located closest to the TRAC gene. The average of normalized expression frequencies in gill, HK and from three fish is shown. **(D)** TRAV and TRAJ usage in Atlantic salmon. TRAV and TRAJ usage was determined from deep sequencing datasets of C alpha (TRAC) primed 5`-RACE generated from gill, head kidney (HK) and spleen of three unvaccinated pre-smolt Atlantic salmon. TRAV and TRAJ usage in each individual and tissue was expressed as the percentage of total productive TRA rearrangements from normalized data sets. Inv. = inverted orientation of TRAV genes, ORF = open reading frame genes (TRAV4-2, TRAV4-4, TRAV5-7, TRAV6-3, TRAV7-1, TRAV12-3 and TRAV14-1), Downward pointing arrows indicating the four most frequently used TRAV (TRAV2-1, TRAV15-1, TRAV19-8 and TRAV19-9) and TRAJ genes (TRAJ70, TRAJ89, TRAJ107 and TRAJ116) common to all three fish. **(E)** Tissue preferential TRAV usage. The relative expression of specific TRAV genes in gill, Head kidney (HK) and spleen from three pre-smolt Atlantic Salmon were determined by comparing the normalized percentage of total productive TRAV rearrangements in each tissue among the three individuals, statistical significance was determined by one way ANOVA followed by Tukey’s multiple comparisons test with *p<0.05 and **p<0.01.

The functionality and expression of TRA genes was investigated by deep sequencing of 5`-RACE products produced from spleen, head-kidney (HK) and gill of three unvaccinated presmolt Atlantic salmon. Prior to sequencing the fish were confirmed free of the salmon pathogens ISAV, SAV3, PRV and IPNV by RT-qPCR. The RACE was initiated from primers located in the TRAC gene and the total number of productive reads obtained from each sample were normalized to provide a quantitative assessment of the respective usage of TRAV, TRAJ genes and TRAV-TRAJ combinations in the different individuals and across representative tissues. Combining all datasets, the relative expression of all productive and unproductive sequences for all TRAV/TRAJ combinations are represented in **Figures 7A, B**, respectively. There was no obvious correlation between TRAV or TRAJ gene expression and the relative position in the locus. Further, TRAV genes located in either of the two TRAV blocks were used at comparable frequencies indicating that neither the transcriptional orientation nor the large gene-less region present in the locus significantly influence expression. Among the 14 inverted, functional TRAV all but one (TRAV6-6) where expressed, representing ~0.31 to 1.45% of total productive rearrangements.

To determine if these proportions reflect selective processes or rather are the result of recombination probability of TRAV genes we compared the distribution of TRAV/TRAJ among productive and unproductive transcripts. As these unproductive rearrangements are independent of TR mediated selection processes, they should reflect probabilities of rearrangements. **Figures 7A, B** show a remarkable similarity of frequency heatmap, providing a global overview of rearrangement efficiency. In general, albeit not statistically significant the TRAJ genes located on the 5’ side of the cluster (*i.e*., those closest to TRAV genes) tend to be less expressed (**Figures 7C, D** **)**. However, unlike the situation in many mammals (12, 48), where combinations of TRAV and TRAJ genes more proximal to each other tend to be favored and thus represented at a higher frequency, no clear pattern of preferential combinations could be discerned here based solely on location.

While some inter-individual variation of expression of specific TRAV-J combinations was observed the overall gene usage, both for TRAV and TRAJ, was remarkably similar among individuals and across tissues (**Figure 7D**). The four most frequently expressed V genes TRAV2-1 (1.58, 1.70 and 1.58% in fish 1, 2 and 5 respectively), TRAV15-1 (1.70, 1.69 and 1.61%), TRAV19-8 (1.64, 1.63 and 1.69%) and TRAV19-9 (1.64, 1.67 and 1.73%) were shared among all three individuals. Similarly, a highly consistent pattern in terms of genes expressed at low frequencies was apparent across individual fish, with a few TRAV genes weakly represented within the dataset, including TRAV2-6 (0.31, 0.31 and 0.32%), TRAV6-1 (0.079, 0.079 and 0.078%), TRAV7-6 (0.089, 0.088 and 0.090%), TRAV8-1 (0.124, 0.124 and 0.126%), TRAV21-2 (0.0447, 0.0449 and 0.0446%) and TRAV22 (0.447, 0.449 and 0.446). Certain V genes, including TRAV6-5, TRAV6-6, TRAV7-5, TRAV7-7, TRAV10-6, TRAV18-2, TRAV18-3 and TRAV20-1 as well as all members of the TRAV4 subgroup, which consists of two functional (TRAV4-1, TRAV4-3) and two ORF (TRAV4-2 and TRAV4-4) genes were absent from the data set. For the remaining genes annotated as ORF three (TRAV5-7, TRAV8-5 and TRAV12-3) were expressed and detected at varying degrees ranging from an average of 0.89, 1.25 and 0.18% in fish 1, 2 and 5 respectively. Overall subgroups TRAV18, which consists of six functional genes, TRAV16 (3F genes), TRAV20 (3F genes) and TRAV21 (2 F genes) were consistently underrepresented across tissues in all three fish. Highly variable TRAV frequencies within given subgroups was also revealed (**Figure 7D**). For example, TRAV2-1, which represented one of the most frequently used V genes in all three individuals represents 34% of all TRAV2 subgroup containing productive rearrangements with TRAV2-4 accounting for 24% followed by TRAV2-3 (10%), TRAV2-2 (9.9%) and TRAV2-6 (6.6%). Similar patterns with specific members of a given subgroup dominating was observed for subgroup TRAV6, TRAV7, TRAV8, TRAV10, TRAV13, TRAV14 and TRAV19. Since the V-HEPTAMER and V-NONAMER of all functional members of these TRAV subgroups are similar, and their V-SPACER has an expected length of 22 bp there is no obvious explanation for these recombination biases and may point to additional constraints related either to pairing with the beta chain, or to selection. Comparably among the TRAV3 genes, all but one gene (TRAV3-7) where expressed at a relatively uniform, moderately high frequency representing about 8.5% of the total TRAV usage in the 3 individuals. Similarly, members of the TRAV9 subgroup, which consists of four functional TRAV, are all expressed at similar levels and represented an average of about 5% of the total TRAV usage in the 3 individuals.

Similar to the pattern observed for TRAV expression, the four most frequently expressed TRAJ genes were shared among the three fish (**Figure 7D**). Most commonly used are TRAJ70 (1.54, 1.51 and 1.40% in fish 1, 2 and 5 respectively) followed by TRAJ107 (1.53, 1.45 and 1.41%), TRAJ116 (1.46, 1.41 and 1.43%) and TRAJ89 (1.43, 1.41and 1.40%). Several TRAJ genes annotated as functional were either absent from the dataset or expressed at extremely low frequencies (normalized frequency <0.05%) including TRAJ6, TRAJ8, TRAJ20, TRAJ22, TRAJ26, TRAJ30, TRAJ42, TRAJ47, TRAJ52, TRAJ62, TRAJ63, TRAJ81, TRAJ84, TRAJ86, TRAJ90, TRAJ91; TRAJ93, TRAJ113, TRAJ114 and TRAJ118.

Notably, while the majority of TRAV genes were used at similar frequency across all three tissues examined TRAV24, TRAV19-5, TRAV5-1 and TRAV5-3 predominated in the spleen, while TRAV8-3 was most common in HK and TRAV8-4 had the highest expression frequency in the gill (**Figure 7E**).

### Expression of Atypical Atlantic Salmon TRAV Genes

To gain insight into the expression profiles and potential preferential TRAJ usage of the structurally unique TRAV genes (*i.e*., TRAV1, TRAV3 and TRAV22) we determined the relative frequency of TRAV/J combinations among productive transcripts identified for each of these groups and compared this to transcripts containing two structurally conventional TRAV genes (TRAV19-5 and TRAV19-9). As an additional contrast to TRAV3, we also analyzed TRAV16 containing transcripts, which represent the three V genes with the longest CDR2 region (10 AA) in both Atlantic salmon and rainbow trout. For the representative TRAV1-1 and TRAV3-1 the overall J usage pattern was comparable to that of TRAV19. TRAV1-1 transcripts were found rearranged to 72 of the 118 functional TRAJ compared to 78 for TRAV3-1, 79 for TRAV19-5 and 90 for the highly expressed TRAV19-9. A similar pattern was obtained for the other members of the TRAV1 (TRAV1-2) and TRAV3 (TRAV3-2, TRAV3-3, TRAV3-4, TRAV3-5, TRAV3-6 and TRAV3-7) groups (**Figures 5G**, **6D**). While the individual expression profiles for TRAV1-1 and TRAV3-1 indicate some preferential J usage the overall TRAV-J combinations for both of these subgroups is diverse indicating that no strong subgroup specific selection constraints are placed on either of these TRAV groups. In contrast, sequence profiles for TRAV16-2 and TRAV22 containing transcripts were biased and displayed prominent inter-individual variation (**Figures 5G**, **6D** **)**. On average, TRAV16-2 transcripts were significantly more likely to be found rearranged to either TRAJ70 or TRAJ37 compared to any other J gene in all three individuals. However, the majority of TRAV16-2 containing productive rearrangements in the gill of fish 1 and fish 2 were combined with TRAJ53 representing 10,3 and 29,2% of total productive rearrangements in this tissue. However, in the same fish this specific combination was significantly less abundant in head kidney (1,6 and 1,01%) and spleen (4,1 and 2,31%) and overall represented less than 2% of TRAV16-2 containing rearrangements in fish 5. The remaining two TRAV16 genes, TRAV16-1 and TRAV16-3 were both expressed at very low frequencies representing less than 0,0066% of the total productive sequences and, in the majority of transcripts TRAV genes were combined with TRAJ68_or TRAJ75. Concerning TRAV22, an even more restricted profile was revealed with no TRAV22-TRAJ combinations shared between the three fish. In fish 1 all TRAV22 containing transcripts were combined with either TRAJ 37, 53 or 107, while for fish 2 TRAV22-TRAJ74 and TRAV22-TRAJ128 combinations made up 100% of the productive transcripts, and in fish 5 TRAV22 was combined with TRAJ50, 79, 94 or 116.

## Discussion

New genome assemblies from teleost species provide a high quality source of knowledge to analyze repertoire sequencing data and decipher adaptive immune responses to pathogens or vaccines. However, the full assembly of IG and TR loci remain challenging, and more efforts are required to improve their annotation and generate standardized sequence database allowing automated annotation of AIRRseq (Adaptive Immune Receptor Repertoire sequencing) data. Standardization is critical to warrant comparison across studies and species. Atlantic salmon and rainbow trout are key species for fish farming globally, and they have become important models in fish immunology. High-quality genome assemblies are now available for several salmonid species and strains. We therefore decided to revisit the description and annotation of TRA/TRD locus present in Atlantic salmon and two strains of rainbow trout (Swanson and Arlee) with the aim of establishing a common salmonid nomenclature.

The TRA/TRD gene linkage is conserved in teleosts (including salmonids) as in other vertebrates; however, the TRD genes are not embedded between the TRAV and TRAJ genes as in mammals (15, 20–23). The genomic organization of TRA/TRD locus in salmonids is similar to that described in other teleosts, with a locus in reverse orientation on the chromosome, as defined by the transcriptional orientation of the TRDC and TRAC. This TRA/TRD locus comprise at its 5’ end (chr FWD3’ side) the cluster TRDD-TRDJ-TRDC-TRAJ-TRAC followed by the translocated downstream TRAV genes at the 3’end of the locus (chr FWD5’ side)in opposite orientation (17, 20, 26). This structure implies that rearrangements of TRAV genes occur by inversion that, as opposed to deletion, would preserve the elements between the two rearranged genes. The gene rearrangement by inversion has been also described in teleost immunoglobulin light chains (49–51). This maximizes the number of genes available for secondary rearrangements, which may lead to a higher diversity and confer selective advantage through editing of self-reactive receptors. In addition, on the3´end of the Atlantic salmon TRA/TRD locus we could also identify a block of 29 TRAV genes in direct orientation as the TRDD-TRDJ-TRDC-TRAJ-TRAC cluster although downstream of it. Our results indicate that most of these TRAV are expressed in Atlantic salmon mucosal (gill) and systemic immune tissues (HK and spleen), suggesting different possibilities, or a complex rearrangement process between chromatids or a mistake in the Atlantic salmon genome assembly. Further analysis of new salmonid genomes will be helpful to solve this question and to verify if this block of TRAV genes is also present in other salmonid species.

A whole genome duplication (WGD) event occurred in the common ancestor of extant salmonids species (52–55). This WGD involved spontaneous doubling of all chromosomes (autotetraploidization), and is named “Ss4R” to account for additional rounds of WGD at the base of teleost fishes and vertebrate lineage (54). The analysis of the rainbow trout genome revealed stability of the two ancestral genome copies suggesting a slow rediploidization process (52). Two immunoglobulin heavy chain loci are present in the Atlantic salmon and rainbow trout (38, 56, 57). In contrast, there is only one TRA/TRD locus in both species, located on Atlantic salmon chromosome 14 and rainbow trout chromosome 8. The presence of two functional IGH loci significantly contributes to the increased diversity of antibodies. While antibodies recognize antigens in native form, αβ TR sense processed peptide antigens bound to MH or MH-like molecules (6). This mode of MH-restricted interaction not only imposes strong structural constraints, but also leads to highly degenerated peptide recognition by TR. This might explain at least in part the loss of one TRA/TRD locus after duplication, as the large number of TRA/TRD V, D and J genes present in one locus might be sufficient to produce a fully diversified repertoire given the number of available T lymphocytes.

Another important aspect is the impact of locus structure and/or the presence of multiple loci on the development of salmonid lymphocytes. The differentiation of T and B lymphocytes in human and mice is a highly orchestrated process, wherein the generation of productive specific antigen receptors (IG or TR), and the expression of an unique clonal receptor by each lymphocyte are key aspects (58–61). In humans and mice, early steps of αβ T lymphocyte differentiation leads to rearrangement of TRB locus, then the expression of the pre-T-cell receptor (pre-TR), which comprises a TRB chain covalently linked to the invariant pre-T alpha (pTα) chain encoded by a unrearranged gene (62, 63). Signaling through the pre-TR is involved in the induction of CD4 and CD8 expression, pre-T cell proliferation and differentiation, further TRA rearrangement and allelic exclusion (63). The allelic exclusion allows only one TRB chain to be expressed at the surface of a cell (64). By contrast, both TRA can be rearranged on both haplotypes through receptor editing (65). The potential diversity available to receptor editing is increased by the very high numbers of TRAV and TRAJ genes, a feature conserved in vertebrates and most apparent in salmonids. Interestingly, the surrogate pTα chain has been described in most mammals (human, mouse, opossum, platypus an so on), bird and lizard (66), but no pTα has been identified in teleosts and frog to date. Although a recent study with CD79-GFP transgenic zebrafish lines suggests that both IGH and IGL loci could rearrange simultaneously, without requirement of surrogate L chain (67), nothing is known about T cell maturation in teleosts. The mechanisms underlying the allelic exclusion required in salmonid TR loci to ensure clonal responses, and the possible role of the particular structure of the salmonid TRA/TRD locus implying V-(D)-J rearrangements by inversion, remain to be clarified.

While the organization of the salmonid TRA/TRD locus is conserved, the total number of TRAV genes is different in the two studied species, being 239 genes in Atlantic salmon and 163 in rainbow trout (Arlee line). Similar to what we previously described in salmonid IGH loci (38, 39), this large difference is mainly due to pseudogenes, with 123 annotated in Atlantic salmon and only 56 in rainbow trout (Arlee line). Furthermore, since there are no significant gaps (≥ 1000 bp) in the current annotated Atlantic salmon TRA/TRD locus, the higher number of TRAV genes identified by Yazawa et al. (23) maybe the result of sequence assembly from BAC clones or structural genome variations between different genetic backgrounds. Further comparisons with other de-novo genome assemblies derived from other Atlantic salmon genetic backgrounds are required to verify this hypothesis. The analysis of functional genes defined 25 subgroups shared by both salmonid species, with one single group identified only in *Oncorhynchus*, supporting a fair conservation of TRA/TRD potential repertoire across salmonids. Additionally, deep sequencing analysis data of Atlantic salmon TRA in different tissues also show that, except TRAV4, all subgroups are expressed indicating a balanced usage of the potential diversity.

Among functional salmonid TRAV genes we identified some members with nucleotide insertions and deletions that likely impact the structure of TRA. Related sequences without 1st-CYS 23, like the TRAV1 subgroup, are present in other salmonids including brown trout, Coho, Chinook, Chum, Sockeye, Artic char, and Lake trout. Interestingly, other species belonging to distinct families of teleosts also have related sequences without 1st-CYS 23, including stickleback, lump, goby, flounder and Atlantic cod. However, TRAV1 sequences could not be detected in the genome of many other fish families. No obvious evolutionary pattern could be defined, suggesting that TRAV1 like sequences might have been lost independently in multiple branches of the teleost tree.

The mode of interaction of human and murine TR with a classical pMH complex, places the germline encoded CDR1 and CDR2 loops of the TRA over the α helices of the MH protein, whereas the encoded CDR3 loops over the peptide (68–71). Thus, all members of salmonid TRAV3 subgroup that completely lack the CDR2, are unlikely to recognize pMH complexes in this mode. The lack of CDR2 loops owing to short CDR2 sequences has been also identified in trout TRBV6 (72) and in bovine TRAV genes (22). In addition, the CDR3 stretch encoded by genes belonging to salmonid TRAV22 subgroup are longer than usual and present particular structural elements that likely directly affect the antigen recognition. Analysis of expressed TRA repertoire indicates that all these particular TRAV genes are expressed at mucosal and systemic level; additionally, compared to other TRAV genes, TRAV22 showed a more restricted TRAJ usage. These structural features and restrict TRAJ suggest a possible involvement in invariant-like TR or the specialization to recognize free antigen with no antigen processing or presentation as described in other vertebrates (73, 74).

The proposed TRA/TRD locus annotation reflects the phylogenetic relationships between genes from *Oncorhynchus* (rainbow trout) and *Salmo* (Atlantic salmon). This resource will facilitate understanding diversification mechanisms, and should contribute to assess somatic hypermutation of TR sequences, a process recently reported (75). Hypermutation has been observed in dromedary (*Camelus dromedarius*) TRG/TRD (3, 76, 77), in sandbar shark (*Carcharhinus plumbeus*) TRG (78, 79), in nurse shark (*Ginglymostoma cirratum*) TRA (75, 80) and in the teleost ballan wrasse (*Labrus bergylta*) TRA (81).

In conclusion, we have provided an annotation for the complete salmonid TRA/TRD locus that represent an important step to better define the expression pattern of these TR isotypes among salmonids and through vertebrate evolution.

## Data Availability Statement

The datasets presented in this study can be found at NCBI under BioProject ID: PRJNA765183.

## Ethics Statement

The authors confirm that the experimental protocols used for the live fish experiments were based on the Animal Welfare Act (https://www.regjeringen.no/en/dokumenter/animal-welfare-act/id571188/) and performed in accordance with relevant guidelines and regulations given by the Norwegian Animal Research Authority.

## Author Contributions

E-SE, PB, and SuM conceived the project, E-SE, CF, StM, M-PL, PB, and SuM performed data analysis. E-SE, M-PL, PB, and SuM wrote the manuscript. All authors contributed to manuscript revision, read, and approved the submitted version.

## Funding

E-SE was supported by the Tromsø Research Foundation Starting Grant and by the Aquaculture program of The Research Council of Norway (Grant No. 295036). PB was supported by INRAE, by the European Commission (Grant Agreement 311993 TARGETFISH), and by the Agence Nationale de la Recherche (ANR-16-CE20-0002-01 FishRNAVax). SuM was supported by UVIGO and by Xunta de Galicia (GRC-ED431C 2020/02).

## Conflict of Interest

The authors declare that the research was conducted in the absence of any commercial or financial relationships that could be construed as a potential conflict of interest.

## Publisher’s Note

All claims expressed in this article are solely those of the authors and do not necessarily represent those of their affiliated organizations, or those of the publisher, the editors and the reviewers. Any product that may be evaluated in this article, or claim that may be made by its manufacturer, is not guaranteed or endorsed by the publisher.
