# Supplementary material for: Profiling the T Cell Receptor Alpha/Delta Locus in Salmonids
Source: Front Immunol. 2021 Oct 18;12:753960. doi: 10.3389/fimmu.2021.753960 (PMC8559430; doi:10.3389/fimmu.2021.753960)
Supplement: Supplementary file 1 [file DataSheet_1.zip › all supplementary files/Supplementary data 7.pdf]

Supplementary Data 7.

Nucleotide sequences of annotated TRAJ genes. The RS sequence is yellow highlighted.

Salmo salar (Atlantic salmon)

>Salsal_Chr14_NC_027313.1_RS_TRAJ1_F_21149566_21149664
TGGTATTACTCACAGCTAATGCACTGTGAGTGAGTGGAACAGGAATCAATAAACTTGTTTTTGGAACTG
CGGTAAAGATCACTATTGAGGCCAGTAAGT

>Salsal_Chr14_NC_027313.1_RS_TRAJ2_F_21150025_21150115
TGTTTTTGTATGAGAATAAGTTATTGTGTGAATGAGTATCAGAAGATCACATTTGGTTCAGGAACCAAA
CTTGTCATTATGACAAGTAAGT

>Salsal_Chr14_NC_027313.1_RS_TRAJ3_F_21150383_21150473
TGTTTTTGTAATGAGGCCAATGTCTGTGTGACAAGTGGATTCAAAATATCATTTGGAAATGGCATCCAA
CTAATTGTGCATTCAAGTAAGT

>Salsal_Chr14_NC_027313.1_RS_TRAJ4_F_21150841_21150934
TGTTTCTGCAACTGGACATATCATTGTGTGAATAACAACTTCAACAAAATCATTTTTGGACGTGGAACA
AAATGTGGTGTTCTGTCGAGTAAGT

>Salsal_Chr14_NC_027313.1_RS_TRAJ5_F_21151034_21151127
GGTTTATGTAATGGGGAAAAGTGTTGTGtgAATTCTGGATTTGACAAAGTAACCTTTGGGAGTGGAACA
AAATTAATAATTGATTCAAGTAAGT

>Salsal_Chr14_NC_027313.1_RS_TRAJ6_F_21152782_21152879
GCTTATTGCTCACAGTTAATGCACTGTGTTAGTGGAACAGGAATCAATAAACTTTATTTCTGGAGCAGG
AATAAAGATCACTATTGACACCAGTTTGT

>Salsal_Chr14_NC_027313.1_RS_TRAJ7_F_21153109_21153202
GGTTTTTATAACCTGACCTATCATTGTGtgAATAACAACAACTTCAAAACCATATTTGGATCTGGAACA
AAAGTTGTTGTCCTGTCACGTAAGT

>Salsal_Chr14_NC_027313.1_RS_TRAJ8_F_21153609_21153691
ATGTTTTGCAAGGGGGCATAATGTTGGATATGGCATAATAACCTTTGTCAGTGTCACAAAATTGATAAT
AGAACAAAACTTGC

>Salsal_Chr14_NC_027313.1_RS_TRAJ9_F_21154134_21154224
GGTTCTTGTAACGAGGCCATTGGCTGTGtgAATACTGGATATAAGATAATATTTGGAGTTGGAACCCAA
CTAATTATACAAGCAAGTAAGT

>Salsal_Chr14_NC_027313.1_RS_TRAJ10_P_21154354_21154449

CATTGGGTATTCACATTTTCAAATTATGGATGAATGATGGATGGAACAAAATCTACTTTCGGCAGGACA
CACAATTTATTTTTCAGCCAGGTTAAA

>Salsal_Chr14_NC_027313.1_RS_TRAJ11_F_21154511_21154604

CGTTATTGTTGAGTACTAATACAATGTGtgAATGCTAACAACCGGAAAGTCATATTTGGAGCAGGAACT
AAATTGATAATTGAAATAAGTGAGT

>Salsal_Chr14_NC_027313.1_RS_TRAJ12_F_21154999_21155092

AGTAATTGCGAAGAGATGCAACCAAGTGTGACTTATGGAGCCAACAAACTTATAATAGGACTTGGCACA
AAGATGACAGTTTATTCCAGTAAGT

>Salsal_Chr14_NC_027313.1_RS_TRAJ13_F_21155449_21155542

TGTTTCTGTTATGTCATATGTTACTGTGtgAATGATGGGACACGAAGACTAATCTTTGGAAGGGGAACCACTCTGTCAATACAATCTAGTAAGG

>Salsal_Chr14_NC_027313.1_RS_TRAJ14_F_21155746_21155842
CATTTTTGGTTAAGGCCCAATCATTGTGtgACTGACTCTGGACTGGGGAAGGTCATTTTTGGAACAGGAATTGAATTGATCATTGAAACCAGTGAGT

>Salsal_Chr14_NC_027313.1_RS_TRAJ15_F_21156082_21156175

CGTTTTTGTTGAGACAGTTTTTAGTGTGtgAATGATGGAGcaGGAAAGCTCATCTTTGGGAGTGGAACATCCCTATCTATAGAGACAAGTAAGA

>Salsal_Chr14_NC_027313.1_RS_TRAJ16_F_21156512_21156605
AGTTTTTGCAACCTGACATATCATTGTGAATACCAACAATATCAAAATCATATTTGGATTTGGTACAAAACTTGTTGTGCTGTCAAGTAAGT

>Salsal_Chr14_NC_027313.1_RS_TRAJ17_F_21157039_21157132

TGTTTTTGCAACCAGAGATATCATTGTGtgAATAGCAACTTAAACAAAATAACATTCGGATCTGGAACAAAAGTTGTCGTCTTGTCGAGTAAGT

>Salsal_Chr14_NC_027313.1_RS_TRAJ18_F_21157480_21157573

GGTTTATGCAATGGGGCAAAGTGTTGTGAATTCTGGATATGGCAAAATAACATTTGGGAGCGGCACAAAATTGATAATAGATTCAAGTAAGT

>Salsal_Chr14_NC_027313.1_RS_TRAJ19_F_21157917_21158007

TGTTTTTGTCATGAGGCCATTGGCTGTGAATACTGGAAATAAGATCATATTTGGAGTCGGAACCAAACTAATTATACAAGCAAGTAAGT

>Salsal_Chr14_NC_027313.1_RS_TRAJ20_F_21158128_21158219

TTTGGTTTTCACATTTTCAAATTATGTGAATGATATATGGAaCAAGTCTACTTTGGGCTGGGAACTTGTTCAGCCAGGTTAAAATTGTATGA

>Salsal_Chr14_NC_027313.1_RS_TRAJ21_F_21158286_21158379

AGTTATTGTTGAATACTAATTCAATGTGtgAATGTTAATAATTGGAAAGTAATATTTGGAGCAGGAACTAAATTGATAATTGAATCAAGTGAGT

>Salsal_Chr14_NC_027313.1_RS_TRAJ22_F_21158545_21158638

GCTGGCATGTAAACTTTCAAATTATGTGAACAATGGACGGAACAAAATCTACTTTGGTGCGGGCATAAAAGTTATTATTCAGCCAGGTTGTA

>Salsal_Chr14_NC_027313.1_RS_TRAJ23_F_21158704_21158797

GGTTATTGTTGAGTACTAATACAATGTGtgAATGGTAACAACCGTAAAGTAATATTTGGAGCAGGAACAAAATTGATAATTGAAACAAGTGAGT

>Salsal_Chr14_NC_027313.1_RS_TRAJ24_F_21159135_21159228

GTTTTTTTGATTGGCCATACTTACTGTGtgAATGATGGAACATGGAAGCTGATCTTTGGAGGTGGAACACAACTAAATGTAGAATCTAGTAAGA

>Salsal_Chr14_NC_027313.1_RS_TRAJ25_F_21159302_21159395

AGTAATTGCTAAGAGATGCAACATTGTGtgACTTATGAAGCCGACAAATTTATATTTGGACTTGGCACA
AAGATTACGGTTTATTCCAGTAAGT

>Salsal_Chr14_NC_027313.1_RS_TRAJ26_F_21159627_21159723

GGTTATTGGTCAGGCTTGATG*TATTGGA*TGACTGCTTTTGGAATCCAGAAAGTCCTATTTGGATCTGGA
ACTAAACTGGTCATTAAGACAAGGGAGT

>Salsal_Chr14_NC_027313.1_RS_TRAJ27_F_21159922_21160015

TGTTTCTGTTATGCCATATGTTACTGTGTGAATGATGGAACTCGAGGGTTGAACTTTGGAACTGGAACA
ACACTGTCAATACAATCTAGTAAGG

>Salsal_Chr14_NC_027313.1_RS_TRAJ28_F_21160523_21160613

TGTATTTGCTTAGATAACAAACACTGTGTgtCTGTTGGGGTCAGAGTTGTTTTTGGGCGTGGAACAAAG
TTGACGGTCAATCCAAGTAAGT

>Salsal_Chr14_NC_027313.1_RS_TRAJ29_F_21160775_ 21160868

AGTTTTTGCTCAGCCTGTTGTTAGTGTGTgtATGTTGGAGGAGAAAAGATCATCTTTGGCAGTGGAACA
TCACTGTTTGTAGAAACAAGTAAGA

>Salsal_Chr14_NC_027313.1_RS_TRAJ30_F_21161256_21161349

GGCTTATGTAATGTGGGTTCTAGTTGTGtgACTACTGATGGCCTAAAAATCATCTTTGGATCAGGCACA
AAATTAATTGTTGACTCAAGTTAGT

>Salsal_Chr14_NC_027313.1_RS_TRAJ31_F_21161474_21161564

AGTAATTGCTAAGGGATCTAGCACTGTGtgACAAGTGCGAACAAAGTCATATTCGGTCAAGGAATAAAG
CTGACCATTGACACACGTAAGT

>Salsal_Chr14_NC_027313.1_RS_TRAJ32_F_21161759_21161855

AGTTTATGTAATGTGAATTCATACTGTGtgACTACTGGAGGTGGTTACAAGATTGTCTTTGGGACAGGC
ACAAGATTAATAATTAAATCCAGTAAGT

>Salsal_Chr14_NC_027313.1_RS_TRAJ33_F_21162219_21162315

GGTTTATGTAATGTGGGTTTATGTTGTGtgACTGCTGATGGATTCAACAAAATCATCTTTGGGACCGGT
ACAAAATTAATAGTTTACTCAAGTAAGT

>Salsal_Chr14_NC_027313.1_RS_TRAJ34_F_21162399_21162482
GTTACCATTTACAATCTGTTTCACAGTGATTCAACAAAATCCTATTTGGAACAGGCACAAAATTTACCG
CTGATTTGAGTAAGT

>Salsal_Chr14_NC_027313.1_RS_TRAJ35_F_21162846_21162938

TGTTTATGTAATGTGGGTTCTTGTTGTGtgAATACGAATGGATTCAAAATCATCTTTGGGACAGGCACA
CAATTAATAGTTAACTCAAGTAAGT

>Salsal_Chr14_NC_027313.1_RS_TRAJ36_F_21163179_21163272

GGTTTATGCTATGTGGGTTCTTGATGTGtgACTCTTGGATCCTACAAAATCaTATTTGGAACAGGCACA
AAAGTAATAGTTAACTCAAGTAAGT

>Salsal_Chr14_NC_027313.1_RS_TRAJ37_F_21163895_21163988

TGTTTTTGTTAAGTCTTTAGTTAGTGTGtgAATGCTGGCGGACAAAAGCTGATCTTTGGAAGTGGAACA
ACAGTTTTTGTGGAATCAAGTAAGA

>Salsal_Chr14_NC_027313.1_RS_TRAJ38_P_21164086_21164181

GGTTTAATATAGTCAAGGAATAAAGCTGATCATTAACTCAAGTAAGTAAAAACACATTCTAAAAGGTAA
GATTAATCATAGTTGAGTCATTTAAGA

>Salsal_Chr14_NC_027313.1_RS_TRAJ39_F_21164304_21164397

AGTTTATGTACTGGAGAAAAGTATTGTGtgAATTCTgGAAATGACAAAATAACCTTTGGGAGCGGCACA
AAATGTATAATTGATTCAAGTAAGT

>Salsal_Chr14_NC_027313.1_RS_TRAJ40_F_21164800_21164890
GGTTTTTGTAATGAGGCCATTGTCTGTGtgAATGCTGGATATAAGATCATATTTGGAGTTGGAACCCAA
CTAATTGTACAAGCAAGTAAGT

>Salsal_Chr14_NC_027313.1_RS_TRAJ41_F_21165153_21165246
GGTTCTTGTTGAGTACTAATTCAATGTGtgAATGATAACAACTGGAAAGTCATATTTGGAGCAGGAACT
AAATTGATCATTGAAAGAAGTGAGT

>Salsal_Chr14_NC_027313.1_RS_TRAJ42_F_21165567_21165657

AGTCATTGCTAAGAGATGCAACACTGTGACTTGTGGAGACAAACACATATATTTGGACTTGGCACAAAG
ATGATATGTACAAAAAAGGAAT

>Salsal_Chr14_NC_027313.1_RS_TRAJ43_ORF_21165892_21165976

TTATTTGTCAGGCTTGTTGTATAGTGTGGAACCCAGAAATTCCTATTTTGATCTGGAACTAAACTATTC
ATTGAGACAAATGAAT

>Salsal_Chr14_NC_027313.1_RS_TRAJ44_F_21166361_21166460

TGTTTCTGTTATGCTATATGCTACTGTGtgAATGATGGGACGGGAACGGGAAAACTCATCTTTGGAAGG
GGAACCACACTGTCAATACAATCTAGTAAGG

>Salsal_Chr14_NC_027313.1_RS_TRAJ45_F_21166648_21166744

CGTTTTTGGTTAAGGCCCAAGCATTGTGtgACTGACTCTGGACTGGGGAAGGTCATTTTTGGAGCAGGA
ATTGAATTGGTCATTGAAACCAGTGAGT

>Salsal_Chr14_NC_027313.1_RS_TRAJ46_F_21167093_21167186
CGTTTTTGTTGAGGCAGTTTTTAGTGTGTGAATGTTGGAGCAGGAAAGCTCATCTTTGGGAGTGGAACA
TCCCTATCTATAGAGACAAGTAAGA

>Salsal_Chr14_NC_027313.1_RS_TRAJ47_F_21167316_21167407

TGCTTACTGGTAAATATCAAACATTGTGtgACTGTTGGAGTCAACAGAGTTGTTTGGGCATGGGACAAT
GTTGACGGTGAATTCATGCAAGT

>Salsal_Chr14_NC_027313.1_RS_TRAJ48_F_21167643_21167736

AGTTTTTGCTCAGCCTGTTGTTAGTGTGTgtATGCTGGAGGAGGAAAGTTCATCTTTGGCAGTGGAACA
TCACTGTTTGTAGAAACACGTAAGA

>Salsal_Chr14_NC_027313.1_RS_TRAJ49_F_21168093_21168186

GGTTTATGTAATGTGAGTTCTAGTCGTGTGATTGCTGAGGGCTTAAAAATGATCTTTGGATCAGTCACA
ACATTGATAGTCGACTCAAGTAAGT

>Salsal_Chr14_NC_027313.1_RS_TRAJ50_F_21168300_21168396

AGTTAATGTACTGTTGGTTCATGTTGTGtgACTGCTGGTGGCAATTATAAAATTATCTTTGGGAGAGGC
ACACAACTGATAGTTGACTCAAGTAAGT

>Salsal_Chr14_NC_027313.1_RS_TRAJ51_F_21168615_21168708

AGATATTGTTCAAGGCAGATGCATTGTGtgACTCAAACAAGTAGGAAAGTAATCTTTGGAACAGGGACC
AAACTAATTGTTGAAATCAGTAAGT

>Salsal_Chr14_NC_027313.1_RS_TRAJ52_F_21168910_21169005

AGTAATTAATAAGTGATCTAACATTGTGtgACAAGTGCGAACAAACTTATATTCGGTCACGTAAGTGTG
CAACATAACATTATAAAAACATCAACA

>Salsal_Chr14_NC_027313.1_RS_TRAJ53_F_21169214_21169310

AGTTTATGTACTGTGAATTCATACTGTGtgACTGCTGGAAGTGGTACCAAGATAATCTTTGGGAAAGGC
ACGAGATTAATAATTGAATCCAGTAAGT

>Salsal_Chr14_NC_027313.1_RS_TRAJ54_F_21170272_21170368

TGTTTATGTAATGTGGGTTAATGTTGTGtgACTACTGATGGATTCAACAAAATCGTCTTTGGGACAGGC
ACAAAATTGAAAGTTGACTCAAGTAAGT

>Salsal_Chr14_NC_027313.1_RS_TRAJ55_F_21170463_21170559

AGTTTATGTAATGTGAGGTAATGTTGTGtgACTAGTGGTGGACTTAACAAAATCGTCTTTGGGAAAGGG
ATCAAACTTACTGTTGCTTCGAGTAAGT

>Salsal_Chr14_NC_027313.1_RS_TRAJ56_F_21171141_21171234

GGTTTATGCTATGTGGGTTCTTGATGTGtgACTACTGGAACCGACAAAATCATCTTTGGGACAGGCACA
AAAGTAGTAGTTAACTCAAGTAAGT

>Salsal_Chr14_NC_027313.1_RS_TRAJ57_F_21171629_21171722

TGTTTTTGTTAAGTCTTTAGTTAGTGTtgGAATACTGGAACACAAAAGCTGATCTTTGGAAGTGGAACA
ACAGTTTTTGTGGAATCAAGTAAGA

>Salsal_Chr14_NC_027313.1_RS_TRAJ58_F_21171878_21171968

ATTAATTGCTATGGTATCTAGCACTGTGTgtCATATGGGAATAAATTCATACTTGGTCAAGGAACAAAG
CTGATCATTGACTCAAGTAAGT

>Salsal_Chr14_NC_027313.1_RS_TRAJ59_F_21172136_21172226

TGTTTCTGTATGGCAATACAACAGTGTGtgACTGTGGGACAAAAGTTAGTGTTTGGAAAAGGAACAATG
TTAACTGTATCAACAGGTAAGC

>Salsal_Chr14_NC_027313.1_RS_TRAJ60_F_21172436_21172535

AGTTTATGtTgTGTGGATTCATGTTGTGTGACTACTGGAGCTGGTTATGGCAAGCTTATCTTTGGGACA
GGCACCAGATTACTTATTGAATCCAGTAAGT

>Salsal_Chr14_NC_027313.1_RS_TRAJ61_F_21172985_21173075

GGTTCTTGTAATGAGGCCATTGGCTGTGtgAATACTGGATATAAGATAATATTTGGAGTTGGAACCCAA
CTTATTATACAAGCAAGTAAGT

>Salsal_Chr14_NC_027313.1_RS_TRAJ62_F_21173205_21173294

GTGGGTATTCAAACATACAAATTATGTGACTGATGGATGGAACAAAATCTACTTTGGGCATAGACTGGC
ATATTGTTCAGCCAGGTTAAA

>Salsal_Chr14_NC_027313.1_RS_TRAJ63_F_21173474_21173565

GTGGGTATTCAAACATACAAATTATGTGAATAATGGACGGAACAAAATCTACTTTGGGTTGGGCTCAAA
AGTTATTGTTCAGCCAGGTTAAA

>Salsal_Chr14_NC_027313.1_RS_TRAJ64_F_21173641_21173734

GGTTCTTGTTGAGTAATAATTCAATGTGtgAATGATAACAACCAGAAAGTCATATTTGGAGAAGGAAGT
AAATTGATAATTGAAACAAGTGAGT

>Salsal_Chr14_NC_027313.1_RS_TRAJ65_P_21173972_21174069
ATTGTTATTGGTCACACAATATCATTGTGTGACCGCATCGGGAATCCAGAATGTCCCACTTGGATCTCG
AACTAAACTATTAATTGAGACACGTTCAT

>Salsal_Chr14_NC_027313.1_RS_TRAJ66_F_21174245_21174338
TGTTTCTGTAATGTCATTTATTACTGTGtgAATGATGGGACTCAAAGGCTAATCTTTGGAAGTGGAACA
ACACTGTCAATACAATCTAGTAAGG

>Salsal_Chr14_NC_027313.1_RS_TRAJ67_F_21174527_21174623
TGTTTTTGGTTAAGGCCCAAGCAATGTGtgACTGACTCTGGACTGGGGAAGGTCATTTTTGGAGCAGGA
ATTGAATTGGTCATTGAAACCAGTGAGT

>Salsal_Chr14_NC_027313.1_RS_TRAJ68_F_21174868_21174961
CGTTTTTGTTGAGGCAGTTTTTAGTGTGtgAATGATGGAGCACGAAAGCTCATCTTTGGGAGTGGAACA
TCCTTATCTATAGAGACAAGTAAGA

>Salsal_Chr14_NC_027313.1_RS_TRAJ69_P_21175135_21175221
GGTTcATGTAATGTGAGTTGTAGTTGTGACTGATGGCCTAAAAATAGTGTTTGGATCAGGTACAAAATG
AATTGTTGACTCAAGTAAGT

>Salsal_Chr14_NC_027313.1_RS_TRAJ70_F_21175308_21175404
AGTTAATGTAATGTAGGTTCATGTTGTGtgACTACTGGTGGGCTCAGTAAAATGATCTTTGGGGCAGGC
ACACAATTAATAGTTGACTCAAGTAAGT

>Salsal_Chr14_NC_027313.1_RS_TRAJ71_F_21180093_21180182
TGTTTTTGTTAAGTCTTTAGTGTGTGAATACTGCAACACAAAAGCTGATCTTTGGAAGTGGAACAACAG
TTTTTGTGGAATCAAGTAAGA

>Salsal_Chr14_NC_027313.1_RS_TRAJ72_F_21180304_21180394

AGTAATTGCTAGTGTATCTAACACTGTGTGTCATATGGGAATAAATTCATACTTGGTCAAGGAATAAAG
CTGATCATTGACTCTAGTAAGT

>Salsal_Chr14_NC_027313.1_RS_TRAJ73_F_21180569_21180660

CTGTAGGACATGGATATATAACAGTGTGTGACTGTGGGACAAAAGTTAGTGTTTGGTAAAAGGAACAAT
GTTAACTGTATCAACAGGTAAGC

>Salsal_Chr14_NC_027313.1_RS_TRAJ74_F_21180862_21180961

AGTTTATGTTGTGTGGATTCATGTTGTGTGACTTCTGGAGCTGGTTCTGACAAGCTTATCTTTGGGACA
GGCACCAGATTACTTATTGAATCCAGTAAGT

>Salsal_Chr14_NC_027313.1_RS_TRAJ75_F_21182353_21182446

CGTTTTTGTTGAGACAGTTTTTAGTGTGTGAATGATGGAGCACGAAAGCTCATCTTTGGGAGTGGAACA
TCCTTATCTATAGAGACAAGTAAGA

>Salsal_Chr14_NC_027313.1_RS_TRAJ76_P_21182620_21182706

GGTTCATGTAATGTGAGTTGTAGTTGTGACTGATGGCCTAAAAATAGTGTTTGGATCAGGTACAAAATG
AATTGTTGACTCAAGTAA

>Salsal_Chr14_NC_027313.1_RS_TRAJ77_F_21182794_21182890

AGTTAATGTAATGTAGGTTCATGTTGTGTGACTACTGGTGGGCTCAGTAAAATGATCTTTGGGGCAGGC
ACACAATTAATAGTTGACTCAAGTAAGT

>Salsal_Chr14_NC_027313.1_RS_TRAJ78_F_21183094_21183187

GGTTTATGTAATGTGGGTTCTAGTTGTGtgACTACTGATGGCCGAAAAATCCTCTTTGGATCAGGCACA
AAATTAATAGTCGACTCAAGTAAGT

>Salsal_Chr14_NC_027313.1_RS_TRAJ79_F_21183312_21183408

AGTTAATGTACTGTGGGTTCATGTTGTGtgACTACTGGTAGCAATGTTAAAATCATCTTTGGGAGAGGC
ACACAACTGATAGTTGACTCAAGTAAGT

>Salsal_Chr14_NC_027313.1_RS_TRAJ80_F_21183626_21183719

AGATATTGTTCAAGGCAGATGCATTGTGTGACTCAAACAAGTAGGAAAGTAATCTTTGGAACAGGGACC
AAACTAATTGTTGAAATCAGTAAGT

>Salsal_Chr14_NC_027313.1_RS_TRAJ81_F_21183929_21184019

GTAATTAATAAGTGATCTAACATTGTGTGACAAGTGCGAACAAACTTATATTCGGTCAAGGAATAAAGG
TGACTATTGACTCACGTAAGT

>Salsal_Chr14_NC_027313.1_RS_TRAJ82_F_21184231_21184327

AGTTTATGTAATGTGAATTCATACTGTGtgACTGTTGGAGGTGGTTCCAAGATTATCTTTGGGACAGGC
ACAAGATTAATACTCAAATCCAGTAAGT

>Salsal_Chr14_NC_027313.1_RS_TRAJ83_F_21185172_21185265

GGTTTATGCTATGTGGGTTCTTGATGTGtgACTGGTGCAGCCAACAAAATCATCTTTGGGACAGGCACA
AAAGTAATAGTTAACTCAAGTAAGT

>Salsal_Chr14_NC_027313.1_RS_TRAJ84_F_21185667_21185760

TGTTTTTGTTAAGTCTTTAGTTAGTGTGtgAATACTGGCGGACAAAAGCTGATCTTTGGAAGTGGAACA
ACAGTTTTTGTGGAATCAAGTAAGA

>Salsal_Chr14_NC_027313.1_RS_TRAJ85_F_21185916_21186006

AGCAATTGCTATGGTATCTAACACTGTGTgtAATACGGGAATAAATTCATACTTGGTCAAGGAACAAAG
CTGATCATTGACTCAAGTAAGT

>Salsal_Chr14_NC_027313.1_RS_TRAJ86_F_21186196_21186286

TGTTTCTGTATGGCAATACAACAGTGTGTGACTGTGGGACAAAAGTTAGTGTTTGGAAAAGGAACAATG
TTAACTGTATCAACAGGTAAGC

>Salsal_Chr14_NC_027313.1_RS_TRAJ87_F_21186494_21186593

AGTTTATGTTGTGTGGATTCATGTTGTGTGACTTCTGGAGCTGGTTATGGCAAGCTTATCTTTGGGACA
GGCACCAGATTACTTATTGAATCCAGTAAGT

>Salsal_Chr14_NC_027313.1_RS_TRAJ88_F_21187043_21187133

TGTTTTTGTCATGAGGCCATTGGCTGTGtgAATACTGGAAATAAACTAACATTTGGAGTTGGAACCCAA
CTAATTATACAATCAAGTAAGT

>Salsal_Chr14_NC_027313.1_RS_TRAJ89_F_21187433_21187526

GGTTATTGTTGAGGTCTAATTCAATGTGtgATTACTAACAACCAGAAAGTCATATTTGGAGCAGGAACT
AAATTGGTAATTGAAACAAGTGAGT

>Salsal_Chr14_NC_027313.1_RS_TRAJ90_F_21187921_21188007

CGTTTTTTGATCGGCCATGTGtgAATGATGGAACATGGAATCTGATCTTTGGAAGTGGAACACAACTAT
TTATAGAATCTAGTAAGA

>Salsal_Chr14_NC_027313.1_RS_TRAJ91_F_21188067_21188158

AGTAATTGCTAAGATATGCAACAATGTGtgACTTATGGAGACAGCAAAGTTATATTTGGACTTGGCACA
AAGATGTTTTTTTCCCAGTAAGT

>Salsal_Chr14_NC_027313.1_RS_TRAJ92_F_21188371_21188467

GCTTATTGGTCAGGCTTGATGTATTGTGtgACTGCATCTGGGATCCAGAAAGTCCTATTTGGATCTGGA
ACTAAACTATTCATTGAGACAAGTGAGT

>Salsal_Chr14_NC_027313.1_RS_TRAJ93_F_21188638_21188725

CCAGTGTTTCTGTTATGTCGAATGAATGTTGGGACACGAAGACTGATCTTTGGCAGTGGAACGACACTG
TCAATACAATCTACTAAGG

>Salsal_Chr14_NC_027313.1_RS_TRAJ94_F_21188913_21189009

ACTTTTTGGTTAAGGCCCAATCATTGTGtgACTGACTCTGGACTGAAGAAGGTCATTTTTGGAACAGGA
ATTGAATTGATCATTGAAACCAGTGAGT

>Salsal_Chr14_NC_027313.1_RS_TRAJ95_P_21189350_21189438

GGTTcATGTAATGTGAGTTGTAGTTGTGACTGATGGCCTAAAAATAATGTTTTGATCAGGTACAAAATT
AATTGTTGACTCAAGTAAGT

>Salsal_Chr14_NC_027313.1_RS_TRAJ96_F_21189528_21189627

AGTTTATGTGCTGTTGGTTCATGTTGTGtgACTGCTGGTGGTGGCAACAGTAAAATGATCTTTGGGGCA
GGCACGCAATTAATAGTTGACTCAAGTAAGT

>Salsal_Chr14_NC_027313.1_RS_TRAJ97_F_21189831_21189924

GGTTTATGTAATGTGGGTTCTAGTTGTGtgACTACTGCTGGCCGAAAAATCCTCTTTGGATCAGGCACA
AAATTAATAGTCGACTCAAGTAAGT

>Salsal_Chr14_NC_027313.1_RS_TRAJ98_F_21190049_21190148

AGTTTATGTAATGTAGGTTCGTGTTGTGtgACTACTGGTGGTGGCCTCGATAAAATCATCTTTGGGACA
GGCACAAAATTAATAGTTGACTCAAGTAAGT

>Salsal_Chr14_NC_027313.1_RS_TRAJ99_P_21190351_21190444

AGATATTGTTCAAGGCAGATGCATTGTGtgACTTAAACAAGCAGAAAAGTAATCTTCGGAACAGGGACC
AAACTAGTTATTGAAACCAGTAAGT

>Salsal_Chr14_NC_027313.1_RS_TRAJ100_F_21190736_21190826

AGAAATTGTTAAGGTGTCAAGCACTGTGtgACAAGTGGGAGCAAAGTCATATTCGGTCAAGGAACAAAG
CTGACCATTGACTCACGTGAGT

>Salsal_Chr14_NC_027313.1_RS_TRAJ101_F_21191019_21191115

AGTTTATGTCATGTGGAATCATGCTGTGtgACTACTGGAGTTGGTGACAAGATTATCTTTGGGACAGGC
ACAAGATTAATAATTCAATCCAGTAAGT

>Salsal_Chr14_NC_027313.1_RS_TRAJ102_F_21191427_21191523
GGTTTATGTAATGTGGGTTCATGTTGTGtgACTACTGCTGGAGTCAACAAAGTCATCTTTGGGACCGGC
ACAAAATTAATAATTGACTCAAGTAAGT

>Salsal_Chr14_NC_027313.1_RS_TRAJ103_F_21191628_21191724

GTTTTATGTATTGTGAGTTCATGTTGTGtgACTAGTGGTGGATTCAACCAAATCATCTTTGGAACAGGC
ACAAAACGAATAGTTTATTCGAGTAAGT

>Salsal_Chr14_NC_027313.1_RS_TRAJ104_F_21192416_21192515
AGTTTATGTGCTGTGGGTTCATGTTGTGtgACTACTGGTGTTGGCACCGGTAAAATGATCTTTGGGACA
GGCACGCAATTAACAGTTGACTCAAGTAAGT

>Salsal_Chr14_NC_027313.1_RS_TRAJ105_F_21192717_21192810
AGATATTGTTCAAGGCAGATGCATTGTGtgACTCTGTCAAGCAGTAAAGTCATTTTTGGAACAGGGACC
AAACTAATTATTGAAACCAGTAAGT

>Salsal_Chr14_NC_027313.1_RS_TRAJ106_F_21193082_21193172
AGTAACTGCTGAGGGATCTAGCACTGTGtgACAAGTGCAAACAAAGTCATATTCGGTCAAGGAATAAAG
TTGACTATTGACTCACGTAAGT

>Salsal_Chr14_NC_027313.1_RS_TRAJ107_F_21193381_21193477
AGTTTATGTCATGTGGATACATACTGTGtgACTACTGGAGGTGGTAACAAGATTGTCTTTGGGACAGGC
ACAAGATTAATAATTGAATCCAGTAAGT

>Salsal_Chr14_NC_027313.1_RS_TRAJ108_F_21193843_21193933
GGTTTATGTAATGTGGGTTCGTGTTGTGtgACTACTGTCAGCAAAATCATCTTTGGGACAGGCACAAAA
TTAAAAGTTGACTCAAGTAAGT

>Salsal_Chr14_NC_027313.1_RS_TRAJ109_F_21194206_21194296

AGTAATTGCTAAGGAATATAGCACTGTGtgACAAGCACGAACAAAGTCATATTTGGTCAAGGAATAAAG
CTGACTATTAACTCACGTAAGT

>Salsal_Chr14_NC_027313.1_RS_TRAJ110_P_21194416_21194508

GGTTTATGTAATGTGAGTTCTCGTTGTGtgAATACGGGTGGATTCAAAATCATCTTTGGGACAGGCTCA
AATTAATAGTGAACTCAAGTAAGT

>Salsal_Chr14_NC_027313.1_RS_TRAJ111_F_21194734_21194826
GTTTATGCTATGTGGGTTCTTGATGTGTgACTCAAAGAGCCAACAAAATCATCTTTGGGACAGGCACAA
AAGTAATAGTTAACTCAAGTAAGT

>Salsal_Chr14_NC_027313.1_RS_TRAJ112_F_21195221_21195314

TGTTTTTGTTAAGTCTTTAGTTAGTGTGtgAATACTGCAACACAAAAGCTGATCTTTGGAAGTGGAACA
AGAGTTTTTGTGGAATCAAGTAAGA

>Salsal_Chr14_NC_027313.1_RS_TRAJ113_F_21195467_21195556

ACAAAATGCTATGGTTATTAACACTGTGTAAATACGGGAATAAATTCATACTTGGTCAAGGAACAAAGC
TGATCATTCACTCAAGTAAGT

>Salsal_Chr14_NC_027313.1_RS_TRAJ114_F_21195756_21195847

*CTACGGTAG*GACATGGATATA*TAAGTGT*GTGACTGTGGGACAAAAATTAGTGTTTGGAAAAGGATTAAA
GTTAACTGTATCAACAGGTAAGC

>Salsal_Chr14_NC_027313.1_RS_TRAJ115_F_21196029_21196128

AGTTTATGTTGTGTGGATTCATGTTGTGTGACTTCTGGAGCTGGTTCTGGCAAGCTTATCTTTGGGACA
GGCACCAGATTACTTATTGAATCCAGTAAGT

>Salsal_Chr14_NC_027313.1_RS_TRAJ116_F_21196695_21196788
TGTAAATGTATTGCAATCTACCACTGTGTgtCTAACGCTGGAGGGAAGCTTACATTTGGTACAGGAACA
AAGCTAATAATTGAGACAAGTAAGA

>Salsal_Chr14_NC_027313.1_RS_TRAJ117_F_21196889_21196985

AATCAGGTAAATAAAACAAAATACTGTGtgACAAGGGGGAATAGTAACAGGATTATCTTTGGATATGGA
ACCCAAATGTTAGTTGAATCAGGTAAAT

>Salsal_Chr14_NC_027313.1_RS_TRAJ118_F_21196974_21197070
AATCTGGTAAGTAAAACAAAATACTGTGtgACAAGCGGGAATAGTAACGGGATTATCTTTGGAAATGGA
ACCCAAATGTTAGTTGAATCAGGTAAAT

>Salsal_Chr14_NC_027313.1_RS_TRAJ119_F_21197059_21197155
GGTTTTTAGCTGGTAACAAAACACTGTGtgACAAGGGGGAATAGTAACAGGATTATCTTTGGATATGGA
ACCCAATTGTTAGTTGAATCTGGTAAGT

>Salsal_Chr14_NC_027313.1_RS_TRAJ120_F_21197273_21197367
TATTTGGTAAGGTGTGTATCTCAGTGTGTCTGCCCAGGGGGGATACAAGCTTATTTTTGGAAGTGGAAC
TAAAGTGATTGTAGAGACACGTAAGT

>Salsal_Chr14_NC_027313.1_RS_TRAJ121_F_21197778_21197871
AGTTTATATACTGGTGTGTTAGGCTGTGtgAATGATGCCGGTACAAAGATCATTTTTGGGAGAGGCACA
AGGCTACTAGTATTAAGCAGTAAGT

>Salsal_Chr14_NC_027313.1_RS_TRAJ122_F_21197999_21198095
AGTAATTGTACTGCAGTTTAATGCTGTGtgAATACTGGGTCTGCAGGAAAACTTGTGTTTGGTAGTGGC
GCCAAACTGTTCATAAATACTGGTAGGT

>Salsal_Chr14_NC_027313.1_RS_TRAJ123_F_21198354_21198447
GGTTTTTAGTGAGCAAGAATACACTGTGtgACAAGAACAAGTGACAAAATTACTTTTGCACGTGGGACT
CAACTGTTTGTAGAGAAAGGTGAGA

>Salsal_Chr14_NC_027313.1_RS_TRAJ124_F_21198691_21198785
TAATCGTCATGGTCTGTATCTCAGTGTGAATGCCCAGGGGGGAGTAAAGCTTATTTTTGGGAGTGGAAC
AAAAATGATTGTGGAGACACGTAAGT

>Salsal_Chr14_NC_027313.1_RS_TRAJ125_F_21199378_21199474
TGTAATTGTACTGCAGTTTAACACTGTGtgACTACTGGAACAAACGGAAAATTcATATTTGGTAGCGGA
ACCAAACTGTTCATAAATACTGGTAGGT

>Salsal_Chr14_NC_027313.1_RS_TRAJ126_P_21199721_21199812
GGTTTTTAGTGAGAAAAAAATACGCTGAGtgACGACAAGTGAAAAAATGTATTTTGCACGTGGAACTCA
ACTGTTTGTAGAGTAAGGTGAGA

>Salsal_Chr14_NC_027313.1_RS_TRAJ127_F_21200051_21200147
AGTAATTGTCATGGTCTGTATCTCAGTGAATGCTCAGGGAGCAGGAAAGATATATTTTGGGAATGGAAT
CAAAATGATTGTGGGGACACGTAAGT

>Salsal_Chr14_NC_027313.1_RS_TRAJ128_F_21200355_21200450
GATTTGTGTACAGGAGTGTAGCACTGTGGTTGGCTGGCTCTAACAATAAGCTGATTTTTGGATCTGGAG
TGAACGTCATTGTGCAATCCCGTAAGT

***Oncorhynchus mykiss* (Arlee strain)**

>aOncmyk_Chr8_NC_048572_RS_TRAJ1_F_61944197_61944292

TGGTATTGCTCACAGCTAATGAACTGTGAGTGACTGGAACAGGAATCAATAAACTTGTTTTTGGAACTG
CGGTAAAGATCACTATTGAGGCCAG

>aOncmyk_Chr8_NC_048572_RS_TRAJ2_F_61944644_61944735

TGTTTTTGCATGAGAATAAGTTATTGTGTGAATGAGTATCAGAAGATCACATTTGGTTCAGGAACCAAA
ATGTTCATTATGACAAGTAAG

>aOncmyk_Chr8_NC_048572_RS_TRAJ3_F_61945002_61945089

TGTTTTTGTAATGAGGCCAATGTCTGTGTGACAAGTGGATTCAAAATATCATTTGGAAATGGCATCCAA
CTAATTGTGCATTCAAG

>aOncmyk_Chr8_NC_048572_RS_TRAJ4_F_61948527_61948614

GGTTCTTGTAATGAGGCCTTTGTCTGTGTGAATACCGGATATAAGATAACATTTGGAGTTGGAACCCAA
CTAATTATACAAGCAAG

>aOncmyk_Chr8_NC_048572_RS_TRAJ5_F_61948908_61948998

CGTTATTGCTGAGTACTAATACAATGTGTGAATGCTAACAACCGGAAAGTCATATTTGGAGTAGGAACT
AAATTGATAATTGAAATAAG

>aOncmyk_Chr8_NC_048572_RS_TRAJ6_F_61949397_61949487

AGTAATTGCTAAGAGATGCAACACAGTGTGACTTACGGAGCCAACAAACTTATATTTGGACTTGGCACA
AAGATGACAGTTTATTCCAG

>aOncmyk_Chr8_NC_048572_RS_TRAJ7_F_61952754_61952842

TTTATGCAATGGGGCAAAGTGTTGTGTGAATTCTGGATATGGCAAAATAACATTTGGAGCGGCACAAA
ATTGATAATAGATTCAAG

>aOncmyk_Chr8_NC_048572_RS_TRAJ8_F_61953191_61953279

GGTTTTTGTAATGAGGCCATTGCCTGTGTGAATACTGGAAATAAGATCATATTTGGAGTCGGAACCCAA
CTAATTATACAAGCAAGT

>aOncmyk_Chr8_NC_048572_RS_TRAJ9_F_61953432_61953511

TTTAGTTTTCACATTTTCAAATTATGTGAATGATATATGGAACAAAGTCTACTTTGGGCTGGGAACTTG
TTCAGCCAGGT

>aOncmyk_Chr8_NC_048572_RS_TRAJ10_F_61953581_61953671

AGTTATTGTTGAATACTAATTCAATGTGTGAATGTTAATAACTGGAAAGTAATACTTGGAGCAGGAACT
AAATTGATAATTGAATCAAG

>aOncmyk_Chr8_NC_048572_RS_TRAJ11_F_61953841_61953929

AGCTGGCATGTAAACTTTCAAATGATGTGAACAATGGATGGAATAACATCTACTTTGGTGAGGGCATAA
AAGTTATTATTCAGCCAG

>aOncmyk_Chr8_NC_048572_RS_TRAJ12_F_61953999_61954089

GGTTATTGTTGAGTACTAATACAATGTGTGAATGGTAACAACCGGAAAGTCATATTTGGAGCAGGAACA
AAATTGATAATTGAAACAAG

>aOncmyk_Chr8_NC_048572_RS_TRAJ13_F_61954436_61954526

GTTTTTTTGATTGGCCATACTTACTGTGTGAATGATGGAACGTGGAAGCTGATCTTTGGAGGTGGAACA
CATCTAAATGTAGAATCTAG

>aOncmyk_Chr8_NC_048572_RS_TRAJ14_P_61955231_61955325

TGTTTCTGTTATGCCATATGTTCCTGTGTGAATGATGGAACTCGAGGATTGAACTTTGGAACTGGAACA
ACACTGTCAATATAATCTAGTAAG

>aOncmyk_Chr8_NC_048572_RS_TRAJ15_F_61955724_61955811

TGTATTTGCTTAGATAACAAACACTGTGTGACTGTTGGGGTCAGAGTTGTTTTTGGGCATGGAACAAAG
TTGACGGTCAATCCAAG

>aOncmyk_Chr8_NC_048572_RS_TRAJ16_F_61956441_61956530

CTTATGTAATGTGGGTTCTAGTTGTGTGGCTACTGATGGCCTAAAAATCGTCTTTGGATCAGGCACAAA
ATTAATTGTTGACTCAAGTTA

>aOncmyk_Chr8_NC_048572_RS_TRAJ17_F_61956656_61956747

AGTAATTGCTAAGATGTCTAGCACAGTGTGACAAGTGCGAACAAACTCATATTCGGTCAAGGAATAAAG
CTGACTGTTGACACACGTAAG

>aOncmyk_Chr8_NC_048572_RS_TRAJ18_F_61956936_61957029

AGTTTATGTAATGCGAATGCATACTGTGTGACTACTGGAGGTGGTTACAAGATTGTCTTTGGGACAGGC
ACAAGATTAATAATTAAATCCAG

>aOncmyk_Chr8_NC_048572_RS_TRAJ19_F_61957366_61957459

GGTTTATGTAATGTAGGTTCATGTTGTGTGACTGCTGATGGATTCAACAAAATCATCTTTGGGACAGGC
ACAAAATTAATAGTTTACTCAAG

>aOncmyk_Chr8_NC_048572_RS_TRAJ20_F_61958004_61958098

GGTTTATGTTGTGTCGGTTCTTGTTGTGTGAATGCGAATGGATTCAAAATCATCTTTGGGACAGGCACA
ACATTAATAGTTAACTCAAGTAAG

>aOncmyk_Chr8_NC_048572_RS_TRAJ21_F_61958962_61959056

TGTTTTTGTTAAGCCTTTAGTTAGTGTGTGAATATTGGCGGACAAAAGCTGATCTTTGGAAGTGGAACA
ACAGTTTTTGTGGAATCAAGTAAG

>aOncmyk_Chr8_NC_048572_RS_TRAJ22_F_61960186_61960276

GGTTCTTGTTGAGTACTAATTCAATGTGTGAATGATAACAACTGGAAAGTCATATTTGGAGCAGGAACT
AAATTGATCATCGAAAAAAG

>aOncmyk_Chr8_NC_048572_RS_TRAJ23_F_61961383_61961477

TGTTTATGTTATGTCATATGTTACTGTGTGAATGATGGGACAGGAAGACTGATCTTTGGAAGGGGAACC
ACACTGTCAATACAATCTAGTAAG

>aOncmyk_Chr8_NC_048572_RS_TRAJ24_F_61961661_61961758

CATTTATAGTTAAGCCCCAATCATTGTGTGACTAACTCAGGACTGGGGAAGGTCATTTTTGGAGCAGGA
ATTGAATTGATCATTGAAACCAGTGAG

>aOncmyk_Chr8_NC_048572_RS_TRAJ25_F_61962116_61962206

CATTTTTGTTGAGCCTGTTTTTAGTGTGTGAATGATGGAGTGAGAAAGCTCATCTTTGGGAGTGGAACA
TCACTATCTGTAGAGACAAG

>aOncmyk_Chr8_NC_048572_RS_TRAJ26_F_61962351_61962444

TGCTTACTGGTAAATATCAAACATTGTGTGACTTTGGAGTCAACAGAGTTGTGTTTGGGCATGGGACAA
TGTTGACGGTCAATTCATGCAAG

>aOncmyk_Chr8_NC_048572_RS_TRAJ27_F_61962673_61962763

AGTTCTTGCTCAGCCTGTTTTTAGTGTGTGTATGCTGGAGGAGGAAAGTTCATCTTTGGCAGTGGAACA
TCACTGTTTGTAAAAACAAG

>aOncmyk_Chr8_NC_048572_RS_TRAJ28_F_61963344_61963437

AGTTAATGTACTGTGGGTTCATGTTGTGTGACTACTGGTGGCAATGTTAAACTTATCTTTGGGAGAGGC
ACACAATTAATAGTTGACTCAAG

>aOncmyk_Chr8_NC_048572_RS_TRAJ29_F_61963613_61963703

AGATATTGTTCAAGGCAGATGCATTGTGTGACTCAAACAAGTAGGAAAGTCATCTTTGGAACAGGGACC
AAACTAGTTATTGAAACCAG

>aOncmyk_Chr8_NC_048572_RS_TRAJ30_ORF_61963934_61964026

AGTAATTGATAAGTGATCTAACACTGTGTGACAAGTGCGAAAAAAACGTATATTCGGTCAAGGAATAAA
GGTGACTATTGACTCACGTAAG

>aOncmyk_Chr8_NC_048572_RS_TRAJ31_F_61964234_61964327

AGTTTATGTAATGTGAATTCATACTGTGTGACCGTTGGAGGTGGTTCCAAGATTACCTTTGGGACAGGC
ACAAGATTAATACTCAAATCCAG

>aOncmyk_Chr8_NC_048572_RS_TRAJ32_F_61965191_61965281

GGTTTATGCTATGTGGGTTCTTGATGTGTGATTGGTGGAGCCAACAAAATCATCTTTGGGACAGGCACA
AAAGTAATAGTTAACTCAAG

>aOncmyk_Chr8_NC_048572_RS_TRAJ33_F_61965686_61965776

TGTTTCTGTTAAGCCTTTAGTTAGTGTGTGAATGCTGGCGGTCAAAAGCTGATCTTTGGAAGTGGAACA
ACAGTTTTTGTGCAATCAAG

>aOncmyk_Chr8_NC_048572_RS_TRAJ34_F_61965926_61966017

ATTAATTGCTGGGGTATCTAACACTGTGTGTCATATGGGAATACATTCATACTTGGTCAAGGAACAAAG
CTGATCATTGACTCAAGTAAG

>aOncmyk_Chr8_NC_048572_RS_TRAJ35_F_61966483_61966579

AGTTTATGTTGTGTGGATTCATGTTGTGTGACAACTGGAGTTGGTTCTAGCAAGCTTATCTTTGGGACA
GGCACCAGATTACTTATTGAATCCAG

>aOncmyk_Chr8_NC_048572_RS_TRAJ36_F_61967018_61967103

TTCTTGTAATGAGGCCATTGGCTGTGTGAATACTGGATATAAGATAACATTTGGAGTTGGAACCCAACT
AATTGTACAAGCAAG

>aOncmyk_Chr8_NC_048572_RS_TRAJ37_F_61967238_61967325

TGCAGTGGTTATTCAAACACATTATGTGACTGATGGACGGAACAAAATCTACTTTGGGTTGGGCTCAAA
AGTTATTGTTCAGCCAG

>aOncmyk_Chr8_NC_048572_RS_TRAJ38_F_61967405_61967495

GGTTATTGTTGAGTAATAATTCAATGTGTGAATGCTAACAACCAGAAAGTCATATTTGGAGAAGGAAGT
AAATTGATAATTGAAACAAG

>aOncmyk_Chr8_NC_048572_RS_TRAJ39_F_61968290_61968383

CATTTTTGGTTAAGGCCCAAGCATTGTGTGACTGACTCTGGAATAAGGAAGGTCATTTTTGGAACAGGA
ATTGAATTGATCATTGAAACCAG

>aOncmyk_Chr8_NC_048572_RS_TRAJ40_F_61968909_61968998

TTCATGTAATGTGAGTTCTAGTTGTGTGACTGATGGCCTAAAAATAATGTTTGGAACAGGTACAAAATT
AATTGTTGACTCAAGTAAG

>aOncmyk_Chr8_NC_048572_RS_TRAJ41_F_61969104_61969200

AGTTTATGTGCTGTTGGTTCATGTTGTGTGACTGCTGGTGGTGGCCAAAGTAAAATTATCTTTGGGACA
GGCACACAATTAATAGTTGACTCAAG

>aOncmyk_Chr8_NC_048572_RS_TRAJ42_F_61969624_61969717

AGTTAATGTACTGTGGGTTCATGTTGTGTGACTACTGGTGGCAATGATAAAATCATCTTTGGGACAGGC
ACAAAATTAATAGTTGACTCAAG

>aOncmyk_Chr8_NC_048572_RS_TRAJ43_F_61969939_61970029

AGATATTGTTCAAGGCAGATGCATTGTGTGACTCAAGCAAGTGGGAAAGTAATCTTTGGAACAGGGACC
AAACTAGTTATTGAAATCAG

>aOncmyk_Chr8_NC_048572_RS_TRAJ44_F_61970784_61970874

GGTTTATGCTATGTGGGTTCTTGATGTGTGACTGGTGCAGCCAACAAAATCATCTTTGGGACAGGCACA
AAAGTAATAGTTAACTCAAG

>aOncmyk_Chr8_NC_048572_RS_TRAJ45_F_61971282_61971372

TGTTTTTGTTAAGCCTTTAGTTAGTGTGTGAATACTGGAACACGAACACTGATCTTTGGAAGTGGAACA
ACAGTTTTTGTGCAATCAAG

>aOncmyk_Chr8_NC_048572_RS_TRAJ46_F_61972083_61972179

AGTTTATGTTGTGTGGATTCATGTTGTGTGACAAGTGGAGGTAGTTACGGCAAACTTATCTTTGGGACA
GGCACCAGATTACTTATTGAATCCAG

>aOncmyk_Chr8_NC_048572_RS_TRAJ47_F_61972643_61972730

GGTTCTTGTAATGAAGCCATTGGCTGTGTGAATACTGGAAATAAGATAACATTTGGAGTTGGAACCCAA
CTAATTATACAAGCAAG

>aOncmyk_Chr8_NC_048572_RS_TRAJ48_F_61973030_61973120

GGTTATTGTTGAGGTCTAATTCAATGTGTGATTACTAACAACCAGAAAGTCATATTTGGAGCAGGAACT
AAATTGGTAATTGAAACAAG

>aOncmyk_Chr8_NC_048572_RS_TRAJ49_F_61973525_61973612

TTGCCAGTGTTTTTTGATCGGCTATGTGTGAATGATGGAATATGGAATCTGATCTTTGGAAGTGGAACA
CTATACATAGAATCTAG

>aOncmyk_Chr8_NC_048572_RS_TRAJ50_F_61973676_61973766

AGCAATTGCTAAGATATGCAACAATGTGTGACTTATGGAGACAACAAAGTTATATTTGGACTTGGCACA
AAGATGATGTTTTTTTCCAG

>aOncmyk_Chr8_NC_048572_RS_TRAJ51_F_61974233_61974312

TGTTTCTGTTATGTAAAATGTTACTGTATGAATGTTGGGACACGAAGACTGATCTTTGGCAGTGGAACA
ACACTCTAG

>aOncmyk_Chr8_NC_048572_RS_TRAJ52_F_61974509_61974602

ACTTTTTGGTTAAGGCACTAGCATTGTGTGACTGACTCTGGACTGAAGAAGGTCATTTTTGGAACAGGA
ATTGAATTGATCATTGAAACCAG

>aOncmyk_Chr8_NC_048572_RS_TRAJ53_F_61974983_61975079

AGTTTATGTGCTGTTGGTTCATGTTGTGTGACTACTGGTGGTGGCAACAGTAAAATTATCTTTGGGACA
GGCACACAATTAATAGTTGACTCAAG

>aOncmyk_Chr8_NC_048572_RS_TRAJ54_F_61975286_61975376

GGTTTATGTAATGTGGGTTCTAGTTGTGTGACTACTGATGGCCGAAAAATCATCTTTGGATCAGGCACA
AAATTAATAGTTGACTCAAG

>aOncmyk_Chr8_NC_048572_RS_TRAJ55_F_61975508_61975601

AGTTTATGTAATGTGGGTTCGTGTTGTGTGACTACTGGTGGCCTCGATAAAATCATCTTTGGGACAGGC
ACAAAATTAATAGTTGACTCAAG

>aOncmyk_Chr8_NC_048572_RS_TRAJ56_P_61975809_61975899

AGATATTGTTCAAGGCAGATGCATTGTCTGAGTTAAACAAGCAGAAAGGTCATCTTTGGAACAGGGACC
AAACTAGTTATTGAAACCAG

>aOncmyk_Chr8_NC_048572_RS_TRAJ57_F_61976102_61976193

AGTAATTGTTAAGGTGTCAAGCACTGTGTGACAAGTGGGAGCAAAGTCATATTCGGTCAAGGAACAAAG
CTGACCATTGACACACGTAAG

>aOncmyk_Chr8_NC_048572_RS_TRAJ58_F_61976415_61976508

AGTTTATGTCATGTGGAATCATGCTGTGTGACTACTGGAGTTGGTGACAAGATTATCTTTGGGAAAGGC
ACAATATTAATAATTCAATCCAG

>aOncmyk_Chr8_NC_048572_RS_TRAJ59_F_61977033_61977126

GTTTTATGTATTGTGAGTTCATGTTGTGTGACTAGTGGTGGATTCAACCAAATCATCTTTGGAACAGGC
ACAAAACTAATAGTTGATTCGAG

>aOncmyk_Chr8_NC_048572_RS_TRAJ60_F_61978095_61978189

AGATATTGTTCAAGGCAGATGCATTGTGTGACTCTGTCAAGCAGTAAAGTCATCTTTGGAACAGGGACC
AAACTAATTATTGAAACCAGTAAG

>aOncmyk_Chr8_NC_048572_RS_TRAJ61_F_61978659_61978767

AGTAATTGTTAAGATGTCTAGCACAGTGTGACAAGTGCGAACAAACTCATATTCGGTCAAGGAATTTAT
TTTTTTTATTTTTTTTTATTTTACCTTTATTTAACCAG

>aOncmyk_Chr8_NC_048572_RS_TRAJ62_F_61979001_61979094

AGTTTATGTCATATGGATTCATACTGTGTGACTACTGGAGGTGGTAACAAGATTGTCTTTGGGACAGGC
ACAAGATTAATAATTGAATCCAG

>aOncmyk_Chr8_NC_048572_RS_TRAJ63_F_61979832_61979923

AGTAATTGCTAAGGAATATAACACTGTGTGACAAGCACCAACAAGGTCATATTCGGTCAAGGAATAAAG
CTGACTATTAACTCACGTAAG

>aOncmyk_Chr8_NC_048572_RS_TRAJ64_F_61980048_61980138

GGTTTATGTAATGTGGGTTCTTGTTGTGTGAATACGGGTGGATTCAAAATCATCTTTGGGACAGGCACA
AAATTAATAGTGAACTCAAG

>aOncmyk_Chr8_NC_048572_RS_TRAJ65_F_61980371_61980461

GGTTTATGCTGTGTGGGTTCTTGATGTGTGACTCTTGGAGCCAACAAAATCATCTTTGGGACAGGCACA
AAAGTAATAGTTAACTCAAG

>aOncmyk_Chr8_NC_048572_RS_TRAJ66_F_61980843_61980933

TGTTTTTGTTAAGCCTTTAGTTAGTGTGTGAATACTGGAACAGAAAAGCTGATCTTTGGAAGTGGAACA
ACAGTTTTGTGCAATCAAG

>aOncmyk_Chr8_NC_048572_RS_TRAJ67_F_61981089_61981176

ATTAATTGCTATGGTATATACCACTGTGTGTCATACGGGAATAAATTCATACTTGGTCAAGGAACAAAG
CTGATCATTCACTCAAG

>aOncmyk_Chr8_NC_048572_RS_TRAJ68_F_61981543_61981639

AGTTTATGTTGTGTGGATTCATGTTGTGTGACAACTGGAGGTGGTTCTAGCAAGCTTATCTTTGGGACA
GGCACCAGATTACTTATTGAATCCAG

>aOncmyk_Chr8_NC_048572_RS_TRAJ69_F_61982200_61982290

TGTAAATGTATTGTAATCTACCACTGTGTGTCTAACTCTGGAGGGAAGCTTACATTTGGAACAGGAACA
AAGCTAATAATTGAGACAAG

>aOncmyk_Chr8_NC_048572_RS_TRAJ70_F_61982387_61982484

GGTTTTTAGCTGGTAACAAAATACTGTGTGACAAGAGGGAATAGTAACAGGCTTATCTTTGGAAACGGA
ACCCAACTGTTAGTTGAATCTGGTAAG

>aOncmyk_Chr8_NC_048572_RS_TRAJ71_F_61982584_61982679

TATTTGGTAAGGTGTGTAGCTCAGTGTGACTGCCCAGGGGGGATACAAGCTTATTTTTGGAAGTGGAAC
TAAAGTGATTGTAGAGACACGTAAG

>aOncmyk_Chr8_NC_048572_RS_TRAJ72_F_61983092_61983180

TTTATATACTGGTATGTTAGGCTGTGTGAATGATGGCAGTACAAAGATCATTTTTGGGAGAGGCACAAG
GCTACTAGTACTAAGCAG

>aOncmyk_Chr8_NC_048572_RS_TRAJ73_F_61983316_61983412

TGTAATTGTACTGCAGTTTAATACTGTGTGGCTACTCTGACAGCAGGAAAACTTGTGTTTGGTAGTGGA
ACCAAACTTTTCATAAATACTGGTAG

>aOncmyk_Chr8_NC_048572_RS_TRAJ74_F_61983670_61983764

GGTTTTTAGTGAGCAAGAATACACTGTGTGACAAGAACAAGTGACAAAATTACTTTTGCACGTGGGACT
CAACTGTTTGTCGAGAAAGGTGAG

>aOncmyk_Chr8_NC_048572_RS_TRAJ75_F_61984005_61984100

TAATTGTCATGGTCTGTATCTCAGTGTGAATTCCCAGGGGGGAGTAAAGCTTATTTTTGGGAGTGGAAC
AAAAATGATTGTGGAGACACGTAAG

>aOncmyk_Chr8_NC_048572_RS_TRAJ76_F_61984430_61984522

TTTTTATACTGGTGTGTCAGGCTGTGTGAATGATGCCGGTACAAAGATCATTTTTGGGAGAGGCACAAG
GCTACTAGTATTAAGTGGTAAG

>aOncmyk_Chr8_NC_048572_RS_TRAJ77_F_61984646_61984742

TGTAATTGTACTGCAGTTTAATGCTGTGTGGCTACTCTGACAGCAGGAAAACTTGTGTTTGGTAGTGGA
ACCAAACTTTTCATAAATACTGGTAG

>aOncmyk_Chr8_NC_048572_RS_TRAJ78_F_61985287_61985382

TAATTGCCATGGTCTGTATCTCAGTGTGAATGCCCAGGTGGGAGGAAAGCTTATTTTTGGGAGTGGAAC
AAAAATGATTGTGGGGACACGTAAG

>aOncmyk_Chr8_NC_048572_RS_TRAJ79_F_61985971_61986067

TGTAATTGTACTGCAGTTTAACACTGTGTGACTACTGGAACAACAGGAAAATTAATATTTGGAAGTGGAACCAAACTGTTCATAAATACTGGTAG

>aOncmyk_Chr8_NC_048572_RS_TRAJ80_F_61986569_61986659

TAATTGTCATTGTCTGTATCTCAGTGTGAATGCTCAGGGGGCAGGAAAGATACATTTTGGGAATGGAATCAAAATGATTGTGGGAACAC

>aOncmyk_Chr8_NC_048572_RS_TRAJ81_F_61986911_61987007

GATTTGTGTACAGGAGTGTAGCACTGTGGTCGGCTGGCTCTAACAATAAGCTGATTTTTGGATCTGGAGTGAATGTCATTGTGCAATCCCGTAAG

Oncorhynchus mykiss (Swanson strain)

>sOncmyk_Chr8_NC_035084 _SS_TRAJ1_P_57436709_57436799

TGGTATTGCTCACAGCTAATGAACTGTGAGTGACTGGAACAGGAATCAATAAACTTGTTTTTGGAACTGCGGTAAAGATCACTATTGAG

>sOncmyk_Chr8_NC_035084 _SS_TRAJ2_F_57437148_57437235

TGTTTTTGCATGAGAATAAGTTATTGTGTGAATGAGTATCAGAAGATCACATTTGGTTCAGGAACCAAAATGTTCATTATGACAAG

>sOncmyk_Chr8_NC_035084 _SS_TRAJ3_F_57437481_57437568

TGTTTTTGTAATGAGGCCAATGTCTGTGTGACAAGTGGATTCAAAATATCATTTGGAAATGGCATCCAACTAATTGTGCATTCAAG

>sOncmyk_Chr8_NC_035084 _SS_TRAJ4_F_57441774_57441861

GGTTCTTGTAATGAGGCCTTTGTCTGTGTGAATACCGGATATAAGATAACATTTGGAGTTGGAACCCAACTAATTATACAAGCAAG

>sOncmyk_Chr8_NC_035084 _SS_TRAJ5_F_57442155_57442245

CGTTATTGCTGAGTACTAATACAATGTGTGAATGCTAACAACCGGAAAGTCATATTTGGAGTAGGAACTAAATTGATAATTGAAATAAG

>sOncmyk_Chr8_NC_035084 _SS_TRAJ6_F_57442644_57442734

AGTAATTGCTAAGAGATGCAACACAGTGTGACTTACGGAGCCAACAAACTTATATTTGGACTTGGCACAAAGATGACAGTTTATTCCAG

>sOncmyk_Chr8_NC_035084 _SS_TRAJ7_F_57446014_57446102

TTTATGCAATGGGGCAAAGTGTTGTGTGAATTCTGGATATGGCAAAATAACATTTGGGAGCGGCACAAAATTGATAATAGATTCAAG

>sOncmyk_Chr8_NC_035084 _SS_TRAJ8_F_57446452_57446539

GGTTTTTGTAATGAGGCCATTGCCTGTGTGAATACTGGAAATAAGATCATATTTGGAGTCGGAACCCAACTAATTATACAAGCAAG

>sOncmyk_Chr8_NC_035084 _SS_TRAJ9_F_57446676_57446771

TTTAGTTTTCACATTTTCAAATTATGTGAATGATATATGGAACAAAGTCTACTTTGGGCTGGGAACTTG
TTCAGCCAGGTTAAAATTGTATGAG

>sOncmyk_Chr8_NC_035084 _SS_TRAJ10_F_57449031_57449118

TGTATTTGCTTAGATAACAAACACTGTGTGACTGTTGGGGTCAGAGTTGTTTTTGGGCATGGAACAAAG
TTGACGGTCAATCCAAG

>sOncmyk_Chr8_NC_035084 _SS_TRAJ11_F_57449748_57449837

CTTATGTAATGTGGGTTCTAGTTGTGTGGCTACTGATGGCCTAAAAATCGTCTTTGGATCAGGCACAAA
ATTAATTGTTGACTCAAG

>sOncmyk_Chr8_NC_035084 _SS_TRAJ12_F_57449963_57450054

AGTAATTGCTAAGGGATCTAGCACAGTGTGACAAGTGCGAACAAACTCATATTCGGTCAAGGAATAAAG
CTGACTGTTGACACACGTAAG

>sOncmyk_Chr8_NC_035084 _SS_TRAJ13_F_57450712_57450805

GGTTTATGTAATGTAGGTTCATGTTGTGTGACTGCTGATGGATTCAACAAAATCATCTTTGGGACAGGC
ACAAAATTAATAGTTTACTCAAG

>sOncmyk_Chr8_NC_035084 _SS_TRAJ14_F_57451350_57451444

GGTTAATGTTGTGTCGGTTCTTGTTGTGTGAATGCGAATGGATTCAAAATCATCTTTGGGACAGGCACA
ACATTAATAGTTAACTCAAGTAAG

>sOncmyk_Chr8_NC_035084 _SS_TRAJ15_F_57452308_57452402

TGTTTTTGTTAAGCCTTTAGTTAGTGTGTGAATATTGGCGGACAAAAGCTGATCTTTGGAAGTGGAACA
ACAGTTTTTGTGGAATCAAGTAAG

>sOncmyk_Chr8_NC_035084 _SS_TRAJ16_F_57453599_57453689

GGTTCTTGTTGAGTACTAATTCAATGTGTGAATGATAACAACTGGAAAGTCATATTTGGAGCAGGAACT
AAATTGATCATCGAAAAAAG

>sOncmyk_Chr8_NC_035084 _SS_TRAJ17_F_57455363_57455456

CATTTTTAGTTCAGCCCCAATCATTGTGTGACTAACTCAGGACTGGGGAAGGTCATTTTTGGAGCAGGA
ATTGAATTGATCATTGAAACCAG

>sOncmyk_Chr8_NC_035084 _SS_TRAJ18_F_57455807_57455897

CATTTTTGTTGAGCCTGTTTTTAGTGTGTGAATGATGGAGTGAGAAAGCTCATCTTTGGGAGTGGAACA
TCACTATCTGTAGAGACAAG

>sOncmyk_Chr8_NC_035084 _SS_TRAJ19_F_57456042_57456135

ATGTATTTGGTAAATATCAAACATTGTGTGACTTTGGAGTCAACAGAGTTGTGTTTGGGCATGGACAA
TGTTGACGGTCAATTCATGCAAG

>sOncmyk_Chr8_NC_035084 _SS_TRAJ20_F_57456362_57456452

AGTTCTTGCTCAGCCTGTTTTTAGTGTGTGTATGCTGGAGGAGGAAAGTTCATCTTTGGCAGTGGAACA
TCACTGTTTGTAAAAACAAG

>sOncmyk_Chr8_NC_035084 _SS_TRAJ21_F_57457034_57457127

AGTTAATGTACTGTGGGTTCATGTTGTGTGACTACTGGTGGCAATGTTAAACTTATCTTTGGGAGAGGCACACAATTAATAGTTGACTCAAG

>sOncmyk_Chr8_NC_035084 _SS_TRAJ22_F_57457303_57457393

AGATATTGTTCAAGGCAGATGCATTGTGTGACTCAAACAAGTAGGAAAGTCATCTTTGGAACAGGGACCAAACTAGTTATTGAAACCAGTA

>sOncmyk_Chr8_NC_035084 _SS_TRAJ23_P_57457624_57457716

AGTAATTGATAAGTGATCTAACACTGTGTGACAAGTGCGAAAAAAACGTATATTCGGTCAAGGAATAAAGGTGACTATTGACTCACGTAAG

>sOncmyk_Chr8_NC_035084 _SS_TRAJ24_F_57458860_57458950

GGTTTATGCTATGTGGGTTCTTGATGTGTGATTGGTGGAGCCAACAAAATCATCTTTGGGACAGGCACAAAAGTAATAGTTAACTCAAG

>sOncmyk_Chr8_NC_035084 _SS_TRAJ25_F_57459588_57459679

ATTAATTGCTGGGGTATCTAACACTGTGTGTCATATGGGAATACATTCATACTTGGTCAAGGAACAAAGCTGATCATTGACTCAAGTAAG

>sOncmyk_Chr8_NC_035084 _SS_TRAJ26_F_57460145_57460241

AGTTTATGTTGTGTGGATTCATGTTGTGTGACAAGTGGAGGTAGTTACGGCAAACTTATCTTTGGGACAGGCACCAGATTACTTATTGAATCCAG

>sOncmyk_Chr8_NC_035084 _SS_TRAJ27_F_57460899_57460986

TGCAGTGGGTATTCGAACACATTATGTGACTGATGGACGGAACAAAATCTACTTTGGGTTGGGCTCAAAAGTTATTGTTCAGCCAG

>sOncmyk_Chr8_NC_035084 _SS_TRAJ28_F_57461066_57461156

GGTTATTGTTGAGTAATAATTCAATGTGTGAATGCTAACAACCAGAAAGTCATATTTGGAGAAGGAAGTAAATTGATAATTGAAACAAG

>sOncmyk_Chr8_NC_035084 _SS_TRAJ29_F_57461951_57462044

CATTTTTGGTTAAGGCCCAAGCATTGTGTGACTGACTCTGGAATAAGGAAGGTCATTTTTGGAACAGGAATTGAATTGATCATTGAAACCAG

>sOncmyk_Chr8_NC_035084 _SS_TRAJ30_F_57462574_57462659

TTCATGTAATGTGAGTTCTAGTTGTGTGACTGATGGCCTAAAAATAATGTTTGGAACAGGTACAAAATTAATTGTTGACTCAAG

>sOncmyk_Chr8_NC_035084 _SS_TRAJ31_F_57462765_57462861

AGTTTATGTGCTGTTGGTTCATGTTGTGTGACTGCTGGTGGTGGCCAAAGTAAAATTATCTTTGGGACAGGCACACAATTAATAGTTGACTCAAG

>sOncmyk_Chr8_NC_035084 _SS_TRAJ32_F_57463285_57463378

AGTTAATGTACTGTGGGTTCATGTTGTGTGACTACTGGTGGCAATGATAAAATCATCTTTGGGACAGGCACAAAATTAATAGTTGACTCAAG

>sOncmyk_Chr8_NC_035084 _SS_TRAJ33_F_57464445_57464534

GTTTATGCTATGTGGGTTCTTGATGTGTGACTGGTGCAGCCAACAAAATCATCTTTGGGACAGGCACAA
AAGTAATAGTTAACTCAAG

>sOncmyk_Chr8_NC_035084 _SS_TRAJ34_F_57464943_57465033

TGTTTTTGTTAAGCCTTTAGTTAGTGTGTGAATACTGGAACACGAACACTGATCTTTGGAAGTGGAACA
ACAGTTTTTGTGCAATCAAGTA

>sOncmyk_Chr8_NC_035084 _SS_TRAJ35_F_57465744_57465840

AGTTTATGTTGTGTGGATTCATGTTGTGTGACAAGTGGAGGTAGTTACGGCAAACTTATCTTTGGGACA
GGCACCAGATTACTTATTGAATCCAG

>sOncmyk_Chr8_NC_035084 _SS_TRAJ36_F_57466304_57466391

GGTTCTTGTAATGAAGCCATTGGCTGTGTGAATACTGGAAATAAGATAACATTTGGAGTTGGAACCCAA
CTAATTATACAAGCAAG

>sOncmyk_Chr8_NC_035084 _SS_TRAJ37_F_57466691_57466781

GGTTATTTTTGAGGTCTAATTCAATGTGTGATTACTAACAACCAGAAAGTCATATTTGGAGCAGGAACT
AAATTGGTAATTGAAACAAG

>sOncmyk_Chr8_NC_035084 _SS_TRAJ38_F_57467186_57467273

TTGCCAGTGTTTTTCGATCTGCTATGTGTGAATGATGGAATATGGAATCTGATCTTTGGAAGTGGAACA
CTATACATAGAATCTAG

>sOncmyk_Chr8_NC_035084 _SS_TRAJ39_F_57467337_57467427

AGCAATTGCTAAGATATGCAACAATGTGTGACTTATGGAGACAACAAAGTTATATTTGGACTTGGCACA
AAGATGATGTTTTTTTCCAG

>sOncmyk_Chr8_NC_035084 _SS_TRAJ40_F_57467894_57467973

TGTTTCTGTTATGTAAAATGTTACTGTATGAATGTTGGGACACGAAGACTGATCTTTGGCAGTGGAACA
ACACTCTAGTA

>sOncmyk_Chr8_NC_035084 _SS_TRAJ41_F_57468170_57468263

ACTTTTTGGTTAAGGCACTAGCATTGTGTGACTGACTCTGGACTGAAGAAGGTCATTTTTGGAACAGGA
ATTGAATTGATCATTGAAACCAG

>sOncmyk_Chr8_NC_035084 _SS_TRAJ42_F_57468644_57468740

AGTTTATGTGCTGTTGGTTCATGTTGTGTGACTACTGGTGGTGGCAACAGTAAAATTATCTTTGGGACA
GGCACACAATTAATAGTTGACTCAAG

>sOncmyk_Chr8_NC_035084 _SS_TRAJ43_F_57469168_57469259

TTTATGTAATGTGGGTTCTAGTTGTGTGACTACTGGTGGCCTCGATAAAATCATCTTTGGGACAGGCAC
AAAATTAATAGTTGACTCAAG

>sOncmyk_Chr8_NC_035084 _SS_TRAJ44_F_57469762_57469853

AGTAATTGTTAAGGTGTCAAGCACTGTGTGACAAGTGGGAGCAAAGTCATATTCGGTCAAGGAACAAAG
CTGACCATTGACACACGTAAG

>sOncmyk_Chr8_NC_035084 _SS_TRAJ45_F_57470701_57470794

GTTTTATGTATTGTGAGTTCATGTTGTGTGACTAGTGGTGGATTCAACCAAATCATCTTTGGAACAGGC
ACAAAACTAATAGTTGATTCGAG

>sOncmyk_Chr8_NC_035084 _SS_TRAJ46_F_57471763_57471857

AGATATTGTTCAAGGCAGATGCATTGTGTGACTCTGTCAAGCAGTAAAGTCATCTTTGGAACAGGGACC
AAACTAATTATTGAAACCAGTAAG

>sOncmyk_Chr8_NC_035084 _SS_TRAJ47_F_57472454_57472547

AGTTTATGTCATATGGATTCATACTGTGTGACTACTGGAGGTGGTAACAAGATTGTCTTTGGGACAGGC
ACAAGATTAATAATTGAATCCAG

>sOncmyk_Chr8_NC_035084 _SS_TRAJ48_F_57473286_57473377

AGTAATTGCTAAGGAATATAACACTGTGTGACAAGCACCAACAAGGTCATATTCGGTCAAGGAATAAAG
CTGACTATTAACTCACGTAAG

>sOncmyk_Chr8_NC_035084 _SS_TRAJ49_F_57473502_57473592

GGTTTATGTAATGTGGGTTCTTGTTGTGTGAATACGGGTGGATTCAAAATCATCTTTGGGACAGGCACA
AAATTAATAGTGAACTCAAG

>sOncmyk_Chr8_NC_035084 _SS_TRAJ50_F_57473825_57473915

GGTTTATGCTGTGTGGGTTCTTGATGTGTGACTCTTGGAGCCAACAAAATCATCTTTGGGACAGGCACA
AAAGTAATAGTTAACTCAAG

>sOncmyk_Chr8_NC_035084 _SS_TRAJ51_F_57474297_57474387

TGTTTTTGTTAAGCCTTTAGTTAGTGTGTGAATACTGGAACAGAAAAGCTGATCTTTGGAAGTGGAACA
ACAGTTTTTGTGCAATCAAG

>sOncmyk_Chr8_NC_035084 _SS_TRAJ52_F_57474543_57474630

ATTAATTGCTATGGTATATACCACTGTGTGTCATACGGGAATAAATTCATACTTGGTCAAGGAACAAAG
CTGATCATTCACTCAAG

>sOncmyk_Chr8_NC_035084 _SS_TRAJ53_F_57474997_57475093

AGTTTATGTTGTGTGGATTCATGTTGTGTGACAACTGGAGGTGGTTCTAGCAAGCTTATCTTTGGGACA
GGCACCAGATTACTTATTGAATCCAG

>sOncmyk_Chr8_NC_035084 _SS_TRAJ54_F_57475650_57475744

TGTAAATGTATTGTAATCTACCACTGTGTGTCTAACTCTGGAGGGAAGCTTACATTTGGAACAGGAACA
AAGCTAATAATTGAGACAAGTAAG

>sOncmyk_Chr8_NC_035084 _SS_TRAJ55_F_57475841_57475938

GGTTTTTAGCTGGTAACAAAATACTGTGTGACAAGAGGGAATAGTAACAGGCTTATCTTTGGAAACGGA
ACCCAACTGTTAGTTGAATCTGGTAAG

>sOncmyk_Chr8_NC_035084 _SS_TRAJ56_F_57476038_57476133

TATTTGGTAAGGTGTGTAGCTCAGTGTGACTGCCCAGGGGGGATACAAGCTTATTTTTGGAAGTGGAAC
TAAAGTGATTGTAGAGACACGTAAG

>sOncmyk_Chr8_NC_035084 _SS_TRAJ57_F_57476541_57476635

AGTTTATATACTGGTATGTTAGGCTGTGTGAATGATGGCAGTACAAAGATCATTTTTGGGAGAGGCACA
AGGCTACTAGTACTAAGCAGTAAG

>sOncmyk_Chr8_NC_035084 _SS_TRAJ58_F_57477128_57477217

GGTTTTTAGTGAGCAAGAATACACTGTGTGACAAGAACAAGTGACAAAATTACTTTTGCACGTGGGACT
CAACTGTTTGTCGAGAAAG

>sOncmyk_Chr8_NC_035084 _SS_TRAJ59_F_57477458_57477553

TAATTGTCATGGTCTGTATCTCAGTGTGAATTCCCAGGGGGGAGTAAAGCTTATTTTTGGGAGTGGAAC
AAAAATGATTGTGGAGACACGTAAG

>sOncmyk_Chr8_NC_035084 _SS_TRAJ60_F_57477883_57477977

AGTTTTTATACTGGTGTGTCAGGCTGTGTGAATGATGCCGGTACAAAGATCATTTTTGGGAGAGGCACA
AGGCTACTAGTATTAAGTGGTAAG

>sOncmyk_Chr8_NC_035084 _SS_TRAJ61_F_57478099_57478195

TGTAATTGTACTGCAGTTTAATGCTGTGTGGCTACTCTGACAGCAGGAAAACTTGTGTTTGGTAGTGGA
ACCAAACTTTTCATAAATACTGGTAG

>sOncmyk_Chr8_NC_035084 _SS_TRAJ62_F_57478740_57478835

TAATTGTCATGGTCTGTATCTCAGTGTGAATGCCCAGGTGGGAGGAAAGCTTATTTTTGGGAGTGGAAC
AAAAATGATTGTGGGGACACGTAAG

>sOncmyk_Chr8_NC_035084 _SS_TRAJ63_F_57479424_57479520

TGTAATTGTACTGCAGTTTAACACTGTGTGACTACTGGAACAACAGGAAAATTAATATTTGGAAGTGGA
ACCAAACTGTTCATAAATACTGGTAG

>sOncmyk_Chr8_NC_035084 _SS_TRAJ64_F_57480017_57480112

TAATTGTCATGGTCTGTATCTCAGTGTGAATGCTCAGGGGGCAGGAAAGATACATTTTGGGAATGGAAT
CAAAATGATTGTGGGAACACGTAAG

>sOncmyk_Chr8_NC_035084 _SS_TRAJ65_F_57480364_57480460

GATTTGTGTACAGGAGTGTAGCACTGTGGTCGGCTGGCTCTAACAATAAGCTGATTTTTGGATCTGGAG
TGAATGTCATTGTGCAATCCCGTAAG

Deduced amino acid sequences of TRAJ genes. Sequences were aligned with ClustalW. The motif FGXG (X is any amino acid) is shown in bold blue. Partial motif is shown in bold grey.

Salsal_TRAJ1_ORF	VSGTGINKLV***FGTA***VKITIEAS
sOncmyk_TRAJ1_P	VTGTGINKLV***FGTA***VKITIEAS
aOncmyk_TRAJ1_ORF	VTGTGINKLV***FGTA***VKITIEAS
Salsal_TRAJ2_F	NEYQKIT***FGSG***TKLVIMTS
sOncmyk_TRAJ2_F	NEYQKIT***FGSG***TKMFIMTS
aOncmyk_TRAJ2_F	NEYQKIT***FGSG***TKMFIMTSK
Salsal_TRAJ120_F	SAQGGYKLI***FGSG***TKVIVETRK
sOncmyk_TRAJ56_F	TAQGGYKLI***FGSG***TKVIVETRK
aOncmyk_TRAJ71_F	TAQGGYKLI***FGSG***TKVIVETRK
Salsal_TRAJ124_F	NAQGGVKLI***FGSG***TKMIVETRK
sOncmyk_TRAJ59_F	NSQGGVKLI***FGSG***TKMIVETRK
aOncmyk_TRAJ75_F	NSQGGVKLI***FGSG***TKMIVETRK
sOncmyk_TRAJ62_F	NAQVGGKLI***FGSG***TKMIVGTRK
aOncmyk_TRAJ78_F	NAQVGGKLI***FGSG***TKMIVGTRK
Salsal_TRAJ127_F	NAQGAGKIY***FGNG***IKMIVGTR
aOncmyk_TRAJ80_F	NAQGAGKIH***FGNG***IKMIVGTR
sOncmyk_TRAJ64_F	NAQGAGKIH***FGNG***IKMIVGTRK
Salsal_TRAJ128_F	LAGSNNKLI***FGSG***VNVIVQSR
sOncmyk_TRAJ65_F	SAGSNNKLI***FGSG***VNVIVQSRK
aOncmyk_TRAJ81_F	SAGSNNKLI***FGSG***VNVIVQSRK
Salsal_TRAJ12_ORF	TYGANKLI***IGLG***TKMTVYSSK
sOncmyk_TRAJ6_F	TYGANKLI***FGLG***TKMTVYS
aOncmyk_TRAJ6_F	TYGANKLI***FGLG***TKMTVYSSK
Salsal_TRAJ25_F	TYEADKFI***FGLG***TKITVYSS
Salsal_TRAJ91_F	TYGDSKVI***FGLG***TKMFFSQ
sOncmyk_TRAJ39_F	TYGDNKVI***FGLG***TKMMFFS
aOncmyk_TRAJ50_F	TYGDNKVI***FGLG***TKMMFFS
Salsal_TRAJ65_ORF	TASGIQNVP***LGSR***TKLLIETR
Salsal_TRAJ92_F	TASGIQKVL***FGSG***TKLFIETS
Salsal_TRAJ26_F	TAFGIQKVL***FGSG***TKLVIKTRE
sOncmyk_TRAJ61_F	ATLTAGKLV***FGSG***TKLFINTG
aOncmyk_TRAJ73_F	ATLTAGKLV***FGSG***TKLFINTG
aOncmyk_TRAJ77_F	ATLTAGKLV***FGSG***TKLFINTG
Salsal_TRAJ122_F	NTGSAGKLV***FGSG***AKLFINTGR
Salsal_TRAJ125_F	TTGTNGKLI***FGSG***TKLFINTG
sOncmyk_TRAJ63_F	TTGTTGKLI***FGSG***TKLFINTG
aOncmyk_TRAJ79_F	TTGTTGKLI***FGSG***TKLFINTG
Salsal_TRAJ11_F	NANNRKVI***FGAG***TKLIIEIS
sOncmyk_TRAJ5_F	NANNRKVI***FGVG***TKLIIEIS
aOncmyk_TRAJ5_F	NANNRKVI***FGVG***TKLIIEIS
Salsal_TRAJ23_F	NGNNRKVI***FGAG***TKLIIETS
aOncmyk_TRAJ12	NGNNRKVI***FGAG***TKLIIETS
Salsal_TRAJ41_F	NDNNWKVI***FGAG***TKLIIERS
sOncmyk_TRAJ16_	NDNNWKVI***FGAG***TKLIIEK
aOncmyk_TRAJ22_	NDNNWKVI***FGAG***TKLIIEK
Salsal_TRAJ21_F	NVNNWKVI***FGAG***TKLIIESS
aOncmyk_TRAJ10_ORF	NVNNWKVI***LGAG***TKLIIESS
Salsal_TRAJ64_F	NDNNQKVI***FGEG***SKLIIETS
sOncmyk_TRAJ28_F	NANNQKVI***FGEG***SKLIIETS
aOncmyk_TRAJ38_F7	NANNQKVI***FGEG***SKLIIETS
Salsal_TRAJ89_F	ITNNQKVI***FGAG***TKLVIETS
sOncmyk_TRAJ37_F	ITNNQKVI***FGAG***TKLVIETS
aOncmyk_TRAJ48_F	ITNNQKVI***FGAG***TKLVIETS
Salsal_TRAJ45_F	TDSGLGKVI***FGAG***IELVIETS

Salsal_TRAJ67_F	TDSGLGKVI***FGAG***IELVIETS
Salsal_TRAJ94_F	TDSGLKKVI***FGTG***IELIIETS
Salsal_TRAJ14_F	TDSGLGKVI***FGTG***IELIIETSE
sOncmyk_TRAJ29_F	TDSGIRKVI***FGTG***IELIIETSE
aOncmyk_TRAJ39_F	TDSGIRKVI***FGTG***IELIIETSE
sOncmyk_TRAJ41_F	TDSGLKKVI***FGTG***IELIIETSE
aOncmyk_TRAJ52_F	TDSGLKKVI***FGTG***IELIIETSE
sOncmyk_TRAJ17_F	TNSGLGKVI***FGAG***IELIIETSE
aOncmyk_TRAJ24_F	TNSGLGKVI***FGAG***IELIIETSE
Salsal_TRAJ99_P	TTSRKVI***FGTG***TKLVIETSK
Salsal_TRAJ80_F	TQTSRKVI***FGTG***TKLIVEIS
Salsal_TRAJ51_F	TQTSRKVI***FGTG***TKLIVEIS
aOncmyk_TRAJ43_F	TQASGKVI***FGTG***TKLVIEIS
sOncmyk_TRAJ22_F	TQTSRKVI***FGTG***TKLVIETS
aOncmyk_TRAJ29_F	TQTSRKVI***FGTG***TKLVIETS
aOncmyk_TRAJ56_P	SXATSRKVI***FGTG***TKLVIET
Salsal_TRAJ105_F	TLSSSKVI***FGTG***TKLIIETSK
sOncmyk_TRAJ46_F	TLSSSKVI***FGTG***TKLIIETSK
aOncmyk_TRAJ60_F	TLSSSKVI***FGTG***TKLIIETSK
Salsal_TRAJ116_F	SNAGGKLT***FGTG***TKLIIETSK
sOncmyk_TRAJ54_F	SNSGGKLT***FGTG***TKLIIETSK
aOncmyk_TRAJ69_F	SNSGGKLT***FGTG***TKLIIETS
Salsal_TRAJ31_F	TSANKVI***FGQG***IKLTIDTRK
Salsal_TRAJ81_F	TSANKLI***FGQG***IKVTIDSR
Salsal_TRAJ106_F	TSANKVI***FGQG***IKLTIDSRK
aOncmyk_TRAJ17_F	TSANKLI***FGQG***IKLTVDTRK
sOncmyk_TRAJ12_F	TSANKLI***FGQG***IKLTVDTRK
sOncmyk_TRAJ48_F	TSTNKVI***FGQG***IKLTINSRK
aOncmyk_TRAJ63_F	TSTNKVI***FGQG***IKLTINSRK
Salsal_TRAJ109_F	TSTNKVI***FGQG***IKLTINSRK
sOncmyk_TRAJ23_P	XAQVRKKRI***FGQG***IKVTIDSRK
aOncmyk_TRAJ30_ORF	XAQVRKKRI***FGQG***IKVTIDSRK
Salsal_TRAJ100_F	TSGSKVI***FGQG***TKLTIDSRE
sOncmyk_TRAJ44_F	TSGSKVI***FGQG***TKLTIDTRK
aOncmyk_TRAJ57_F	TSGSKVI***FGQG***TKLTIDTRK
Salsal_TRAJ113_ORF	KYGNKFI***LGQG***TKLIIHSSK
Salsal_TRAJ58_ORF	SYGNKFI***LGQG***TKLIIDSS
Salsal_TRAJ72_ORF	SYGNKFI***LGQG***IKLIIDSS
Salsal_TRAJ85_P	YGNKFI***LGQG***TKLIIDSS
sOncmyk_TRAJ25_ORF	SYGNTFI***LGQG***TKLIIDSSK
aOncmyk_TRAJ34_ORF	SYGNTFI***LGQG***TKLIIDSSK
sOncmyk_TRAJ52_ORF	SYGNKFI***LGQG***TKLIIHSS
aOncmyk_TRAJ67_ORF	SYGNKFI***LGQG***TKLIIHSS
Salsal_TRAJ3_F	TSGFKIS***FGNG***IQLIVHSS
sOncmyk_TRAJ3_F	TSGFKIS***FGNG***IQLIVHSS
aOncmyk_TRAJ3_F	TSGFKIS***FGNG***IQLIVHSS
Salsal_TRAJ35_F	NTNGFKII***FGTG***TQLIVNSS
sOncmyk_TRAJ14_F	NANGFKII***FGTG***TTLIVNSSK
aOncmyk_TRAJ20_F	NANGFKII***FGTG***TTLIVNSSK
sOncmyk_TRAJ49_F	NTGGFKII***FGTG***TKLIVNSS
aOncmyk_TRAJ64_F	NTGGFKII***FGTG***TKLIVNSS
Salsal_TRAJ110_P	NTGGFKII***FGTG***SNTQVS
Salsal_TRAJ69_P	TDGLKIV***FGSG***TKIVDSS
Salsal_TRAJ76_P	TDGLKIV***FGSG***TKIVDSS
Salsal_TRAJ95_P	TDGLKIM***FSG***TKLIVDSSK
sOncmyk_TRAJ11_F	ATDGLKIV***FGSG***TKLIVDSS
aOncmyk_TRAJ16_F	ATDGLKIV***FGSG***TKLIVDSS
sOncmyk_TRAJ30_F	TDGLKIM***FGTG***TKLIVDSSK

aOncmyk_TRAJ40_F	TDGLKIM***FGTG***TKLIVDSSK
Salsal_TRAJ30_F	TTDGLKII***FGSG***TKLIVDSS
Salsal_TRAJ78_F	TTDGRKIL***FGSG***TKLIVDSS
aOncmyk_TRAJ54_F	TTDGRKII***FGSG***TKLIVDSS
Salsal_TRAJ97_F	TTAGRKIL***FGSG***TKLIVDSS
Salsal_TRAJ49_ORF	IAEGLKMI***FGSV***TTLIVDSSK
Salsal_TRAJ9_F	NTGYKII***FGVG***TQLIIQAS
sOncmyk_TRAJ4_F	NTGYKIT***FGVG***TQLIIQAS
aOncmyk_TRAJ4_F	NTGYKIT***FGVG***TQLIIQAS
aOncmyk_TRAJ36_F	NTGYKIT***FGVG***TQLIVQAS
sOncmyk_TRAJ36_F	NTGNKIT***FGVG***TQLIIQAS
aOncmyk_TRAJ47_F	NTGNKIT***FGVG***TQLIIQAS
Salsal_TRAJ61_F	NTGYKII***FGVG***TQLIIQAS
Salsal_TRAJ19_F	NTGNKII***FGVG***TKLIIQAS
sOncmyk_TRAJ8_F	NTGNKII***FGVG***TQLIIQAS
aOncmyk_TRAJ8_F	NTGNKII***FGVG***TQLIIQAS
Salsal_TRAJ88_F	NTGNKLT***FGVG***TQLIIQSS
Salsal_TRAJ40_F	NAGYKII***FGVG***TQLIVQAS
Salsal_TRAJ107_F	TTGGGNKIV***FGTG***TRLIIESS
sOncmyk_TRAJ47_F	TTGGGNKIV***FGTG***TRLIIESS
aOncmyk_TRAJ62_F	TTGGGNKIV***FGTG***TRLIIESS
Salsal_TRAJ32_F	TTGGGYKIV***FGTG***TRLIIKSS
aOncmyk_TRAJ18_F	TTGGGYKIV***FGTG***TRLIIKSS
Salsal_TRAJ82_F	TVGGGSKII***FGTG***TRLILKSS
aOncmyk_TRAJ31_F	TVGGGSKIT***FGTG***TRLILKSS
Salsal_TRAJ101_F	TTGVGDKII***FGTG***TRLIIQSS
aOncmyk_TRAJ58_F	TTGVGDKII***FGKG***TILIIQSS
Salsal_TRAJ53_F	TAGSGTKII***FGKG***TRLIIESS
Salsal_TRAJ18_F	NSGYGKIT***FGSG***TKLIIDSS
sOncmyk_TRAJ7_F	NSGYGKIT***FGSG***TKLIIDSS
aOncmyk_TRAJ7_F	NSGYGKIT***FGSG***TKLIIDSS
Salsal_TRAJ5_F	NSGFDKVT***FGSG***TKLIIDSS
Salsal_TRAJ39_F	NSGNDKIT***FGSG***TKCIIDSS
Salsal_TRAJ102_F	TTAGVNKVI***FGTG***TKLIIDSS
Salsal_TRAJ33_F	TADGFNKII***FGTG***TKLIVYSS
sOncmyk_TRAJ13_F	TADGFNKII***FGTG***TKLIVYSS
aOncmyk_TRAJ19_F	TADGFNKII***FGTG***TKLIVYSS
sOncmyk_TRAJ45_F	TSGGFNQII***FGTG***TKLIVDSS
aOncmyk_TRAJ59_F	TSGGFNQII***FGTG***TKLIVDSS
Salsal_TRAJ103_F	TSGGFNQII***FGTG***TKRIVYSS
Salsal_TRAJ55_F	TSGGLNKIV***FGKG***IKLTVASS
Salsal_TRAJ79_F	TTGSNVKII***FGRG***TQLIVDSS
Salsal_TRAJ50_F	TAGGNYKII***FGRG***TQLIVDSS
sOncmyk_TRAJ21_F	TTGGNVKLI***FGRG***TQLIVDSS
aOncmyk_TRAJ28_F	TTGGNVKLI***FGRG***TQLIVDSS
Salsal_TRAJ96_F	TAGGGNSKMI***FGAG***TQLIVDSS
sOncmyk_TRAJ31_F	TAGGGQSKII***FGTG***TQLIVDSS
aOncmyk_TRAJ41_F	TAGGGQSKII***FGTG***TQLIVDSS
sOncmyk_TRAJ42_F	TTGGGNSKII***FGTG***TQLIVDSS
aOncmyk_TRAJ53_F	TTGGGNSKII***FGTG***TQLIVDSS
Salsal_TRAJ70_F	TTGGLSKMI***FGAG***TQLIVDSS
Salsal_TRAJ77_F	TTGGLSKMI***FGAG***TQLIVDSS
sOncmyk_TRAJ32_F	TTGGNDKII***FGTG***TKLIVDSS
aOncmyk_TRAJ42_F	TTGGNDKII***FGTG***TKLIVDSS
sOncmyk_TRAJ43_F	TTGGLDKII***FGTG***TKLIVDSS
aOncmyk_TRAJ55_F	TTGGLDKII***FGTG***TKLIVDSS
Salsal_TRAJ98_F	TTGGGLDKII***FGTG***TKLIVDSS
sOncmyk_TRAJ24_F	IGGANKII***FGTG***TKVIVNS

aOncmyk_TRAJ32_F	IGGANKII***FGTG***TKVIVNS
Salsal_TRAJ111_F	TQRANKII***FGTG***TKVIVNSSK
Salsal_TRAJ83_F	TGAANKII***FGTG***TKVIVNSS
sOncmyk_TRAJ33_F	TGAANKII***FGTG***TKVIVNSS
aOncmyk_TRAJ44_F	TGAANKII***FGTG***TKVIVNSS
Salsal_TRAJ36_F	TLGSYKII***FGTG***TKVIVNSS
sOncmyk_TRAJ50_F	TLGANKII***FGTG***TKVIVNSS
aOncmyk_TRAJ65_F	TLGANKII***FGTG***TKVIVNSS
Salsal_TRAJ56_F	TTGTDKII***FGTG***TKVVVNSS
Salsal_TRAJ34_F	FNKIL***FGTG***TKFTADLSK
Salsal_TRAJ54_F	TTDGFNKIV***FGTG***TKLKVDSS
Salsal_TRAJ108_F	TTVSKII***FGTG***TKLKVDSSK
Salsal_TRAJ84_F	NTGGQKLI***FGSG***TTVFVESS
sOncmyk_TRAJ15_F	NIGGQKLI***FGSG***TTVFVESSK
aOncmyk_TRAJ21_F	NIGGQKLI***FGSG***TTVFVESSK
aOncmyk_TRAJ33_F	NAGGQKLI***FGSG***TTVFVQSS
Salsal_TRAJ37_F	NAGGQKLI***FGSG***TTVFVESS
sOncmyk_TRAJ34_F	NTGTRTLI***FGSG***TTVFVQSS
aOncmyk_TRAJ45_F	NTGTRTLI***FGSG***TTVFVQSS
sOncmyk_TRAJ51_F	NTGTEKLI***FGSG***TTVFVQSS
aOncmyk_TRAJ66_F	NTGTEKLI***FGSG***TTVFVQSS
Salsal_TRAJ71_F	TATQKLI***FGSG***TTVFVESS
Salsal_TRAJ112_F	NTATQKLI***FGSG***TRVFVESSK
Salsal_TRAJ57_F	NTGTQKLI***FGSG***TTVFVESS
sOncmyk_TRAJ40_F	NVGTRRLI***FGSG***TTL
aOncmyk_TRAJ51_F	NVGTRRLI***FGSG***TTL
Salsal_TRAJ93_F	GTRRLI***FGSG***TTLSIQSTK
Salsal_TRAJ13_F	NDGTRRLI***FGRG***TTLSIQSS
Salsal_TRAJ66_F	NDGTQRLI***FGSG***TTLSIQSS
aOncmyk_TRAJ23_F	NDGTGRLI***FGRG***TTLSIQSSK
aOncmyk_TRAJ14_P	NDGTRGLN***FGTG***TTLSIXASSK
Salsal_TRAJ27_F	NDGTRGLN***FGTG***TTLSIQSS
Salsal_TRAJ44_F	NDGTGTGKLI***FGRG***TTLSIQSS
Salsal_TRAJ24_F	NDGTWKLI***FGGG***TQLNVESS
sOncmyk_TRAJ38_F	NDGIWNLI***FGSG***TLYIESS
aOncmyk_TRAJ49_F	NDGIWNLI***FGSG***TLYIESS
aOncmyk_TRAJ13_F	NDGTWKLI***FGGG***THLNVESS
Salsal_TRAJ90_F	GTWNLI***FGSG***TQLFIESS
Salsal_TRAJ15_F	NDGAGKLI***FGSG***TSLSIETS
sOncmyk_TRAJ18_F	NDGVRKLI***FGSG***TSLSVETS
aOncmyk_TRAJ25_F	NDGVRKLI***FGSG***TSLSVETS
Salsal_TRAJ46_F	NVGAGKLI***FGSG***TSLSIETS
Salsal_TRAJ68_F	NDGARKLI***FGSG***TSLSIETS
Salsal_TRAJ75_F	NDGARKLI***FGSG***TSLSIETS
Salsal_TRAJ29_F	YVGGEKII***FGSG***TSLFVETS
Salsal_TRAJ48_F	YAGGGKFI***FGSG***TSLFVETR
sOncmyk_TRAJ20_F	YAGGGKFI***FGSG***TSLFVKTS
aOncmyk_TRAJ27_F	YAGGGKFI***FGSG***TSLFVKTS
Salsal_TRAJ60_F	TTGAGYGKLI***FGTG***TRLLIESS
Salsal_TRAJ87_F	TSGAGYGKLI***FGTG***TRLLIESS
Salsal_TRAJ74_F	TSGAGSDKLI***FGTG***TRLLIESS
Salsal_TRAJ115_F	TSGAGSGKLI***FGTG***TRLLIESS
Salsal_TRAJ104_F	TTGVGTGKMI***FGTG***TQLTVDSS
sOncmyk_TRAJ26_F	TSGGSYGKLI***FGTG***TRLLIESS
sOncmyk_TRAJ35_F	TSGGSYGKLI***FGTG***TRLLIESS
aOncmyk_TRAJ46_F	TSGGSYGKLI***FGTG***TRLLIESS
sOncmyk_TRAJ53_F	TTGGGSSKLI***FGTG***TRLLIESS
aOncmyk_TRAJ68_F	TTGGGSSKLI***FGTG***TRLLIESS

aOncmyk_TRAJ35_F	TTGVGSSKLI***FGTG***TRLLIESS
Salsal_TRAJ117_F	TRGNSNRII***FGYG***TQMLVESGK
Salsal_TRAJ118_F	TSGNSNGII***FGNG***TQMLVESGK
Salsal_TRAJ119_F	TRGNSNRII***FGYG***TQLLVESGK
sOncmyk_TRAJ55_F	TRGNSNRLI***FGNG***TQLLVESGK
aOncmyk_TRAJ70_F	TRGNSNRLI***FGNG***TQLLVESGK
Salsal_TRAJ123_ORF	TRTSDKIT***FARG***TQLFVEKGE
aOncmyk_TRAJ74_ORF	TRTSDKIT***FARG***TQLFVEKGE
sOncmyk_TRAJ58_ORF	TRTSDKIT***FARG***TQLFVEKGE
Salsal_TRAJ126_P	VTTSEKMY***FARG***TQLFVEGE
Salsal_TRAJ121_F	NDAGTKII***FGRG***TRLLVLSSK
sOncmyk_TRAJ57_F	NDGSTKII***FGRG***TRLLVLSSK
aOncmyk_TRAJ72_F	NDGSTKII***FGRG***TRLLVLSSK
sOncmyk_TRAJ60_F	NDAGTKII***FGRG***TRLLVLSGK
aOncmyk_TRAJ76_F	NDAGTKII***FGRG***TRLLVLSGK
Salsal_TRAJ4_F	NNNFNKII***FGRG***TKCGVLSS
Salsal_TRAJ17_F	NSNLNKIT***FGSG***TKVVVLSS
Salsal_TRAJ7_F	NNNNFKTI***FGSG***TKVVVLSR
Salsal_TRAJ16_F	NTNNIKII***FGFG***TKLVVLSS
Salsal_TRAJ28_F	SVGVRVV***FGRG***TKLTVNPS
sOncmyk_TRAJ10_F	TVGVRVV***FGHG***TKLTVNPS
aOncmyk_TRAJ15_F	TVGVRVV***FGHG***TKLTVNPS
sOncmyk_TRAJ19_F	DFGVNRVV***FGHG***TMLTVNSCK
aOncmyk_TRAJ26_F	DFGVNRVV***FGHG***TMLTVNSCK
Salsal_TRAJ47_F	LLESTEL***FGHG***TMLTVNSCK
Salsal_TRAJ59_F	TVGQKLV***FGKG***TMLTVSTG
Salsal_TRAJ86_F	TVGQKLV***FGKG***TMLTVSTGK
Salsal_TRAJ114_F	VTVGQKLV***FGKG***LKLTVSTG
Salsal_TRAJ73_ORF	TVGQKLV***FGKR***NNVNCINR
sOncmyk_TRAJ27_F	TDGRNKIY***FGLG***SKVIVQP
aOncmyk_TRAJ37_F	TDGRNKIY***FGLG***SKVIVQP
Salsal_TRAJ63_F	NNGRNKIY***FGLG***SKVIVQPG
Salsal_TRAJ22F	NNGRNKIY***FGAG***IKVIIQPG
aOncmyk_TRAJ11_F	NNGWNNIY***FGEG***IKVIIQPG
Salsal_TRAJ10_ORF	MNDGWNKIY***FRQD***TQFIFQPG
sOncmyk_TRAJ9_F	NDIWNKVY***FGLG***TCSARLKLYE
aOncmyk_TRAJ9_F	NDIWNKVY***FGLG***TCSARLKLYDIYQ
Salsal_TRAJ20_F	YMEQVY***FGLG***TCSARLKL
Salsal_TRAJ62_ORF	TDGWNKIY***FGHR***LAYCSAR
Salsal_TRAJ38_P	SLTQVSKNTFKVRLIIVE
Salsal_TRAJ43_ORF	EPRNSY***FDLE***LNYSLRQMN
Salsal_TRAJ42_F	LWRQTHI***FGLG***TKMICTKK
Salsal_TRAJ6_ORF	LVEQESINFI***SGAG***IKITIDTS
Salsal_TRAJ8_ORF	YGIIT***FVSV***TKLIIEQNL
Salsal_TRAJ52_ORF	TSANKLI***FGHV***SVQHNIIKTST
aOncmyk_TRAJ61_F	TSANKLI***FGQG***IYFFYFFLFYLYLT
