# Supplementary material for: Profiling the T Cell Receptor Alpha/Delta Locus in Salmonids
Source: Front Immunol. 2021 Oct 18;12:753960. doi: 10.3389/fimmu.2021.753960 (PMC8559430; doi:10.3389/fimmu.2021.753960)
Supplement: Supplementary file 1 [file DataSheet_1.zip › all supplementary files/Supplementary data 4.pdf]

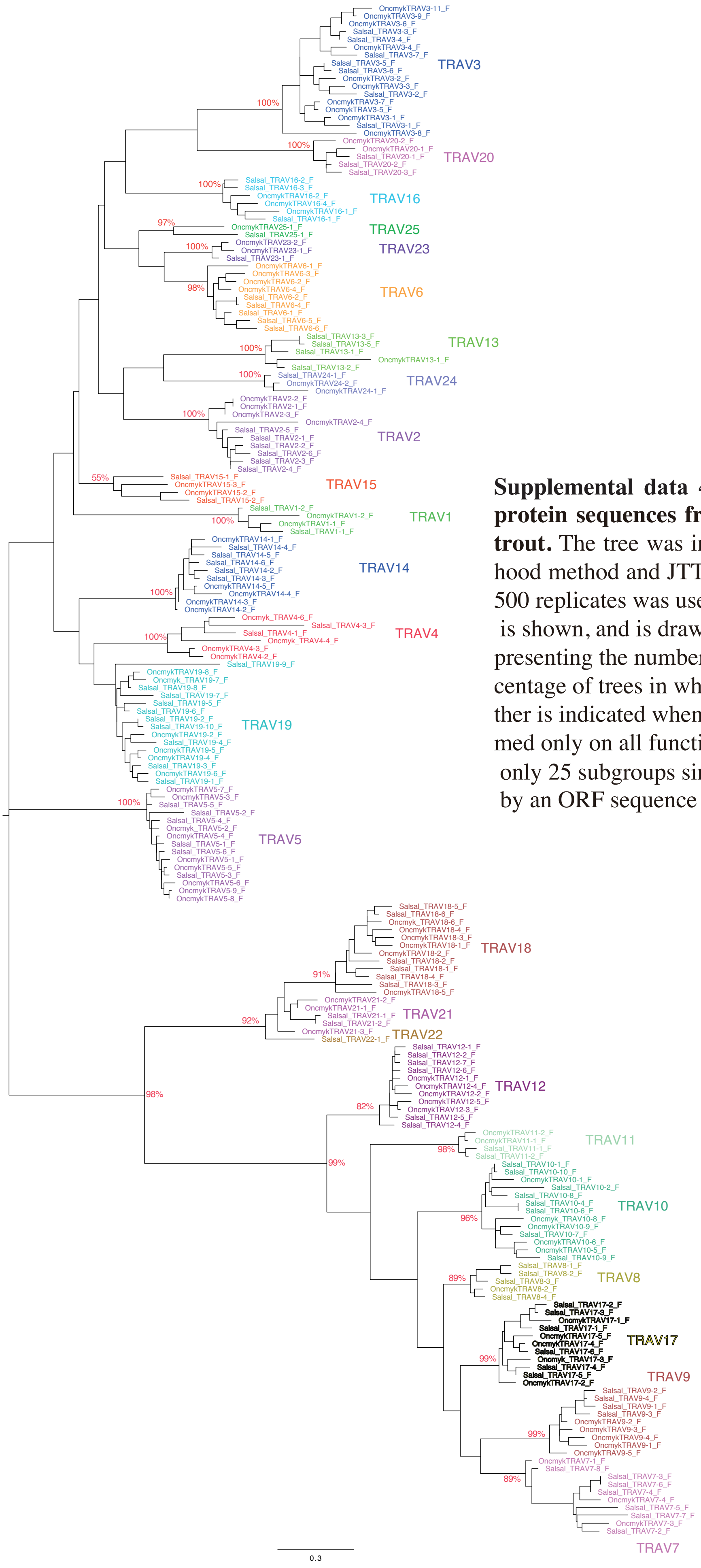

Supplemental data 4. Evolutionary tree of TRVA/D protein sequences from Atlantic salmon and rainbow trout. The tree was inferred using the Maximum Likelyhood method and JTT matrix based model. A boostrap of 500 replicates was used. The tree with highest likelihood is shown, and is drawn to scale, with branch lengths representing the number of substitutions per site. The percentage of trees in which the asscoiated taxa cluster together is indicated when >80. The analysis has been performed only on all functional V sequences. It comprises only 25 subgroups since subgroup 26 is only represented by an ORF sequence in the Arlee rainbow trout genome.
