# Supplementary material for: Profiling the T Cell Receptor Alpha/Delta Locus in Salmonids
Source: Front Immunol. 2021 Oct 18;12:753960. doi: 10.3389/fimmu.2021.753960 (PMC8559430; doi:10.3389/fimmu.2021.753960)
Supplement: Supplementary file 1 [file DataSheet_1.zip › all supplementary files/Supplementary data 5.pdf]

A.

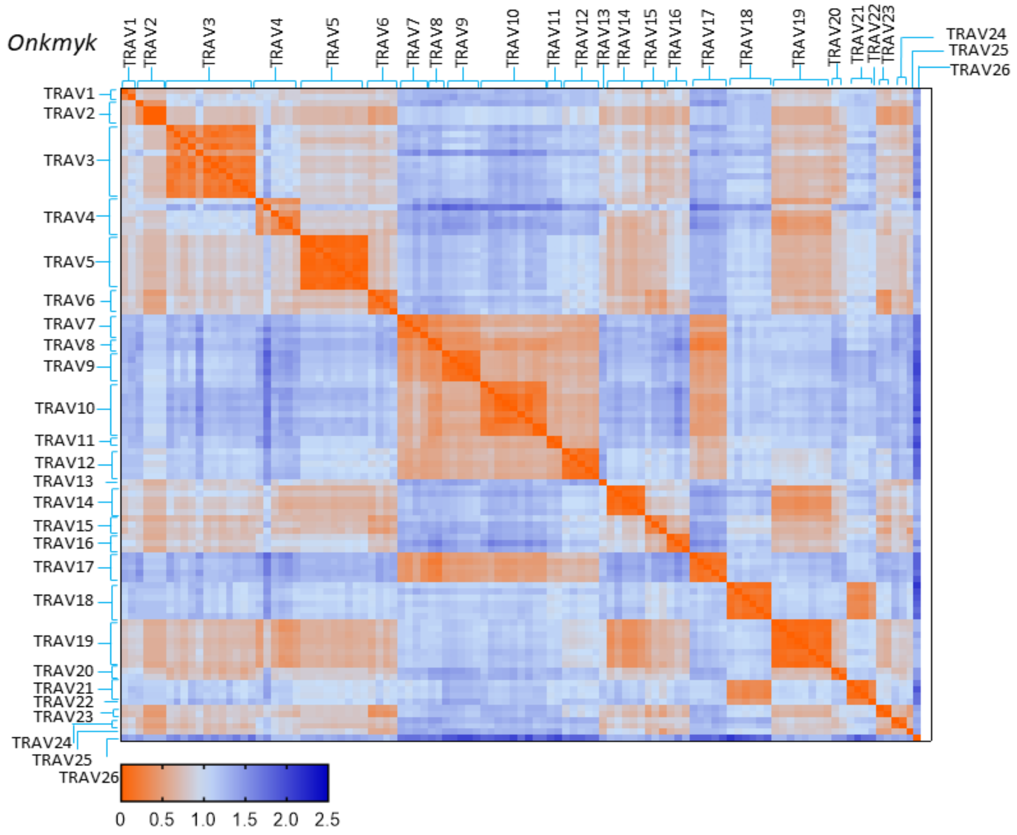

B.

```
                    1        10    FR1    20 23   27   CDR1   38    41      FR2       55  56  CDR2  65  66   70          80    FR3   90              104   CDR3
                    |........|.....  ....|..|...  |..........|  ..|.....  ...|....|  |.........|  |...|....  .....|....  .....|......  .......|............
aOncmyk_TRAV1-1[a]  CEDLTPLKKEEYCLE GTPVTLSYNYS RTAT....AGDE FHWYRQDT AQRPEFLLY ILG....SGF INKADSLNT RISAKLNKEK NRLDLEISSALV TDSALYYCAVR---------
aOncmyk_TRAV2-1     EEEIISATTKERVFE GGGITLSCNYT GSYS.....SDS LLWYQQYP RSKPEFLLL ITE....AGL KIHNESIHP RLSVKLNEEK THVDLEISSAEV TDSALYYCALR---------
aOncmyk_TRAV3-1     EQVLTPYTDVEVASE RDRVTLSCNYT SG........DT LQWYRQYP KSAPQLLIN .......... EYMDNT--P GFSLNHDKKA KRVDLVISSAHV TDSAMYYCALR---------
aOncmyk_TRAV3-2     EQILTPYTDVEVASE RDRVTLSCNYT SSV.......NT LLWYRQYP KSAPQLLVK .......... EYADIT--P GFTLNHDNNV KRMDLEISSAEV TDSALYYCALK---------
aOncmyk_TRAV3-6     EQVLTPYTDVEVASE RERVTLSCNYS SGVS....SGVT LQWYRQYP QSAPLLLVM .......... EHMLAKT-P GFLLNHDKKA KRVDLEISSAQV TDSALYYCALQ---------
aOncmyk_TRAV4-2     LQTIQPNQHEVYGED GSNVKLSCICS SA........YS VLWYKQYP GSVPQYLLL IHHA...SRT VVRAKPLYP RIPVKLNKEK NCTDLEISSVEV KYSALFYCVVT---------
aOncmyk_TRAV4-5     QQTSQPNQHEVYGEE GGNVQLSCNYS SL........YR VYCGINSI QYQFPNFLL FILL.ASRTV VRARPPNLL IPVKLNKDKM CADLEISSAEVR LLCSVLLCCDV---------
aOncmyk_TRAV4-6[e]  LQTVQPNQ--VYGEE GSNVQLSCNYS SA........YS VLWYKQHP GSIPQFLLF IHHA...SRT VVRAKPPYS HLTGILNKEK TRTDLEISSVEV RDCSVRLCCK----------
aOncmyk:TRAV5-1     QETITPVKPEVNALQ GTDITLSCNYK GKV.......NN LQWYRQYP GSRPECLLW ILPT...NKN VQNTSITTP RHTGGLNEEK TRVDLMISSVEL EDSALYHCALQ---------
aOncmyk_TRAV5-9     QETITPVKPEVNALQ GTDITLSCNYK GNI.......FN LQWYRQYP GSRPECLLW ILPT...NKY AQNTSITTP RHTGRLNEEK TRVDLMISSVEL EDSALYHCAL----------
aOncmyk_TRAV6-1[b]  GNDITLTTETVVGME GDVLKLSSHYS SA........ST LQWFQQYP GSSPIFLLL TELS..SNLS VVNANSPYL RLSVKLNKER TRVDLEISSAVV TDSALYYCALE---------
aOncmyk_TRAV7-1     ADHVQQQPGGVIATE GGLVTLSCQYN TSAN.....NAY LYWFKQEE NDFPKYMLS RYS....FGS GDNATGFKE RFDARLDADS KSVPLTIQRVQL SDSAVYYCALQ---------
aOncmyk_TRAV7-3     GDNVQQQPGSVIATE GGLVTLSCQYN TSAT....NFAY LYWYKQEA NDFPKQMLS RFS....TGS GDNAAGFKE RFNASLNAKT QSVPLTIQRLQL SDSAVYYCALR---------
aOncmyk_TRAV7-4     GDHVQQQPGGVIATE GGLVTLSCQYN TSAN....NDVY LFWFKQEA NDFPKHMLR H........Y SFGSAGFKE RFNASLNAKT QSVPLMIQRVQL SDSVVYYCALR---------
aOncmyk_TRAV8-2     EDSVIQPPGDVIATE GEQVTLDCQFD TLDT.....NPY LFWYKQGA NDFPKYMLR RATFGSDNAI EFLGRFDAH LNLTTLKSAS KSFPLTIQRLQL SDSAVYYCALR---------
aOncmyk_TRAV9-1     GDRVLQPKGVEMAAE GGQITLVCHYE TDDP.....SPY LFWYKQGA NDFPKYMLL RQK....FGT GDNSTEFKE RFHADLDANS KSVPLTIQSVQL FDSAVYYCALR---------
aOncmyk_TRAV10-1    EESVIQPTEDVEVVE GQPTQLSCTFD TTDL.....SPY LFWYKQQA NGKPMFMLR EDT....FGT GETATEFKE RFHARLNVTA KSVPLTIQRLQL SDSAVYYCALR---------
aOncmyk_TRAV10-3    EENVTQPTEDVMVVE GQPTTLTCLFD TTNQ.....SPY LFWYKQLA NGKPIFMLR RDI....FSP GESATEFKD RFDAHLNVTA KSVPLTIQRGQL SDSAVNYCALRPIVTAGY--
aOncmyk_TRAV10-6    EENVRQPTEDVMVVE GHPTTLTCLFD TSQ......SPY LFWYKQLA NGKPIFMLR RDA....FSP GESSTEFKD RFDAHLNVSG KSVPLTIQRVQL SDSAVYYCALR---------
aOncmyk_TRAV11-1    ADGVTQSTREVTAYE GEDVSLNCAYN TSLS.....APS LFWYIQYT NDYPEYVLR KDV....IGP GDIDDRFKE RFDAHLSSIT KLVPLTIQRVQL SDSAVYYCALK---------
aOncmyk:TRAV12-1    EEAVDQQSGHVTALE GGLVTLSCNYT TSSP.....SPD LFWYIQLT RDTPQYVLR RDR....YSE GSNSDKFKK RFDSRMNFTS SSVPLTIQRLQL SDSAVYYCALR---------
aOncmyk_TRAV13-1    VKFFCRKQFCFFNKP CYYVILSCKFD GNV.......YN LQWYRQYP RSKPEFLLY ITP....VGL AFTATPPPP CLTVSIDKVE KCVDLKISSAVV TDSALYYCALM---------
aOncmyk_TRAV14-1    GQTIEPVREELCAPA GSNITLLCTYS SA........VS LQWYLQDP GSPPQYILL ILHG...VGS PSRAPGLDP RLSVKLNDEK NRVDLEISSAKE KDSAMYYCALM---------
aOncmyk_TRAV15-2    EDDITPTSPEEYYLE GSRVKISCNYT VKS.......DS LLWYRQYS GSGLQFLYL VTTT..NNPY DVRGDPQDF RLTVTQNKER TRVDLEISSVEE TDSALYYCALR---------
aOncmyk_TRAV16-2[f] GDELFSNSL-EDVIH GGSVKLSCNYT VSG......GGS VLWYHQYH TSAPQFLLL LSVYSASAET IVTADSPYP RLSVNVDKVT DCVDLEISSAEV TDSAL-YCYALR--------
aOncmyk_TRAV16-4    GDGISSNSLEEDVIH GGSVMLSCNYT VSG......GGS VLWYHQYH TSAPQFLLL LSLYSASAET KVTADPPYP RLSVNVDKVA ERVDLKVSSAEV TDSALYYCALK---------
aOncmyk_TRAV17-1    EDTVIQPPGDVTATE GGQVTLDCQFD AVNT....NNLY LFWYKHEL HGFPKFMLG LSF.....FR STNATGYEE RFDAHLDKDS KSVPLTIQRVQL SDSAVYYCALK---------
aOncmyk_TRAV18-1    GDSVTPDKEEYTPTE GSSVSLRCTYE TKRS.....NIY LYWYRHYC NQAPQFLLY KG......AR SLSSDEHIP DKRYKSTTSR TSTELVITSLTL ADTALYYCAV----------
aOncmyk_TRAV18-2    ADSVTPDKKEYTPTE RSSVSLSCTYE TSSE.....FIY LYWYRHYP NQAPQFLLY KG......AR SWSSDEHIP DKRYTSTTSR TSTELVITNLTL ADTALYYCALREY-------
aOncmyk_TRAV19-2    EQTIEPNQHEVYAEV GINVLLSCNYS SA........DS LLWYKQSP GAAPQYLLL IQHY...TGT EHRADSLDS RVSGKLNKER TRVDLEISSAEE TDSALYYCALR---------
aOncmyk_TRAV20-1    EDIISPDRDVVDDMD GCSVQLSCRYN SSL......TNS LLWYLQHP GSSPQFLIV DY.......S GIITNTDST QWTLIHEEED NRVDLDISSTEL TDSALYYCTLK---------
aOncmyk_TRAV21-2    GDHITPVNNEVISTE GELVTLRCSYD TSYE.....NIW LYWYRHYP KQAPQFLLY KG.......A RSWSSEHIS DKRYKPTTSR TSTELVITSLTL ADTALYYCARRDD-------
aOncmyk_TRAV21-1    GDHITPVNNEVISTE GELVTLRCSYD TSYE.....NIW LYWYRHYP NQAPQFLLY KG......AR SQSSEEHIP DKRYTSTTSR TSTELVITSLTL ADTALYYCAV----------
aOncmyk_TRAV22-1    GEHITAVQHEVISTE GKPVILRCSYD ASWN.....YPL LYWYRHYD YQAPHFLLY KG.......A RSSTREHIP DKRYTSKTSQ TTTELIITHLTL ADTALYYCALRDCYYENCY-
aOncmyk_TRAV23-1    HDEIQPTAKIMYGDK GGFVTLSCNYS GSP.......YN LQWYRQYP RSAPKFLLL TTVS..SNPS VVNATPPYP HLSVKLNKER THLDLEISSAVV TDSALYYCCALQ--------
aOncmyk_TRAV24-1    EDYINPTRSMVGGVE GDTITLSCSYI GSV.......NN LHWYRQYL RSKPEFLFL I........T KSGYVQKPV TPRMSAQVQT DRVDLEISSAEV TYSTLYYCSLR---------
aOncmyk_TRAV25-1    ENDIKPASETEHGME ECSVTLSCNFS GSV.......DY LLWYRHYP RSAPKFLLY TTVT..LKPS VFRAEPDDP RLSVNLKKER TCVDLKSSSAEV TDSSLYYCVLK---------
```

Supplementary data 5. Comparison of TRAV sequences from rainbow trout. (A) Heatmap representation of pairwise distance matrix of functional and ORF TRAVs. Pairwise distances were estimated from amino-acid alignments using the Poisson model. 94 functional TRAVs and 14 ORF (TRAV3-12, TRAV4-1. TRAV7-2, TRAV8-1, TRAV10-2, TRAV10-3, TRAV10-4, TRAV10-7, TRAV15-1, TRAV16-3, TRAV19-1, TRAV19-3, TRAV22-1 and TRAV26-1) derived from the Arlee genome were included in the analysis. Color in each pixel indicate pairwise distance among and between each TRAV sequence. (B) Multiple Alignment of representative TRAV subgroups for Atlantic salmon (Salsal). The CDR1 and CDR2 encoded amino acids are colored as follows: gray positively charged R groups, blue negatively charged R groups, green aromatic R groups, pink polar uncharged R groups, yellow nonpolar R groups, red conserved cysteines (1st-Cys 23 and 2nd-Cys 104) and conserved tryptophan 41. [a]Conserved Cys 23 is a Tyrosine (Y). [b] Conserved Cys 23 is a Serine (S). [e] two amino acid deletion in FR1 compared to the other genes of the subgroup. [f] one amino acid deletion in FR1 and deletion of a Y in position 103 compared to the genes of the subgroup.
