# Supplementary material for: Profiling the T Cell Receptor Alpha/Delta Locus in Salmonids
Source: Front Immunol. 2021 Oct 18;12:753960. doi: 10.3389/fimmu.2021.753960 (PMC8559430; doi:10.3389/fimmu.2021.753960)

Supplementary data 2. Organization of TRA/TRD locus in rainbow trout (Onchorynchus mykiss, Swansson strain). TRV gene names are according to IMGT nomenclature. Functional and ORF TRAV genes are in red. TRAV pseudogenes with frameshift(s) in the V-REGION are in grey and their name are not displayed. The arrow indicates the orientation. The symbols representing the genes are not to scale.

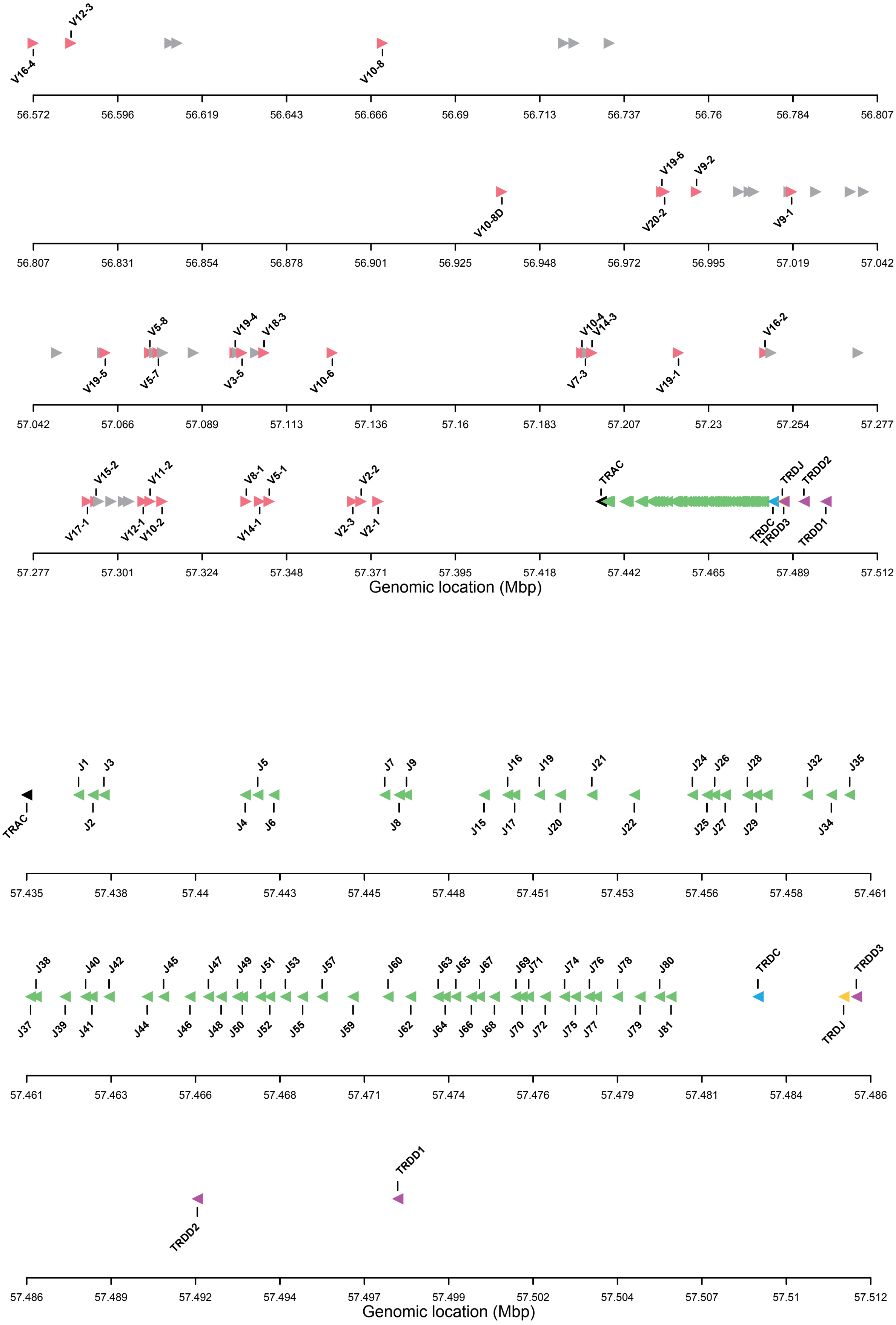

Supplement: Supplementary file 1 [file DataSheet_1.zip › all supplementary files/Supplementary data 2.pdf]
