# Supplementary material for: Profiling the T Cell Receptor Alpha/Delta Locus in Salmonids
Source: Front Immunol. 2021 Oct 18;12:753960. doi: 10.3389/fimmu.2021.753960 (PMC8559430; doi:10.3389/fimmu.2021.753960)
Supplement: Supplementary file 1 [file DataSheet_1.zip › all supplementary files/Supplementary data 8.pdf]

Supplementary Data 8.

A) Nucleotide and deduced amino acid sequences of TRDJ genes identified in Atlantic salmon (Salmo salar) and in two rainbow trout (Oncorhynchus mykiss) strains: Arlee (Oncmyk_Ar) and Swanson (Oncmyk_Sw). Each J-GENE-UNIT comprises the J-RS (J-NONAMER, J-SPACER '12-spacer', J-REGION)

>Salsal_Chr14_NC_027313.1_TRDJ1_F_J-GENE-UNIT_21205173_21205092

```
                               D  K  L  T  F  G  K  A  I  N  L  I  V  E  P
GGTTTTTGTACTGACATGTTCCACAGTGCAGATAAACTGACGTTTGGAAAAGCTATCAATCTCATTGTTGAACCCA
```

>Oncmyk_Ar_Chr8_NC_048572.1_TRDJ1_F_J-GENE-UNIT_61991792_61991717

```
                               A  K  L  T  F  G  K  A  I  N  L  I  V  E  P
GGTTTTTGTATGGACATGTTCCACAGTGCAGCTAAACTGACATTTGGAAAAGCCATCAATCTCATTGTTGAACCCA
```

>Oncmyk_Sw_Chr8_NC_035084.1_TRDJ1_F_J-GENE-UNIT_57485693_57485618

```
                               A  K  L  T  F  G  K  A  I  N  L  I  V  E  P
GGTTTTTGTACAGACATGTTCCACAGTGCAGCTAAACTGACATTTGGAAAAGCCATCAATCTCATTGTTGAACCCA
```

B) Nucleotide sequences of TRDD genes and RS sequences (TRDD D-REGION are grey highlighted). Each D-GENE-UNIT comprises the 5'D-RS (5'D-NONAMER, 5'D-SPACER '12-spacer', 5'D-HEPTAMER), the D-REGION and the 3'D-RS (3'D-HEPTAMER, 3'D-SPACER '23-spacer', 3'D-NONAMER).

```
                              5'D-RS             D-REGION                    3'D-RS
                     9        12-spacer     7                      7         23-spacer            9
Salsal     TRDD3 GGTTTTTGTGCTACGTTTTCTCACAGTGTGGGGTGGGTTACCACAGTGACTGAATATTCATGTTGCGTTGTACAAAAACC
Oncmyk_Ar  TRDD3 GGTTTTTGTGCTACGTTTTCTCACAGTGTGGGTTGGGATACCACAGTGACTGAATATTCATGTCGTGTTGTACAAATACC
Oncmyk_Sw  TRDD3 GGTTTTTGTGCTACGTTTTCTCACAGTGTGGGTTGGGAGACCACAGTGACTGAATATTCATGTCGCGTTGTACAAAAACC
```

	5′D-RS			D-REGION	3′D-RS		
	9	12-spacer	7		7	23-spacer	9
Salsal_TRDD2	***AGTTTTTGC***	TGAGGGGTATTT	***CAGTGTG***	TGGATTGGGATAC	***CACATTG***	ATTCAAACTAGGTATGTACCTGA	***ACAAAAACT***
Oncmyk_Ar_TRDD2	***AGTTTTTGC***	TGAGGGGTATTT	***CAGTGTG***	GGGATTTGGGATAC	***CACATTG***	ATTCAAACTAGGTATGAACCTGA	***ACAAAAACT***
Oncmyk_Sw_TRDD2	***AGTTTTTGC***	TGAGGGGTATTT	***CAGTGTG***	GGGATTTGGGATAC	***CACATTG***	ATTCAAACTAGGTATGAACCTGA	***ACAAAAACT***
Salsal_TRDD1	***AGTTTTTGC***	TGAGGGGTATTT	***CAGTGTG***	TGGACTAC	***CACATTG***	ATTCAAACTAGTTATGTTCCTGA	***ACAAAAACA***
Oncmyk_Ar_TRDD1	***AGTTTTTG***	CTGAGGGGTATTT	***CAGTGTG***	TGGACTAC	***CACATTG***	ATTCAAACTAGTTATGTTCCTGA	***ACAAAAACA***
Oncmyk_Sw_TRDD1	***AGTTTTTG***	CTGAGGGGTATTT	***CAGTGTG***	TGGACTAC	***CACATTG***	ATTCAAACTAGTTATGTTCCTGA	***ACAAAAACA***
