# Supplementary figures and images for: Profiling the T Cell Receptor Alpha/Delta Locus in Salmonids
Source: Front Immunol. 2021 Oct 18;12:753960. doi: 10.3389/fimmu.2021.753960 (PMC8559430; doi:10.3389/fimmu.2021.753960)

A

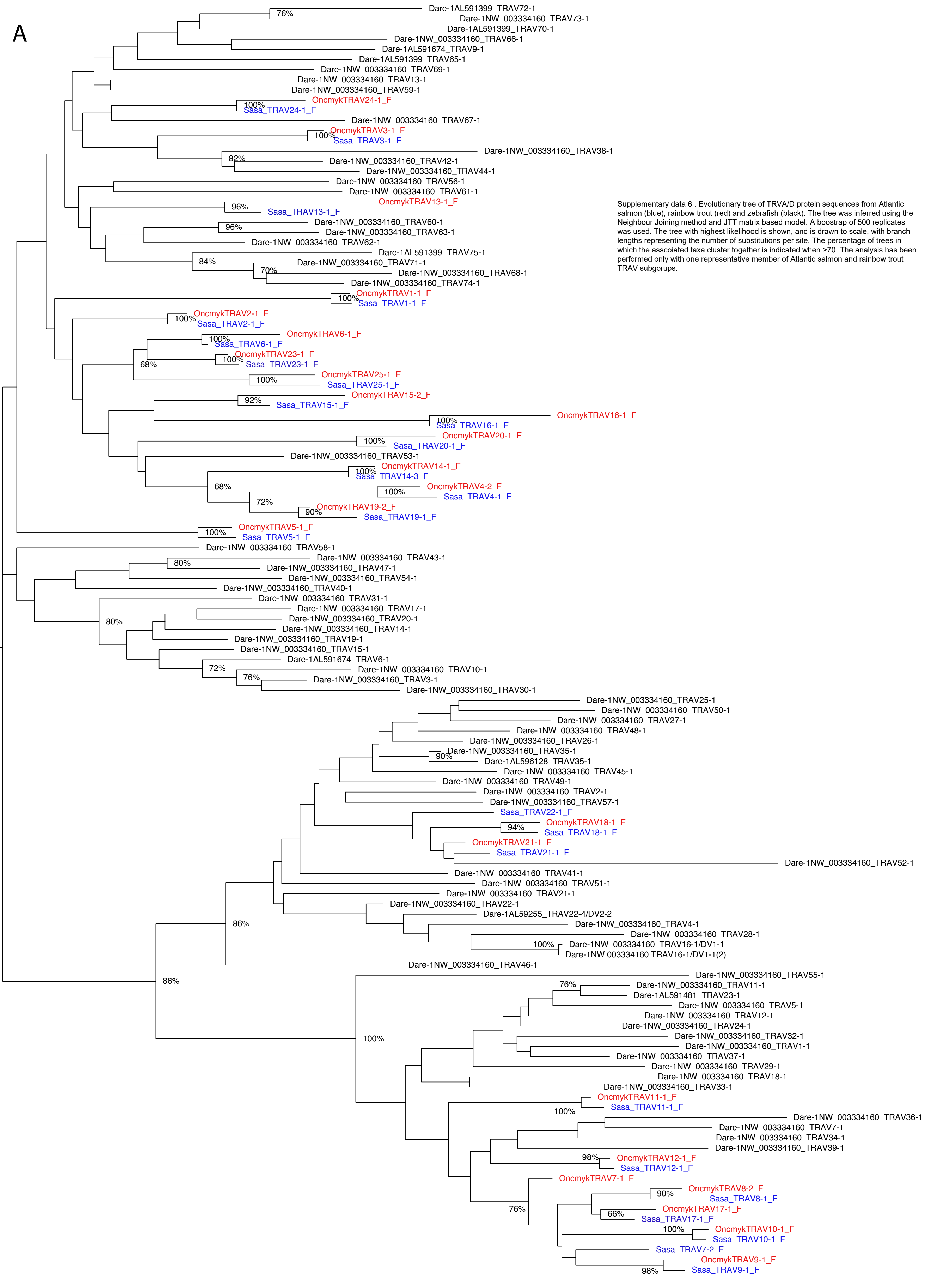

Supplement: Supplementary file 1 [file DataSheet_1.zip › all supplementary files/Supplementary data 6.pdf]
